# Reporting on patient’s body mass index (BMI) in recent clinical trials for patients with breast cancer: a systematic review

**DOI:** 10.1186/s13058-024-01832-7

**Published:** 2024-05-22

**Authors:** Josephine Van Cauwenberge, Karen Van Baelen, Marion Maetens, Tatjana Geukens, Ha Linh Nguyen, Ines Nevelsteen, Ann Smeets, Anne Deblander, Patrick Neven, Stijn Koolen, Hans Wildiers, Kevin Punie, Christine Desmedt

**Affiliations:** 1 Laboratory for Translational Breast Cancer Research, Department of Oncology, KU Leuven, Herestraat 49, Box 808, 3000 Louvain, Belgium; 2 Department of Gynecological Oncology, University Hospitals Leuven, Leuven, Belgium; 3 Department of General Medical Oncology, University Hospitals Leuven, Leuven, Belgium; 4 Department of Surgical Oncology, University Hospitals Leuven, Leuven, Belgium; 5 Department of Medical Oncology, Erasmus MC Cancer Institute, Rotterdam, The Netherlands; 6 Department of Hospital Pharmacy, Erasmus MC, Rotterdam, The Netherlands; 7 Department of Medical Oncology, GZA Hospitals Sint-Augustinus, Wilrijk, Belgium

**Keywords:** Obesity, Breast cancer, Body mass index (BMI), Clinical drug trials, Dosing, Treatment efficacy

## Abstract

**Background:**

The proportion of patients with breast cancer and obesity is increasing. While the therapeutic landscape of breast cancer has been expanding, we lack knowledge about the potential differential efficacy of most drugs according to the body mass index (BMI). Here, we conducted a systematic review on recent clinical drug trials to document the dosing regimen of recent drugs, the reporting of BMI and the possible exclusion of patients according to BMI, other adiposity measurements and/or diabetes (leading comorbidity of obesity). We further explored whether treatment efficacy was evaluated according to BMI.

**Methods:**

A search of Pubmed and ClinicalTrials.gov was performed to identify phase I-IV trials investigating novel systemic breast cancer treatments. Dosing regimens and exclusion based on BMI, adiposity measurements or diabetes, documentation of BMI and subgroup analyses according to BMI were assessed.

**Results:**

495 trials evaluating 26 different drugs were included. Most of the drugs (21/26, 81%) were given in a fixed dose independent of patient weight. BMI was an exclusion criterion in 3 out of 495 trials. Patients with diabetes, the leading comorbidity of obesity, were excluded in 67/495 trials (13.5%). Distribution of patients according to BMI was mentioned in 8% of the manuscripts, subgroup analysis was performed in 2 trials. No other measures of adiposity/body composition were mentioned in any of the trials. Retrospective analyses on the impact of BMI were performed in 6 trials.

**Conclusions:**

Patient adiposity is hardly considered as most novel drug treatments are given in a fixed dose. BMI is generally not reported in recent trials and few secondary analyses are performed. Given the prevalence of patients with obesity and the impact obesity can have on pharmacokinetics and cancer biology, more attention should be given by investigators and study sponsors to reporting patient’s BMI and evaluating its impact on treatment efficacy and toxicity.

**Supplementary Information:**

The online version contains supplementary material available at 10.1186/s13058-024-01832-7.

## Introduction

Over the last decade the proportion of women with overweight and obesity, reflected by an elevated body mass index (BMI equal or above 25 and 30 kg/m^2^, respectively), has been increasing worldwide [1]. Consequently, the proportion of women with breast cancer (BC) who are overweight or obese is increasing as well. Obesity is associated with a higher prevalence of postmenopausal breast cancer, more advanced disease burden at diagnosis and worse survival [2–4]. Retrospective analyses show that obesity is also associated with an increased risk of distant recurrence in patients with both estrogen receptor (ER)-negative and ER-positive disease after adjustments for standard clinicopathological parameters [5].

Different approaches are used for drug dosing related to weight and/or length parameters. Fixed dose administration minimizes the risk of mistakes and maximizes the efficacy of the drug preparation. Weight-based approaches, on the other hand, include regimens using the patient’s actual weight, body-surface area (BSA) or, in research setting, lean body weight [6, 7]. By using the actual weight to calculate drug dosing, the dosage is increased linearly with the patient’s weight [6]. The BSA resembles the two-dimensional surface area of the skin and BSA correlates with the basal metabolic rate [8]. However, different BSA formulas are rather inconsistent in obese patients [9, 10]. Lean body weight dosing, excluding the fat tissues, correlates more closely with the actual drug clearance in hydrophilic drugs, however, this is difficult to use in clinical practice [11]. These considerations are relevant since it is known that obesity can influence pharmacokinetics and -dynamics in a drug-specific manner [12]. Both fat and lean mass increase with higher body weight, but the ratio changes, resulting in a higher fat percentage and a lower lean body percentage [13]. Obesity impacts pharmacokinetics in all facets: absorption, distribution, metabolism, and clearance [12, 14, 15]. The effect of obesity on absorption and distribution is highly drug-specific and depends on the lipophilicity of the drug [14]. Greater lipophilicity enhances the drug's capacity to cross the lipid bilayer by passive diffusion; however, it concurrently augments the drug's propensity to accumulate in adipose tissue distinct from the target organ. Examples of established chemotherapeutic drugs that are lipophilic are docetaxel, paclitaxel, adriamycin, and cisplatin [17]. A retrospective analysis of the BIG 2–98 trial demonstrated that survival was worse in patients with overweight or obesity receiving a docetaxel-based regimen, as compared to lean patients receiving the same treatment, while no difference in survival was observed according to BMI in patients receiving anthracyclines and CMF (cyclophosphamide, methotrexate, fluorouracil) [18]. The impact of obesity on pharmacokinetic properties is dependent on the therapeutic window, which is generally broader for immunotherapy and targeted therapies [19–21].

But BMI does not only impact treatment efficacy through the pharmacokinetic properties of the drugs [22, 23]. For instance, some studies have shown a potential association between obesity and a higher rate of endocrine resistance in the adjuvant setting [23–25]. This could be explained through the impact of obesity on adipocytes, metabolic markers and inflammation [23]. Controversially, overweight and obesity might be associated with an increased anti-proliferative response to neoadjuvant treatment for BC with aromatase inhibitors [26]. Experimental studies and retrospective analyses from clinical trials in some cancer types suggest that obese patients might benefit more from immune checkpoint inhibitors as compared to lean patients, although results for patients with BC are limited [27]. Finally, several studies have also revealed the impact BMI has on tumor cells and the tumor microenvironment [28–30]. Globally, these studies have shown that in some tumor types there is an increased molecular aging with obesity, slight differences in genomic landscape, and a reorganization of the composition and interactions in the tumor microenvironment.

Altogether, this collective evidence emphasizes the need to investigate the impact of obesity on pharmacokinetics and efficacy in clinical drug trials, especially given the expansion of the BC therapeutic landscape in the last decade with the emergence of novel selective estrogen receptor degraders (SERDs), CDK4/6 inhibitors, immune checkpoint inhibitors (ICIs), antibody–drug conjugates (ADCs), PI3K and tyrosine-kinase inhibitors, PARP-inhibitors as well as other targeted therapies. For most of these therapies, it is unknown whether efficacy or associated side effects could differ according to patient adiposity. Here, we conducted a systematic review on recent clinical drug trials to document the dosing regimen of these recent drugs, the reporting of BMI (or alternative body composition measurements) and the possible exclusion of patients according to BMI (or patients with diabetes) in phase I-IV trials.

## Methods

**Fig. 1:**
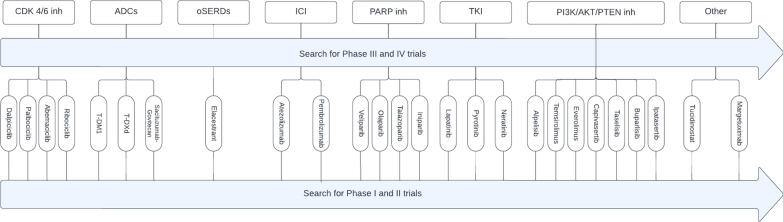
Flowchart of the search methodology

PRISMA (Preferred Reporting Items for Systematic Reviews and Meta-Analyses) guidelines were followed to conduct the literature search and study). Based on recent literature, 8 different categories of novel BC treatments of interest were identified [33–35]: CDK4/6 inhibitors, ADCs, oral SERDs, PARP inhibitors, tyrosine kinase inhibitors (TKI), ICIs, PI3K/AKT/PTEN inhibitors and other drugs under investigation such as tucidinostat and margetuximab. Two reviewers (JVC and KVB) independently searched the PubMed database and the clinicaltrials.gov database to identify phase III and IV clinical trials on these 8 novel BC drug categories by screening titles and abstracts. Additionally, a second search was conducted on phase I and II trials for those drugs within the predefined drug categories reported in phase III and IV trials (Fig. 1). Medical subject heading terms (MeSH) related to the treatments and treatment categories were used as well as names of individual drugs in the search together with the MeSH-term ‘Breast Neoplasms’ (Additional file 1: Supplementary Table 1). We included all phase I-IV trials that had a manuscript of the primary analysis accessible on the 31st of December 2023. There were no further restrictions based on date of publication. Solely manuscripts in English were included. The search terms and definitions can be found in the supplementary material (Additional file 1: Supplementary Table 1). In case of multiple records on the same trial, only the primary analyses was considered. An exception was made for basket trials investigating multiple drugs for which 1 record per study arm was allowed. Pooled analyses using results of multiple trials were excluded as well as trials that were not investigating the efficacy of drugs associated with the used search term (records off topic). Disagreements between the reviewers were resolved through discussion and consensus.

We first reported the BC drug dosing regimens in the included trials. The manuscripts were then assessed for the documentation of mean or median weight of the study population, for distribution of BMI and for potential subgroup analyses based on BMI/weight categories. BMI is defined as body mass (kg) divided by the square of the body height (m^2^). According to the WHO classification, BMI is categorized into underweight (≤ 18.5 kg/m^2^), lean (> 18.5 kg/m^2^ and < 25 kg/m^2^), overweight (≥ 25 kg/m^2^ and < 30 kg/m^2^) and obesity (≥ 30 kg/m^2^). We investigated whether there were exclusion criteria related to BMI or diabetes and whether other measurements of adiposity such as body composition or body measurements were reported. We investigated diabetes since it is the leading cause of BMI-related disability adjusted life years [31], 60% of patients who are diagnosed with diabetes mellitus type 2 are obese [32]. We further explored whether treatment efficacy and treatment-associated side effects were evaluated according to BMI. All supplementary material, if available, was also evaluated. Additionally, with a secondary PubMed search, we assessed the availability of additional retrospective analyses of the included clinical trials, that evaluated the impact of BMI and other adiposity measurements on pharmacokinetics or survival.

The names and classes of the drugs as well as the names of the different trials were used together with the MeSH-terms ‘Obesity’, ‘Body Mass Index’, ‘Body Weight’ or ‘Adiposity’ to see if any additional analyses had been done. In this stage, pooled analyses of the included trials were also searched to see if they reported on sub-analyses.

Results are shown in a descriptive manner.

## Results

### Selection of the trials investigating new breast cancer treatments

1. Primary search: phase III and IV trials

**Fig. 2:**
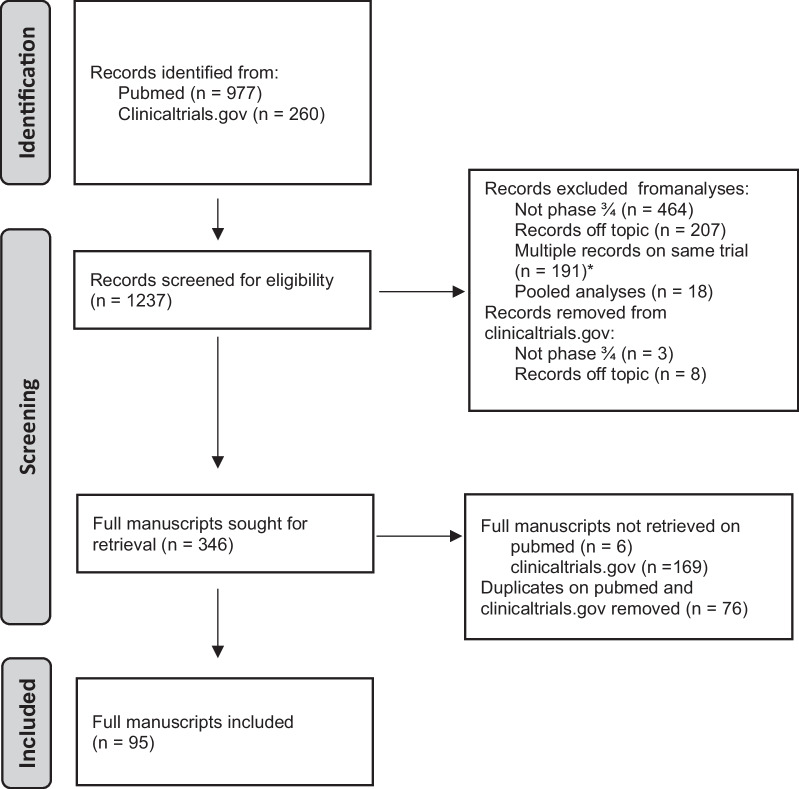
PRISMA diagram of primary search (Phase III and IV trials): Study selection of primary trials of novel breast cancer treatments

We identified 26 drugs within the eight treatment categories. For the primary comprehensive search, 1273 papers were screened and a total of 95 phase III and IV trials were retained, of which 14 are in the neo-adjuvant, 11 in the adjuvant and 70 in the metastatic setting. Figure 2 depicts the PRISMA flow diagram. All original primary manuscripts were published between 12/2006 and 12/2023. All included trials are summarized in Additional file 1: Supplementary Table 2 in the Appendix.

2. Secondary search: phase I and II trials

**Fig. 3:**
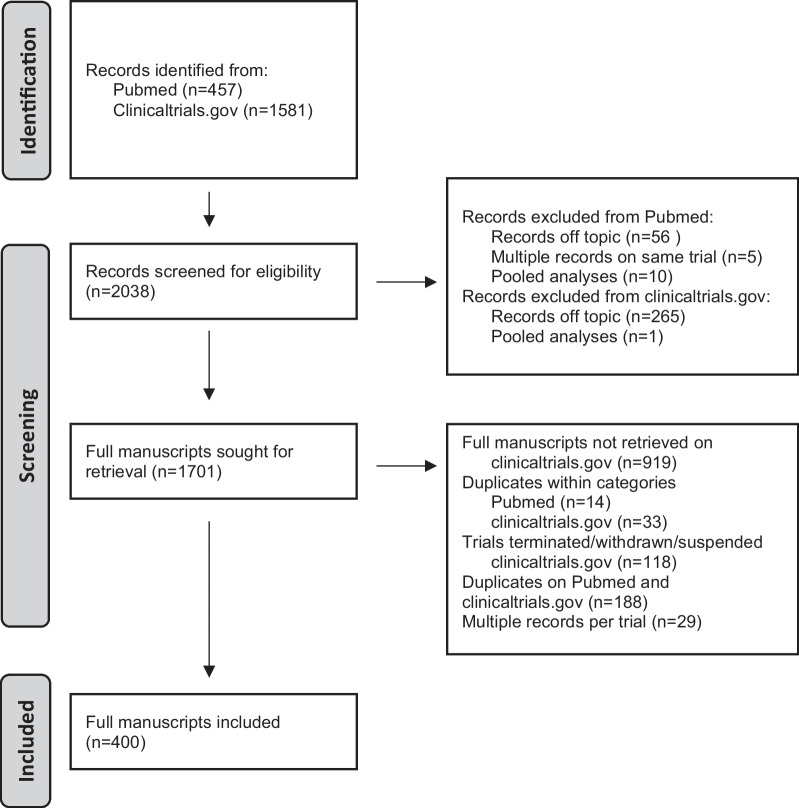
PRISMA diagram of second search (Phase I and II trials)

ClinicalTrials.gov and Pubmed were searched for the phase I and II trials of the 26 drugs identified during the primary search. A total of 2038 papers were screened and a total of 400 manuscripts were retained, of which 318 in metastatic, 5 in adjuvant and 74 in neoadjuvant setting. Three trials were performed in healthy participants [36]. All the included trials are summarized in Additional file 1: Supplemantary Table 3 in the Appendix (Fig. 3).

#### Weight-based vs fixed dose

We assessed the dosing regimen of the drugs under investigation in the clinical trials, as presented in Table 1. Notably, 21 out of 26 drugs (81%) are administered in a fixed dose during phase III/IV trials. Two drugs, atezolizumab and pembrolizumab, have been dosed in a weight-based regimen in some phase I and II trials [37–41], but proceeded to phase III and IV trials in a fixed dose regimen. It is noteworthy that trials employing an ICI in a weight-based regimen did not provide information regarding the weight distribution of the patients. Neither was there a direct comparison between the efficacy of the two regimens for breast cancer specifically. Out of the 26 drugs investigated, 18 were administerd in a fixed dose regimen with no documented exploration of a weight-based approach.

**Table 1:** Overview of the included recent BC drugs recent BC drugs and their dosing regimen and volume of distribution, organised per treatment category

Drug category	Drug	IV/PO	Dosing regimen	
			Phase I–II	Phase III–IV
CDK4/6 inhibitor	Palbociclib	PO	Fixed	Fixed
	Abemaciclib	PO	Fixed	Fixed
	Ribociclib	PO	Fixed	Fixed
	Dalpiciclib	PO	Fixed	Fixed
oSERD	Elacestrant	PO	Fixed	Fixed
ADC	T-DM1	IV	Weight-based	Weight-based
	T-DXd	IV	Weight-based	Weight-based
	Sacituzumab Govitecan	IV	Weight-based	Weight-based
ICI	Atezolizumab	IV	Weight-based	Fixed
			Fixed	
	Pembrolizumab	IV	Weight-based	Fixed
			Fixed	
PARPi	Veliparib	PO	Fixed	Fixed
	Olaparib	PO	Fixed	Fixed
	Talazoparib	PO	Fixed	Fixed
	Iniparib	PO	Weight-based	Weight-based
TKI	Lapatinib	PO	Fixed	Fixed
	Pyrotinib	PO	Fixed	Fixed
	Neratinib	PO	Fixed	Fixed
PI3K/AKT/PTEN	Alpelisib	PO	Fixed	Fixed
	Buparlisib	PO	Fixed	Fixed
	Ipatasertib	PO	Fixed	Fixed
	Taselisib	PO	Fixed	Fixed
	Everolimus	PO	Fixed	Fixed
	Temsirolimus	PO	Fixed	Fixed
	Capivasertib	PO	Fixed	Fixed
Other	Margetuximab	IV	Weight-based	Weight-based
	Tucidinostat	PO	NA	Fixed

*CDK4/6* Cyclin-dependent kinases 4 and 6, *oSERDs* oral selective estrogen receptor degraders, *ADC* antibody drug conjugate, *ICI* immune checkpoint inhibitor, *PARPi* PARP-inhibitor, *TKI* tyrosine kinase inhibitor, *PO* per os, *IV* intravenous

The dosage of sacituzumab-govitecan, trastuzumab emtansine, trastuzumab-deruxtecan, iniparib and margetuximab are based on total body weight and can be adjusted during each administration in case of weight changes over the treatment period. None of the identified drugs were administered by BSA-dosing or lean-based weight dosing.

#### BMI or any other adiposity measurement and diabetes as exclusion criteria

Patients were excluded based on their weight or BMI in 3/495 trials (< 1%). A phase 1 study of ribociclib on healthy subjects excluded those with a BMI > 36 kg/m^2^ or total body weight of > 120 kg [44]. Miller et al., a trial investigating capivaserib in a healthy subjects, also excluded patients with a body weight > 100 kg [45]. Yam et al. on the other hand only included patients with BMI ≥  25 kg/m^2^ to asses the combination of everolimus and metformin [46]. In all further 492 trials (99%), BMI/weight of the patient was never considered an exclusion criterion. Diabetes mellitus type 2 on the other hand, which is the leading comorbidity of obesity [31, 47, 48], was however a criterion for exclusion to some degree in 16/95 of the phase III/IV (17%) trials and 51/400 (13%) of the phase I and II trials. Approximately 51% of the trials that excluded individuals with diabetes to some degree, were studying drugs targeting the PI3K/AKT/PTEN pathway. There is a wide variability in the severity of exclusion based on diabetes in the different trials, details can be retrieve in Additional file 1: Supplementary Table 2 and 3. Certain trials identified uncontrolled or severe diabetes mellitus as an exclusion criterion, without providing further specifics [49–67, 101–106]. Other trials have opted to exclude all individuals with diabetes mellitus [68–74] or patients with diabetic symptoms [75–78] or all patients receiving hypoglycemic treatments [54, 79–81, 106]. Some trials established strict exclusion criteria based on HbA1c and fasting glucose levels [82–100, 107–109, 130]. These clinical trials might not always have adequately enrolled patients with obesity and might therefore not represent the intended general patient population.

#### Reporting of BMI and analysis of treatment efficacy and side-effects according to BMI

BMI or weight of the included patients was reported in 9% of the phase I/II trials (n = 37) and 3% of phase III and IV trials (n = 3), culminating in a total of 8% (n = 40) of all included trials.

The BALLET and TBCRC 043 trials were the only trials performing a subgroup analysis in the original manuscript. The BALLET trial showed that the incidence of adverse events of everolimus was independent of BMI status [110]. In TBCRC 043 it is noted that there was a non-significant trend towards a greater benefit from atezolizumab plus carboplatin in metastatic breast cancer of obese patients (HR, 0.52; *P* = 0.10) [111]. Primary subgroup analyses on differences in therapy efficacy were not performed in any of the other trials. But DeCensi et al. conducted a study on the impact of patients’ weight on the pharmacokinetics of lapatinib, and the findings indicated that weight did not exert a significant impact [112].

For six trials studying CDK4/6 inhibitors, additional retrospective analyses regarding the impact of BMI on treatment efficacy and/or associated side-effects were performed. This was the case for the NEOMONARCH [113], ALTTO [114] and neo-ALTTO trial [115], the PALLAS trial [116] and a pooled analysis of MONARCH 2 and 3 trial [117]. In the NEOMONARCH BMI > 25 kg/m^2^ did not impact change in Ki67% or clinical/radiological response in neoadjuvant setting [113]. Di Cosimo et al. showed that the pathological complete response rate after anti-HER2 therapies in overweight and obese patients with hormone receptor (HR)-positive, HER2-positive BC included in the neo-ALTTO trial was lower compared to their lean counterparts. This was however independent of the treatment arm [115]. The secondary analysis of the ALTTO [114] trials showed that postmenopausal patients with HR-positive HER2-positive BC who were obese at diagnosis and received lapatinib, had a worse prognosis, experienced more grade 3 or 4 adverse events and had higher treatment discontinuation rate [114]. Secondary analyses of the PALLAS trial showed that palbociclib-induced neutropenia was less frequent in overweight and obese patients. A difference in iDFS was not seen [116]. A pooled analysis of Monarch-2 and 3 could not identify any difference in PFS according to BMI [117]. However, the addition of abemaciclib to endocrine therapy resulted in a greater difference in PFS in the normal weight category compared to overweight and obese [117]. Neutropenia was again less frequent in the overweight/obese population compared to normal weight population, suggesting a pharmacokinetic effect.

## Discussion

Several conclusions can be drawn from this systematic review. Firstly, this study emphasizes that most recent drugs are given at fixed doses independently of patient’s weight or BSA in contrast to chemotherapy. Some recent trials question this fixed dose regimen since weight-based dosing mostly results in a lower dose and thus possibly lower cost and less toxicity [118–120]. Defining the maximum tolerated dose (MTD) is a frequent primary objective in phase I/II trials. However, in the case of drugs with a wider therapeutic window, as anticipated in targeted therapies and immunotherapy there is a concern that MTD could result in overdosing. Dose de-escalation might therefore lead to similar therapeutic efficacy with less toxicity [116, 121]. The Optimus Project, an FDA initiative, aims to reform dosing in drug development in early clinical trials [122]. It is hypothesized that the impact of obesity on the pharmacokinetics is less relevant in drugs with a broader therapeutic window, such as targeted therapies and immunotherapies. However, the potential impact on therapeutic efficacy through other alternative mechanisms remains uncertain due to a lack of dedicated studies on this subject.

In 2021, the American Society of Clinical Oncology (ASCO) issued guidelines on the appropriate systematic therapy dosing for obese adult patients with cancer [123]. The guidelines stated that cytotoxic therapy should be offered at full dose for obese patient and therefore dose capping should be avoided. Guidelines regarding targeted therapies and immunotherapy prescribe that these treatments should be used in all patients, regardless of obesity status. However, evidence supporting equal efficacy in obese versus lean patients is low [124]. To be able to guide our clinical practice pharmacokinetic/pharmacodynamic studies as well as dose–response analyses are needed for these drugs.

Further, obesity was in less than 1% of the trials an exclusion criterion. Since diabetes mellitus type 2 is the leading comorbidity of obesity [31], we investigated diabetes mellitus type 2 as an exclusion criterium in the context of our systematic review as a surrogate marker of obesity [125]. A note of caution should however be taken as only about 16–20% of patients with obesity develop diabetes mellitus type 2 [126, 127] and as diabetes type 2 can be diagnosed after inclusion in the study. It is noteworthy that the majority of the trials excluding patients with diabetes involve PIK3CA or mTOR inhibitors, both of which are known to cause hyperglycemia. Consequently, caution is needed when including diabetic patients in such trials. Furthermore, we acknowledge that there are more comorbidities of obesity that we did not consider in this systematic review, such as heart disease, hypertension or obstructive sleep apnea. It is important to highlight that the exclusion of obese patients indirectly contributes to a racial bias in some environments, given that obesity is more prevalent within the black or Hispanic communities in the USA [128]. Race and other socioeconomic characteristics are associated with obesity and therefore should also be considered and documented [129, 130]. Reimbursements of therapies can be based on the inclusion criteria outlined in phase III/IV trials, underscoring the importance of prioritizing inclusivity in these trials.

Thirdly, we can conclude that there is a clear gap in knowledge on the efficacy of novel anti-cancer treatments in different weight categories. Most of the clinical trials do not report individual patient data about BMI and do not perform analyses to identify potential variations in treatment efficacy according to BMI.

It is known that BMI itself is a suboptimal marker for adiposity since it does not reflect body composition and therefore underestimates adiposity in postmenopausal women [131–135]. BSA—based dosing is suboptimal for some obese patients and might lead to underexposure, while weight-based dosing can lead to overexposure [136]. Alternative measurements of adiposity include body composition measurements or measurements of the adipocytes in the breast tissue [137]. However, the precise relationship between these measures, BMI and their potential impact on treatment efficacy is not yet fully understood. Further research to understand these relationships is necessary before these variables can be used in clinical practice to determine treatment options.

Phase I and II trials are designed to study pharmacokinetics and identify the maximum tolerated dose for progression into phase III and IV trials [138]. It is noteworthy that body composition measurements are rarely reported across all phases of clinical trials. A comprehensive study of obesity throughout the entire drug development process is important as its impact extents beyond pharmacokinetics and as phase I and II trials are often conducted in a population limited in size and generally less representative of the affected population [139]. These trials often exclude patients with cardiovascular history, diabetes, hypertension or low performance score, indirectly resulting to the underrepresentation of obese patients within these cohorts [139, 140].

This systematic review has several limitations. Firstly, there is a publication bias. We have only included full manuscripts but several, more recent trials [141, 142], currently only have an abstract or poster presentation available. In addition to this, non-publication of a full manuscript is common among phase I and II trials [143]. We found 74 abstracts of results of phase I and II trials without a full manuscript. Secondly, we suspect that the patient’s weight and height are available in the electronic case report forms of most patients but not reported in publication nor analysed elsewhere.

If we would consistently report on BMI, precaution is still needed if subgroup analyses for therapeutic efficacy according to BMI are performed, as these will be most of the time unplanned analyses with possibly suboptimal statistical power. In the event that poorer survival is observed among individuals classified as obese or overweight, it will be important to distinguish the predictive versus prognostic effect. We strongly encourage investigators to study the impact of obesity on therapeutic efficacy (and the incidence and severity of treatment-related side effects) and possibly use real world data for a representative population.

To our knowledge, no previous systematic review has been conducted to point out this gap in information. Hereby we hope to emphasize the need to take BMI or alternative body composition measurements at time of treatment administration into consideration when evaluating BC therapies.

In conclusion, this systematic review emphasizes the lack of reporting BMI or other adiposity measurements in clinical trials for BC treatment. Incorporating BMI or other adiposity measurements into trial design and analysis can aid in identifying potential differences in treatment efficacy among different weight categories, ultimately resulting in more effective and tailored treatment methods for patients with BC.

### Supplementary Information

**Additional file 1. Appendix: Supplementary Table 1:** Search terms clinical drug trials • **Supplementary Table 2:** Acquired data per included clinical drug trial, phase III and IV • **Supplementary Table 3:** Acquired data per included clinical drug trial, phase I and II.

## Data Availability

All data generated or analysed during this study are included in this published article.
