# Supplementary material for: Reporting on patient’s body mass index (BMI) in recent clinical trials for patients with breast cancer: a systematic review
Source: Breast Cancer Res. 2024 May 22;26:81. doi: 10.1186/s13058-024-01832-7 (PMC11112918; doi:10.1186/s13058-024-01832-7)
Supplement: Supplementary file 1 — Additional file 1. Appendix: Supplementary Table 1: Search terms clinical drug trials • Supplementary Table 2: Acquired data per included clinical drug trial, phase III and IV • Supplementary Table 3: Acquired data per included clinical drug trial, phase I and II. [file 13058_2024_1832_MOESM1_ESM.docx]

**Appendix: Reporting on patient’s body mass index (BMI) in recent clinical trials**

**for patients with breast cancer: a systematic review**

Van Cauwenberge J. , Van Baelen K. *et al.*

Overview:

- Supplementary Table 1a and b: Search terms clinical drug trials
- Supplementary Table 2: Acquired data per included clinical drug trial, phase III and IV
- Supplementary Table 3: Acquired data per included clinical drug trial, phase I and II

**Supplementary Table 1a: Search terms clinical drug phase III and IV trials**

| **Search terms** | | **Clinicaltrials.gov** | **Pubmed** |
| --- | --- | --- | --- |
| **General search terms used** | |  |  |
|  | | i. Condition or disease: Breast Cancer  ii. Study Phase: Phase 3, Phase 4 | "Breast Neoplasms"[Mesh] AND article type: ‘Clinical Trial, phase III’ OR ‘Clinical Trial, phase IV’ |
| **Additional search terms used per drug class** | | |  |
|  | CDK4/6 inhibitors | Other terms: “CDK4/6 inhibitor” OR “palbociclib” OR “abemaciclib” OR “ribociclib ” | "palbociclib" [Supplementary Concept] OR "ribociclib " [Supplementary Concept] OR "abemaciclib" [Supplementary Concept] |
|  | Oral SERDs | Other terms: “SERD” OR “selective estrogen receptor degrader” OR “amcenestrant” OR “elacestrant” OR “imlunestrant” OR “camizestrant” OR “giredestrant” OR “SAR439859” OR “RAD1901” OR “LY3484356” OR “AAZD9833” OR “GDC-9545” | "selective estrogen receptor degrader" [Supplementary Concept] OR "oral selective estrogen receptor degrader" [Supplementary Concept] OR "elacestrant" [Supplementary Concept] OR "amcenestrant" [Supplementary Concept] OR “SAR 439859” [Supplementary Concept] OR "imlunestrant" [Supplementary Concept] OR “LY3484356” [Supplementary Concept] OR "camizestrant" [Supplementary Concept] OR “AZD9833” [Supplementary Concept] OR "giredestrant" [Supplementary Concept] OR “GDC-9545” [Supplementary Concept] |
|  | Antibody drug conjugates | Other terms: “Antibody-drug conjugates” OR “T-DM1” OR “trastuzumab emtansine” OR “T-DXd” OR “trastuzumab deruxtecan” OR “sacituzumab govitecan” | "immunoconjugates" [Mesh] OR "sacituzumab govitecan" [Supplementary Concept] OR "trastuzumab deruxtecan" [Supplementary Concept] OR "Ado-trastuzumab Emtansine” [Mesh] |
|  | Immune checkpoint inhibitors | Other terms: “Immune Checkpoint inhibitor” OR “pembrolizumab” OR “PD-L1 inhibitor” OR “PD1-inhibitor” OR “atezolizumab” OR “nivolumab” OR “ipilimumab” OR “Anti-CTLA-4” | "Immune Checkpoint Inhibitors"[Mesh] OR "pembrolizumab" [Supplementary Concept] OR "atezolizumab" [Supplementary Concept] OR "nivolumab"[Mesh] OR "ipilimumab"[Mesh] OR “Anti-CTLA-4” OR “PDL-1 inhibitor” OR “PD1 inhibitor” |
|  | PARP inhibitors | Other terms: “PARP inhibitors” OR “olaparib” OR “niraparib” OR “talazoparib” OR “veliparib” | "Poly(ADP-ribose) Polymerase Inhibitors"[Mesh] OR "olaparib” [Supplementary Concept] OR "talazoparib" [Supplementary Concept] OR "niraparib” [Supplementary Concept] OR "veliparib" [Supplementary Concept] OR “iniparib” [Supplementary Concept] |
|  | Tyrosine kinase inhibitors | Other terms: “Tyrosine Kinase Inhibitors” OR “lapatinib” OR “neratinib” OR “pyrotinib" OR “tucatinib” | "Tyrosine Kinase Inhibitors"[Mesh] OR "lapatinib"[Mesh] OR "neratinib" [Supplementary Concept] OR "pyrotinib" [Supplementary Concept] OR "tucatinib" [Supplementary Concept] |
|  | PI3K/AKT/PTEN inhibitor | Other terms: “PIK3CA inhibitors” OR “AKTinhibitor” OR “alpelisib” OR “buparlisib” OR “ipatasertib” OR “taselisib” OR “capivasertib” OR “mTOR inhibitors” OR “everolimus” OR “temsirolimus” OR “sirolimus” | "alpelisib" [Supplementary Concept] OR "NVP-BKM120" [Supplementary Concept] OR "ipatasertib" [Supplementary Concept] OR "2-(3-(2-(1-isopropyl-3-methyl-1H-1,2-4-triazol-5-yl)-5,6-dihydrobenzo(f)imidazo(1,2-d)(1,4)oxazepin-9-yl)-1H-pyrazol-1-yl)-2-methylpropanamide" [Supplementary Concept] OR “capivasertib” OR “buparlisib” OR “Taselisib” OR "everolimus"[Mesh] OR "temsirolimus" [Supplementary Concept] OR "sirolimus" [Supplementary Concept] |
|  | Others | Other terms:  “margetuximab” OR “tucidinostat” | "margetuximab" OR "N-(2-amino-5-fluorobenzyl)-4-(N-(pyridine-3-acrylyl)aminomethyl)benzamide" [Supplementary Concept] |

SERD: selective estrogen receptor degrader

**Supplementary Table 1b: Search terms clinical drug phase I and II trials**

| **Search terms** | | **Clinicaltrials.gov** | **Pubmed** |
| --- | --- | --- | --- |
| **General search terms used** | |  |  |
|  | | i. Condition or disease: Breast Cancer  ii. Study Phase: Phase 1, Phase 2 | "Breast Neoplasms"[Mesh] AND article type: ‘Clinical Trial, phase I’ OR ‘Clinical Trial, phase II’ |
| **Additional search terms used per drug class** | | |  |
|  | CDK4/6 inhibitors | Other terms: “palbociclib” OR “abemaciclib” OR “ribociclib ” OR “Dalpiciclib” | "palbociclib" [Supplementary Concept] OR "ribociclib " [Supplementary Concept] OR "abemaciclib" [Supplementary Concept] OR "dalpiciclib" [Supplementary Concept] |
|  | Oral SERDs | Other terms “elacestrant” | ("Breast Neoplasms"[Mesh]) AND "elacestrant" [Supplementary Concept] |
|  | Antibody drug conjugates | Other terms “T-DM1” OR “trastuzumab emtansine” OR “T-DXd” OR “trastuzumab deruxtecan” OR “sacituzumab govitecan” | "immunoconjugates" [Mesh] OR "sacituzumab govitecan" [Supplementary Concept] OR "trastuzumab deruxtecan" [Supplementary Concept] OR "Ado-trastuzumab Emtansine” [Mesh] |
|  | Immune checkpoint inhibitors | Other terms: “pembrolizumab” OR “atezolizumab” | "pembrolizumab" [Supplementary Concept] OR "atezolizumab" [Supplementary Concept] |
|  | PARP inhibitors | Other terms: “olaparib” OR “talazoparib” OR “veliparib” OR “iniparib” | "olaparib” [Supplementary Concept] OR "talazoparib" [Supplementary Concept] OR "veliparib" [Supplementary Concept] OR “iniparib” [Supplementary Concept] |
|  | Tyrosine kinase inhibitors | Other terms: “lapatinib” OR “neratinib” OR “pyrotinib" | "lapatinib"[Mesh] OR "neratinib" [Supplementary Concept] OR "pyrotinib" [Supplementary Concept] |
|  | PI3K/AKT/PTEN inhibitor | Other terms: “alpelisib” OR “buparlisib” OR “ipatasertib” OR “capivasertib” OR “everolimus” OR “temsirolimus” | "alpelisib" [Supplementary Concept] OR "ipatasertib" [Supplementary Concept] OR “capivasertib” OR “buparlisib” OR "everolimus"[Mesh] OR "temsirolimus" [Supplementary Concept] |
|  | Others | Other terms:  “margetuximab” OR “tucidinostat” | "margetuximab" OR "tucidinostat” |

**Supplementary Table 2: Acquired data per included clinical drug trial, phase III and IV**

| **Setting** | **Drug** | **Trial** | **Enrollment** | **Clinical subtype** | **Treatment arms** | **Primary endpoint** | **Secondary endpoints** | **Exclusion based on BMI/ weight /…** | **Exclusion based on diabetes** | **Is number or % per BMI category reported** | **% per BMI category** | **Is number or % of diabetes reported** | **Other adiposity measurements reported** | **Subgroup analyses by BMI category performed** | **Retrospective analyses performed** |
| --- | --- | --- | --- | --- | --- | --- | --- | --- | --- | --- | --- | --- | --- | --- | --- |
| **CDK4/6 inhibitors** | | | | | | | | | | | | | | | |
| Neoadjuvant | palbociclib | SAFIA  doi:10.1007/ S00432-023-04588-3 | 354 | HR+ HER2- | °fulvestrant +/- goserelin + palbociclib  °fulvestrant +/- goserelin + placebo | pCR | °Radiological response  °Rate of BCS  °Safety/ Tolerability  °DFS  °OS | No | Yes, uncontrolled diabetes | No | NA | No | No | NA | No |
| Adjuvant | abemaciclib | MONARCHE doi:10.1016/ j.annonc.2021.09.015 | 5637 | HR+ HER2- | °endocrine treatment (TPC) + abemaciclib  °endocrine treatment (TPC) | iDFS | °iDFS in case of Ki67 ≥20%  °DRFS  °OS  °PK  °FACT-B, FACIT-F, EQ-5D-5L | No | No | No | NA | No | No | NA | No |
|  | palbociclib | PENELOPE-B doi:10.1200/ JCO.20.03639 | 1250 | HR+ HER2- | °endocrine treatment (TPC) + palbociclib  °endocrine treatment (TPC) + placebo | iDFS | °iDFS excluding second non-breast cancers  °DDFS  °OS  °iDFS in luminal B  °Compliance/ Safety  °Patient reported outcomes, QALY  °AUC  °Correlation between exposure/efficacy and/or safety | No | Yes, uncontrolled diabetes | No | NA | No | No | NA | No |
|  | palbociclib | PALLAS doi:10.1200/ JCO.21.02554 | 5796 | HR+ HER2- | °endocrine treatment (TPC) + palbociclib  °endocrine treatment (TPC) | iDFS | °iDFS excluding second non-breast cancers  °DRFS  °OS  °LRRFS | No | No | No | NA | No | No | NA | Yes |
| Metastatic | abemaciclib | MONARCH2  doi:10.1200/ JCO.2017.73.7585 | 669 | HR+ HER2- | °fulvestrant + abemaciclib  °fulvestrant + placebo | PFS | °OS  °ORR  °DOR  °DCR  °CBR  °mBPI-sf  °PK  °EQ-5D-5L, EORTC QLQ-C30, EORTC QLQ-BR23 | No | No | No | NA | No | No | NA | Yes |
|  | abemaciclib | MONARCH3 doi: 10.1038/ s41523-018-0097-z | 493 | HR+ HER2- | °AI + abemaciclib  °AI + placebo | PFS | °OS  °ORR  °DOR  °DCR  °CBR  °ES-5D-5L, EORTC QLQ-C30; EORTC QLQ-BR23  °PK | No | No | No | NA | No | No | NA | Yes |
|  | abemaciclib | MONARCH Plus doi: 10.1177/ 1758835920963925 | 463 | HR+ HER2- | °AI + abemaciclib  °AI + placebo  °fulvestrant + abemaciclib  °fulvestrant + placebo | PFS | °OS  °ORR  °DOR  °DCR  °CBR  °EORTC QLQ-C30  °PK | No | No | No | NA | No | No | NA | No |
|  | palbociclib | PALOMA2 doi:10.1056/ NEJMoa1607303 | 666 | HR+ HER2- | °letrozole + palbociclib  °letrozole + placebo | PFS | °ORR  °DOR  °CBR  °PFS by biomarkers  °QTc  °Ctrough  °EQ-5D, FACT-B  °TEAE  °OS  °Survival probability  °CTCAE | No | No | No | NA | No | No | NA | No |
|  | palbociclib | PALOMA3 doi:10.1016/ S1470-2045(15)00613-0 | 521 | HR+ HER2- | °fulvestrant + palbociclib  °fulvestrant + placebo | PFS | °OS  °ORR  °DOR  °CBR  °Survival probabilities  °Ctrough  °EORTC QLQ-C30, EORTC QLQ-BR23, EQ-5D  °TTD  °TEAE | No | No | No | NA | No | No | NA | No |
|  | palbociclib | PALOMA4 doi:10.1016/ j.ejca.2022.08.012 | 340 | HR+ HER2- | °letrozole + palbociclib  °letrozole + placebo | PFS | °ORR  °DOR  °CBR  °OS  °Survival Probability  °TEAE  °CTCAE  °Ctrough  °EQ-5D, FACT-B  °% positive cells for Ki67  °Detection in ER | No | No | No | NA | No | No | NA | No |
|  | palbociclib | PADA-1 doi:10.1016/ S1470-2045(22)00555-1 | 1017 | HR+ HER2- | °AI + palbociclib  °fulvestrant + palbociclib | °TEAE  °PFS | °Time to strategy failure  °Chemotherapy-free survival  °EORTC QLQ-C30  °Other line of therapy  °OS | No | No | No | NA | No | No | NA | No |
|  | palbociclib | PEARL doi:10.1016/ j.ejca.2022.03.006 | 693 | HR+ HER2- | °fulvestrant/ exemestane + palbociclib  °capecitabine | PFS | °ORR  °CBR  °DOR  °OS  °TEAE | No | No | No | NA | No | No | NA | No |
|  | ribociclib | MONALEESA2 doi:10.1093/ annonc/mdy155 | 668 | HR+ HER2- | °letrozole + ribociclib  °letrozole + placebo | PFS | °ORR  °OS  °CBR  °Time to deterioration ECOG performance status  °Safety and Tolerability  °EORTC QLQ-C30  °QTc | No | No | No | NA | No | No | NA | No |
|  | ribociclib | MONALEESA3 doi:10.1200/ JCO.2018.78.9909 | 726 | HR+ HER2- | °fulvestrant + ribociclib  °fulvestrant + placebo | PFS | °OS  °ORR  °TTD  °ECOG performance status  °Safety and Tolerability  °EORTC QLQ-C30  °CBR  °TTR  °DOR | No | No | No | NA | No | No | NA | No |
|  | ribociclib | MONALEESA7 doi:10.1016/ S1470-2045(18)30292-4 | 672 | HR+ HER2- | °AI/tamoxifen + goserelin + ribociclib  °AI/tamoxifen + goserelin + placebo | PFS | °OS  °ORR  °CBR  °Safety and Tolerability  °TTR  °DOR  °TTD °ECOG performance status  °EORTC QLQ-C30 | No | No | No | NA | No | No | NA | No |
|  | ribociclib | RIBECCA doi:10.1016/ j.ejca.2021.11.025 | 502 | HR+ HER2- | °letrozole + ribociclib in postmenopausal women or in men  °letrozole + goserelin + ribociclib in pre/perimenopausal women  °letrozole +/- goserelin + ribociclib in pretreated women or men | CBR | °PFS  °OS  °ORR  °EORTC QLQ-C30, EORTC QLQ-BR23  °TTD °EORTC Global Health Status  °TEAE | No | No | No | NA | No | No | NA | No |
|  | ribociclib | COMPLEEMENT1 doi:10.1007/ s10549-021-06334-0 | 3246 | HR+ HER2- | °letrozole +/- goserelin/leuprolide + ribociclib | °TEAE  °SEA | °TTP  °ORR  °CBR  °FACT-B | No | No | No | NA | No | No | NA | No |
|  | ribociclib | BioItaLEE doi:10.1016/ j.ejca.2023.03.001 | 287 | HR+ HER2- | °ribociclib + letrozole  °alpelisib + fulvestrant | % of patients with ctDNA alterations | °Change in TK1 concentrations  °Change in TMB  °% of patients with mutations  °% of patients with alterations in liquid biopsy vs. tissue biopsy  °Micro-environment parameters  °TTP °adverse events  °ORR °CBR | No | No | No | NA | No | No | NA | No |
|  | Dalpiciclib | DAWNA-2 doi:10.1016/ S1470-2045(23)00172-9 | 426 |  | °dalpiciclib + anastrozole/letrozole ° placebo + anastrozole/letrozole | °PFS (investigator assessed) | °PFS per recist,  °OS  ° ORR  °DOR  °CBR  °AEs | No | No | No | NA | No | No | NA | No |
| **Oral SERDs** | | | | | | | | | | | | | | | |
| Metastatic | elacestrant | EMERALD III doi:10.1200/ JCO.22.00338 | 477 | HR+ HER2- | °SOC (AI /fulvestrant)  °elacestrant | PFS | °OS  °BICR-assessed PFS and OS  °PFS assessed by the investigator  °ORR  °DOR  °CBR  °Safety and Tolerability | No | No | No | NA | No | No | NA | No |
| **ADCs** | | | | | | | | | | | | | | | |
| Neoadjuvant | T-DM1 | KRISTINE doi:10.1016/ S1470-2045(17)30716-7 | 444 | HER2+ | °trastuzumab + pertuzumab  °T-DM1 + pertuzumab | pCR | °OS  °Rate of BCS  °EFS  °iDFS  °EORTC QLQ-C30, EORTC QLQ-BR23 modified  °Cmax  °Cmin  °Plasma DM1 concentrations, Serum DM1 catabolites concentrations  °TEAE  °ATA | No | No | No | NA | No | No | NA | No |
| Adjuvant | T-DM1 | KAITLIN doi:10.1200/ JCO.21.00896 | 1846 | HER2+ | °AC followed by trastuzumab + pertuzumab + taxane  °AC followed by  T-DM1 + pertuzumab | iDFS | °IDFS + Second Primary Non-Breast Cancer  °DFS  °DRFI  °OS  °TEAE  °% with decrease LVEF  °EORTC QLQ-C30, EORTC QLQ-BR23 | No | Yes, uncontrolled diabetes | No | NA | No | No | NA | No |
|  | T-DM1 | KATHERINE doi:10.1056/ NEJMoa1814017 | 1487 | HER2+ | °trastuzumab  °T-DM1 | iDFS | °IDFS + Second Primary Non-Breast Cancer  °DFS  °OS  °DRFI  °TEAE  °Cardiac dysfunction  °EORTC QLQ-C30, EORTC QLQ-BR23  °Serum concentrations, plasma concentrations of DM1  °T-DM1 Exposure  °ATA | No | No | No | NA | No | No | NA | No |
| Metastatic | T-DM1 | EMILIA doi:10.1016/ S1470-2045(17)30312-1 | 991 | HER2+ | °T-DM1  °lapatinib + capecitabine | °% with PD by IRC  °PFS by IRC  °% death  °OS  °% alive at year 1, % alive at year 2 | °% with PD by Investigator  °PFS by Investigator  °ORR  °DOR  °CBR  °% Treatment Failure  °% Symptom progression  °Time to Symptom progression | No | No | No | NA | No | No | NA | No |
|  | T-DM1 | TH3RESA doi:10.1016/ S1470-2045(17)30313-3 | 602 | HER2+ | °T-DM1  °TPC | °PFS  °OS | °ORR  °DOR  °6m and 1y Survival  °EORTC QLQ-BM22 | No | No | No | NA | No | No | NA | No |
|  | T-DM1 | KAMILLA doi: 10.1016/ j.esmoop.2022.100561 | 2185 | HER2+ | °T-DM1 all participants  °T-DM1 Asian participants | AEPI | °AESI  °PFS  °OS  °ORR  °CBR  °DOR  °TTR  °Number and Type of Hospitals Visits | No | No | No | NA | No | No | NA | No |
|  | T-DM1 | MARIANNE doi:10.1200/ JCO.2016.67.4887 | 1095 | HER2+ | °trastuzumab + taxane  °T-DM1 + pertuzumab  °T-DM1 + pertuzumab placebo | °% Death and PD by IRC  °PFS by IRC | °% Death prior to clinical cut off  °OS  °% Death and PD by Investigator  °PFS by Investigator  °% with Treatment Failure  °TTF  °1y Survival  °Grade 3 TEAE  °% Death at 2y  °OS 2y  °CTCAE  °ECOG Performance status  °Hospitalization days  °% Hospitalizations  °ORR  °CBR  °FACT-TaxS  °FACT-C, HRQOL, FACT-B, RSCL, WPAI  °Outcomes in Low HER2 mRNA levels | No | No | No | NA | No | No | NA | No |
|  | T-DM1 | ELAINA doi:10.21037/ tbcr-23-2 | 200 | HER2+ | °T-DM1 °lapatinib + capecitabine | PFS | °ORR °DOR °OS °Adverse events °Concentration T-DM1 °Concentration DM1 °Concentration trastuzumab °ATA °PRO | No | No | No | NA | No | No | NA | No |
|  | T-DXd | DESTINY-Breast02 doi:10.1016/S0140-6736(23)00725-0 | 608 | HER2+ | °T-DXd  °trastuzumab + capecitabine °lapatinib + capecitabine | PFS by BICR | °OS  °ORR  °DOR  °PFS by Investigator | No | No | No | NA | No | No | NA | No |
|  | T-DXd | DESTINY-Breast03 doi:10.1056/ NEJMoa2115022 | 524 | HER2+ | °T-DXd  °T-DM1 | PFS by BICR | °OS  °ORR  °DOR  °PFS by Investigator | No | No | No | NA | No | No | NA | No |
|  | T-DXd | DESTINY-Breast04 doi:10.1056/ NEJMoa2203690 | 557 | HR+ HER2- TNBC | °T-DXd  °TPC (capecitabine, eribulin, gemcitabine, paclitaxel, nab-paclitaxel) | PFS by BICR | °PFS by Investigator  °OS  °ORR  °DOR | No | No | No | NA | No | No | NA | No |
|  | Sacituzumab-Govitecan | TROPICS02 doi:10.1200/ JCO.22.01002 | 543 | HR+ HER2- | °sacituzumab-govitecan  °TPC (capecitabine, eribulin, gemcitabine, vinorelbine) | PFS | °ORR  °OS  °DOR  °CBR  °EORTC QLQ-C30  °TEAE  °SAE  °Laboratory Abnormalities  °ECOG Performance status | No | No | No | NA | No | No | NA | No |
|  | Sacituzumab-Govitecan | ASCENT doi:10.1056/ NEJMoa2028485 | 529 | TNBC | °sacituzumab-govitecan  °TPC (capecitabine, eribulin, gemcitabine, vinorelbine) | PFS by IRC | °OS  °ORR  °Time to OR  °DOR  °TTP  °CBR  °TEAE  °EORTC QLQ-C30  °Laboratory Abnormalities | No | No | No | NA | No | No | NA | No |
| **Immune Checkpoint Inhibitor** | | | | | | | | | | | | | | | |
| Neoadjuvant | Atezolizumab | Impassion031 doi:10.1016/ S0140-6736(20)31953-X | 333 | TNBC | °chemotherapy + atezolizumab  °chemotherapy + placebo | °pCR  °pCR in PD-L1 positive tumors | °EFS  °EFS in PD-L1 positive tumors  °DFS  °DFS in PD-L1 positive tumors  °OS  °OS in PD-L1 positive tumors  °EORTC QLQ-C30  °TEAE  °Cmin  °Cmax  °ATA | No | No | No | NA | No | No | NA | No |
|  | Atezolizumab | Impassion050 doi:10.1200/ JCO.21.02772 | 454 | HER2+ | °ddAC + paclitaxel/ trastuzumab/ pertuzumab + atezolizumab  °ddAC + paclitaxel/ trastuzumab/ pertuzumab + placebo | °pCR in PD-L1 positive tumors  °pCR in ITT population | °pCR by HR status  °pCR in PD-L1 negative tumors  °EFS  °DFS  °OS  °EORTC QLQ-C30  °TEAE  °Cmin Atezolizumab  °Cmax Atezolizumab  °Ctrough for Pertuzumab and Trastuzumab  °Cmin T-DM1  °Cmax T-DM1  °ATA Atezolizumab, Trastuzumab, Pertuzumab, T-DM1  °pCR based on PIK3CA mutation  °EFS based on PIK3CA mutation  °DFS based on PIK3CA mutation  °OS based on PIK3CA mutation | No | No | No | NA | No | No | NA | No |
|  | Atezolizumab | NeoTRIP doi: 10.1016/ j.annonc.2022.02.004 | 278 | TNBC | °carboplatin/ abraxane + surgery + AC  °carboplatin/ abraxane + atezolizumab + surgery + AC | EFS | °pCR  °COR  °DEFS  °TEAE | No | No | No | NA | No | No | NA | No |
|  | Pembrolizumab | KEYNOTE522 doi:10.1056/ NEJMoa2112651 | 1174 | TNBC | °chemotherapy + pembrolizumab  °chemotherapy + placebo | °pCR  °EFS | °EFS in PD-L1 positive tumors  °OS  °TEAE  °EORTC QLQ-C30, EORTC QLQ-BR23 | No | No | No | NA | No | No | NA | No |
| Metastatic | Atezolizumab | Impassion130 doi:10.1056/ NEJMoa1809615 | 902 | TNBC | °nab-paclitaxel + atezolizumab  °nab-paclitaxel + placebo | °PFS  °PFS in PD-L1 positive tumors  °OS  °OS in PD-L1 positive tumors | °ORR  °ORR in PD-L1 positive tumors  °DOR  °DOR in PD-L1 positive tumors  °EORTC QLQ-C30  °TEAE  °ATA  °Cmax  °Cmin  °Plasma concentrations Paclitaxel | No | Yes, uncontrolled diabetes type 1 | No | NA | No | No | NA | No |
|  | Atezolizumab | Impassion131 doi:10.1016/ j.annonc.2021.05.801 | 651 | TNBC | °paclitaxel + atezolizumab  °paclitaxel + placebo | °PFS in PD-L1 positive tumors  °PFS in ITT population | °OS in PD-L1 positive tumors  °OS in ITT population  °% alive at 12 and 18m  °EORTC QLQ-C30  °% Alive without PD at 12m  °ORR in PD-L1 positive tumors  °ORR  °DOR  °CBR  °Cmin Atezolizumab, Cmin Paclitaxel  °Cmax Atezolizumab, Cmax Paclitaxel  °TEAE  °ATA  °OS by PD-L1 status  °PFS by PD-L1 status  °C-DOR | No | Yes, uncontrolled diabetes type 1 | No | NA | No | No | NA | No |
|  | Pembrolizumab | KEYNOTE119 doi:10.1016/ S1470-2045(20)30754-3 | 622 | TNBC | °pembrolizumab  °chemotherapy (capecitabine, eribulin, gemcitabine, vinorelbine) | °OS in PD-L1 CPS ≥10  °OS in PD-L1 CPS ≥1  °OS | °ORR in PD-L1 CPS ≥10  °ORR in PD-L1 CPS ≥1  °ORR  °PFS in PD-L1 CPS ≥10  °PFS in PD-L1 CPS ≥1  °PFS  °DOR in PD-L1 CPS ≥10  °DOR in PD-L1 CPS ≥1  °DOR  °DCR in PD-L1 CPS ≥10  °DCR in PD-L1 CPS ≥1  °DCR  °TEAE  °Discontinuation due to TEAE | No | No | No | NA | No | No | NA | No |
|  | Pembrolizumab | KEYNOTE355 doi:10.1056/ NEJMoa2202809 | 882 | TNBC | °pembrolizumab + nab-paclitaxel  °pembrolizumab + paclitaxel  °pembrolizumab + gemcitabine/ carboplatin  °pembrolizumab + chemotherapy  °placebo + chemotherapy | °TEAE  °Discontinuation due to TEAE  °PFS  °PFS in PD-L1 CPS ≥1  °PFS in PD-L1 CPS ≥10  °OS  °OS in PD-L1 CPS ≥1  °OS in PD-L1 CPS ≥10 | °ORR in PD-L1 CPS ≥10  °ORR in PD-L1 CPS ≥1  °ORR  °DOR in PD-L1 CPS ≥10  °DOR in PD-L1 CPS ≥1  °DOR  °DCR in PD-L1 CPS ≥10  °DCR in PD-L1 CPS ≥1  °DCR  °TEAE  °Discontinuation due to TEAE  °EORTC QLQ-C30, EORTC QLQ-BR23 | No | No | No | NA | No | No | NA | No |
| **PARP inhibitors** | | | | | | | | | | | | | | | |
| Neoadjuvant | Veliparib | BrighTNess  doi: 10.1016/ S1470-2045(18)30111-6 | 634 | TNBC | °veliparib + carboplatin + paclitaxel followed by AC  °placebo + carboplatin + paclitaxel followed by AC  °placebo + placebo + paclitaxel followed by AC | pCRh | °EFS  °OS  °BCR | No | No | No | NA | No | No | NA | No |
| Adjuvant | Olaparib | OlympiA doi:10.1056/ NEJMoa2105215 | 1836 | HR+ HER2- TNBC | °olaparib  °placebo | iDFS | °DDFS  °OS  °Contralateral Breast Cancers, New Primary Ovarian Cancer, New Primary Fallopian Tube Cancer and New Primary Peritoneal Cancer  °FACIT-F, EORTC QLQ-C30 | No | No | No | NA | No | No | NA | No |
| Metastatic | Iniparib | NCT00938652 doi:10.1200/ JCO.2014.55.2984 | 519 | TNBC | °gemcitabine/ carboplatin  °gemcitabine/ carboplatin + iniparib | °PFS  °OS | °BOR  °ORR | No | No | No | NA | No | No | NA | No |
|  | Olaparib | OlympiAD doi:10.1056/ NEJMoa1706450 | 302 | HR+ HER2- TNBC | °olaparib  °TPC | PFS | °Time to second progression or death  °OS  °ORR  °EORTC QLQ-C30  °PFS by BICR | No | No | No | NA | No | No | NA | No |
|  | Olaparib | LUCY doi:10.1016/ j.ejca.2021.03.029 | 256 | HR+ HER2- TNBC | olaparib | PFS | °OS  °TFST  °TSST  °TDT  °PFS on subsequent therapy  °CRR  °DOR  °TEAE | No | No | No | NA | No | No | NA | No |
|  | Talazoparib | EMBRACA doi:10.1056/ NEJMoa1802905 | 431 | HR+ HER2- TNBC | °talazoparib  °TPC | PFS | °ORR  °OS  °Cthrough  °TEAE  °Grade 3/4 Laboratory Abnormalities  °Changes in vital signs  °Concomitant Medication | No | No | No | NA | No | No | NA | No |
|  | Veliparib | BROCADE3 doi:10.1016/ S1470-2045(20)30447-2 | 509 | HR+ HER2- TNBC | °veliparib + carboplatin + paclitaxel  °placebo + carboplatin + paclitaxel | PFS | °OS  °CBR  °ORR  °PFS on subsequent therapy | No | No | No | NA | No | No | NA | No |
| **Tyrosine kinase inhibitors** | | | | | | | | | | | | | | | |
| Neoadjuvant | Lapatinib | NSABP protocol B-41 doi:10.1016/ S1470-2045(13)70411-X | 529 | HER2+ | °trastuzumab  °lapatinib | pCR | °clinical complete response  °cardiac events  °CTCAE side adverse events | No | No | No | NA | No | No | NA | No |
|  | Lapatinib | GeparQuinto doi:10.1016/ S1470-2045(11)70397-7 | 615 | HER2+ | °epirubicin/ cyclophosphamide/ trastuzumab + docetaxel/ trastuzumab  °epirubicin/ cyclophosphamide/ lapatinib + +docetaxel/lapatinib | pCR | °Toxic effects  °Compliance  °Response rate physical examination  °Response rate imaging  °Breast conversation rate  °pCR according to diff definitions | No | Yes, uncontrolled diabetes or insulin dependent | No | NA | No | No | NA | No |
|  | Lapatinib | ALLIANCE doi:10.1200/ JCO.2015.62.1268 | 305 | HER2+ | °paclitaxel + trastuzumab  °paclitaxel + trastuzumab + lapatinib  °paclitaxel + lapatinib | pCR | °pCR axilla  °adverse events | No | No | No | NA | No | No | NA | No |
|  | Lapatinib | NeoALLTO doi:10.1016/ S0140-6736(11)61847-3 | 455 | HER2+ | °lapatinib  °trastuzumab  °lapatinib + trastuzumab | pCR | °locoregional total pCR  °objective tumor response rate (physical examination)  °Breast conserving rate  °Node negativity at surgery  °Safety and tolerability  °DFS  °OS  °Biomarker expression | No | Yes, uncontrolled diabetes | No | NA | No | No | NA | Yes |
|  | Lapatinib | EPHOS B doi:10.1158/ 1078-0432.CCR-21-3177 | 257 | HER2+ | °no (neo)adjuvant therapy  °trastuzumab  °lapatinib  °lapatinib + trastuzumab | °Increase in apoptosis  °Ki67  °RFS | °Angiogenic serum markers  °Molecular markers  °Time to local recurrence  °Time to distant recurrence  °OS | No | No | No | NA | No | No | NA | No |
|  | Pyrotinib | PHEDRA doi:10.1186/ s12916-022-02708-3 | 355 | HER2+ | °trastuzumab/ docetaxel/pyrotinib  °trastuzumab/ docetaxel/placebo | pCR | °pCR rate peripheral vs central  °Overall response rate  °EFS  °DFS  °DDFS | No | No | No | NA | No | No | NA | No |
| Adjuvant | Lapatinib | TEACH doi:10.1016/ S1470-2045(12)70508-9 | 3161 | HER2+ | °trastuzumab  °lapatinib | DFS | °time to recurrence  °OS  °Time to CNS recurrence  °QoL  °Toxic effects | No | No | No | NA | No | No | NA | No |
|  | Lapatinib | ALTTO doi:10.1200/ JCO.2015.62.1797 | 8381 | HER2+ | °trastuzumab 52w  °lapatinib 52w  °trastuzumab + lapatinib 52w  °trastuzumab 12 -> lapatinib 34w | DFS | °OS  °General Safety  °Cardiac Safety  °Time to recurrence  °Time to distant recurrence  °Time to CNS recurrence | No | No | No | NA | No | No | NA | Yes |
|  | Neratinib | ExteNET doi:10.1016/ j.clbc.2020.09.014 | 2840 | HER2+ | After 1y trastuzumab  °neratinib  °placebo | iDFS | °DFS for DCIS  °Time to distant recurrence  °Distant DFS  °Time to CNS recurrence  °Overall survival  °Safety | No | No | No | NA | No | No | NA | No |
| Metastatic | Lapatinib | NCT00078572 doi:10.1056/ NEJMoa064320 | 408 | HER2+ | °capecitabine  °capecitabine + lapatinib | Time to progression | °PFS  °OS  °clinical benefit rate  °Safety | No | No | No | NA | No | No | NA | No |
|  | Lapatinib | EGF30001 doi:10.1200/ JCO.2008.16.2578 | 579 | HR+ HER2- TNBC | °paclitaxel + placebo  °paclitaxel + lapatinib | Time to progression | °ORR  °CBR  °Time of response  °Event free survival  °OS  °Safety | No | No | No | NA | No | No | NA | No |
|  | Lapatinib | EGF104900 doi:10.1200/ JCO.2011.35.6725 | 291 | HER2+ | °trastuzumab + lapatinib  °lapatinib | PFS | °ORR  °CBR  °QOL  °OS | No | No | No | NA | No | No | NA | No |
|  | Lapatinib | NCT00073528 doi:10.1200/ JCO.2009.23.3734 | 1286 | HR+ HER2- HR+ HER2+ | °letrozole + lapatinib  °letrozole + placebo | PFS | °ORR  °CBR  °OS  °Safety  °PFS | No | No | No | NA | No | No | NA | No |
|  | Lapatinib | NCT00281658 doi:10.1200/  JCO.2011.40.5241 | 444 | HER2+ | °paclitaxel + placebo  °paclitaxel + lapatinib | OS 53m | °OS 190m  °PFS  °ORR  °TTR  °Safety  °CBR  °DOR | No | No | No | NA | No | No | NA | No |
|  | Lapatinib | NCIC CTG MA.31 doi:10.1200/ JCO.2014.56.9590 | 537 | HER2+ | °taxane + lapatinib  °taxane + trastuzumab | PFS | °OS  °ORR  °TTR  °DOR  °CBR  °Adverse Events (AEs)  °QoL | No | No | No | NA | No | No | NA | No |
|  | Lapatinib | CEREBEL doi:10.1200/  JCO.2014.57.1794 | 540 | HER2+ | °capecitabine +trastuzumab  °capecitabine + lapatinib | Number of participants with CNS as first site of relapse | °PFS  °Time to first CNS progression  °Incidence of CNS progression at any time  °OS  °ORR  °DOR  °Safety | No | No | No | NA | No | No | NA | No |
|  | Lapatinib | ALTERNATIVE  doi:10.1200/  JCO.20.01894 | 355 | HR+ HER2+ | °lapatinib + trastuzumab + AI  °trastuzumab + AI  °lapatinib + AI | PFS (radiologic and non- radiologic response) | °OS  °ORR  °CBR  °Duration of response  °QoL | No | No | No | NA | No | No | NA | No |
|  | Lapatinib | DETECT III  doi:10.1007/ s10549-010-1163-x | 254 | HR+ HER2- TNBC | °standard therapy  °standard therapy + lapatinib | PFS | °ORR  °CBR  °OS  °Dynamic of CTC  °QoL  °Safety and Tolerability  °Intensity of Pain  °Level of Compliance | No | No | No | NA | No | No | NA | No |
|  | Lapatinib | NCT00508274 doi:10.5732/ cjc.010.10507 | 52 | HER2+ | °lapatinib + capecitabine | CBR | °PFS  °TTR  °DOR  °CNS as first relapse  °Safety | No | No | No | NA | No | No | NA | No |
|  | Lapatinib | NCT00272987 doi:10.1634/ theoncologist.2012-0129 | 63 | HER2+ | °lapatinib (1000mg) + paclitaxel (80mg/m^2^)  °lapatinib (1000mg) + paclitaxel (70mg/m^2^)  lapatinib (750mg) + paclitaxel (80mg/m^2^) | Safety and tolerability | °CBR °PFS  °TTR  °DOR | No | No | No | NA | No | No | NA | No |
|  | Lapatinib | CALGB 40302 doi:10.1200/ JCO.2014.56.7941 | 295 | HR+ HER2- HR+ HER2+ | °fulvestrant + lapatinib  °fulvestrant + placebo | PFS | °Toxicity  °Objective tumor response  °OS | No | No | No | NA | No | No | NA | No |
|  | Neratinib | NALA doi:10.1200/  JCO.20.00147 | 621 | HER2+ | °capecitabine + neratinib  °capecitabine + lapatinib | °PFS  °OS | °Time to intervention for CNS  °ORR  °Investigator assessed PFS  °CBR  °DOR  °HRQoL  °Safety | No | No | No | NA | No | No | NA | No |
|  | Pyrotinib | PHOEBE doi:10.1016/ S1470-2045(20)30702-6 | 267 | HER2+ | °capecitabine + pyrotinib  °capecitabine + lapatinib | PFS | °Safety  °OS  °ORR  °TTP  °DOR  °CBR | No | No | No | NA | No | No | NA | No |
|  | Pyrotinib | Phenix doi:10.21037/ tbcr-20-25 | 279 | HER2+ | °capecitabine + pyrotinib  °capecitabine + placebo | PFS by IRC | °PFS by investigator °ORR °Disease control rate °CBR °DOR °OS  °Safety | No | No | No | NA | No | No | NA | No |
|  | Pyrotinib | PHILA doi:10.1136/ bmj-2023-076065 | 590 | HER2+ | °pyrotinib + trastuzumab+ docetaxel  °placebo + trastuzumab + docetaxel | °PFS | °AE  °OS  °ORR  °DOR  °CBR | No | No | No | NA | No | No | NA | No |
| **PIK3CA/AKT/PTEN inhibitor** | | | | | | | | | | | | | | | |
| Neoadjuvant | Everolimus | GeparQuinto doi:10.1093/ annonc/mdu455 | 1948 | HR+ HER2- TNBC | °epirubicin/ cyclophosphamide/ trastuzumab + docetaxel/ trastuzumab  °epirubicin/ cyclophosphamide/ lapatinib + +docetaxel/  lapatinib  °paclitaxel  °epirubicin/ cyclophosphamide + docetaxel  °epirubicin/ cyclophosphamide + docetaxel + bevacuzimab  °paclitaxel + everolimus | pCR | °DFS  °LRFS  °LRRFS  °RRFS  °DDFS  °Cerebral DFS  °OS | No | Yes, uncontrolled diabetes and insulin dependent diabetes type 2 | No | NA | No | No | NA | No |
| Adjuvant | Everolimus | NCT01805271 doi:10.1200/ JCO.21.02179 | 1278 | HR+ HER2- | °endocrine therapy + everolimus  °endocrine therapy + placebo | DFS | °OS  °EFS  °Distant metastases - FS  °Second malignancies  °Toxicity | No | Yes, uncontrolled diabetes defined as glycated hemoglobin >7% | No | NA | No | No | NA | No |
|  | Everolimus | MAINtenance Afinitor doi:10.1016/ j.ejca.2021.05.008 | 110 | HR+ HER2- | °endocrine therapy + everolimus  °endocrine therapy + placebo | PFS | °ORR  °OS  °Safety | No | Yes, uncontrolled diabetes defined as fasting serum glucose >1.5xULN | No | NA | No | No | NA | No |
| Metastatic | Alpelisib | SOLAR1 doi:10.1056/ NEJMoa1813904 | 572 | HR+ HER2- | °fulvestrant + alpelisib  °fulvestrant + placebo | PFS | °OS  °Subgroup analyses PIK3CA  °PFS according to ctDNA  °Overall response  °CBR  °Safety | No | Yes, diabetes type 1 or uncontrolled diabetes type 2 defined as fasting plasma glucose level >140mg/dl or glycated hemoglobin >6.4% | No | NA | No | No | NA | No |
|  | Buparlisib | BELLE2 doi:10.1016/ S1470-2045(17)30376-5 | 1147 | HR+ HER2- | °fulvestrant + buparlisib  °fulvestrant + placebo | PFS | °OS  °CBR  °ORR  °PK  °Safety  °QoL | No | No | No | NA | No | No | NA | No |
|  | Buparlisib | BELLE3  doi:10.1016/ S1470-2045(17)30688-5 | 432 | HR+ HER2- | °fulvestrant + buparlisib  °fulvestrant + placebo | PFS by investigator | °OS °PFS by PIK3CA °OS by PIK3CA °ORR by PIK3CA °CBR by PIK3CA °Safety and Tolerability °PK °HRQoL °Time to Definitive Deterioration of ECOG Performance Status | No | No | No | NA | No | No | NA | No |
|  | Buparlisib | BELLE4  doi:10.1093/  annonc/mdw562 | 416 | HR+ HER2- TNBC | °paclitaxel + buparlisib  °paclitaxel + placebo | °PFS °PFS by PIK3CA | °OS °ORR °CBR °PK  °Safety | No | No | No | NA | No | No | NA | No |
|  | Ipatasertib | IPATunity130  doi:10.1007/  s10549-021-06450-x | 222 | HR+ HER2- | °paclitaxel + ipatasertib  °paclitaxel + placebo | PFS | °OS  °ORR  °DOR  °CBR  °Safety  °Patient reported Outcomes | No | Yes, diabetes type 1 or insulin dependent diabetes type 2 | No | NA | No | No | NA | No |
|  | Taselisib | SANDPIPER doi:10.1016/  j.annonc.2020.10.596 | 631 | HR+ HER2- | °fulvestrant + taselisib  °fulvestrant + placebo | PFS by investigator | °OS  °CBR  °Duration of objective response  °BICR-PFS  °TTD  °HRQoL  °Safety | No | No | No | NA | No | No | NA | No |
|  | Capivasertib | Capitello 291  doi:10.1056/  NEJMoa2214131 | 708 | HR+ HER2- | °fulvestrant + capivasertib  °fulvestrant + placebo | PFS by investigator | °OS °ORR °Safety | No | Yes, uncontrolled diabetes defined as glycated hemoglobin >8% or insulin dependent diabetes | No | NA | No | No | NA | No |
| Metastatic | Everolimus | 4EVER doi:10.1002/  ijc.31738 | 281 | HR+ HER2- | °everolimus + exemestane  °exemestane | ORR | °PFS  °ORR  °OS  °Safety  °Resource utilization  °HRQoL | No | No | No | NA | No | No | NA | No |
|  | Everolimus | Everexes doi:10.1007/ s10549-021-06173-z | 235 | HR+ HER2- | everolimus + exemestane | Safety  Tolerability | °ORR  °CBR  °TTD | No | Yes, uncontrolled diabetes defined as fasting serum glucose >1.5xULN | No | NA | No | No | NA | No |
|  | Everolimus | Bolero-1 doi:10.1016/ S1470-2045(15)00051-0 | 719 | HER2+ | °trastuzumab + paclitaxel + everolimus  °trastuzumab + paclitaxel + placebo | PFS | °OS  °objective response rate  °CBR  °Safety | No | No | No | NA | No | No | NA | No |
|  | Everolimus | Bolero-2 doi:10.1007/  s12325-013-0060-1 | 724 | HR+ HER2- | °everolimus + exemestane  °exemestane | PFS | °ORR  °CBR  °OS  °QoL  °Safety  °changes in bone marker levels | No | No | No | NA | No | No | NA | No |
|  | Everolimus | Bolero-3  doi:10.1016/  S1470-2045(14)70138-X | 569 | HER2+ | °trastuzumab + vinolrebine + everolimus  °trastuzumab + vinolrebine + placebo | PFS | °OS  °objective response rate  °CBR  °Safety | No | Yes, uncontrolled diabetes | No | NA | No | No | NA | No |
|  | Everolimus | BALLET doi:10.1093/  annonc/mdw249 | 1151 | HR+ HER2- | °everolimus + exemestane | Safety | °Grade 3-4 AE | No | No | Yes | UW: 37 NW: 836 OW: 698 OB: 391 | No | No | No | Yes |
|  | Everolimus | SWOG 1222 doi:10.1158/  1078-0432.CCR-21-3131 | 37 | HR+ HER2- | °fulvestrant + placebo + placebo  °fulvestrant + everolimus + placebo  °fulvestrant + everolimus + anastrazole | PFS | °OS  °Adverse Events  °CTC analyses | No | Yes, uncontrolled diabetes defined as glycated hemoglobin >7% | No | NA | No | No | NA | No |
|  | Everolimus | INPRES  doi:10.1007/  s11523-018-0596-8 | 44 | HR+ HER2- | °exemestane + everolimus | Everolimus AUC | °Correlation early metabolic response and PFS °Correlation early metabolic response and AUC °Effect dose escalation on metabolic response °Correlation AUC and frequency of adverse events | No | No | No (median per category is reported) | NA | No | No | Yes | No |
|  | Everolimus | IMPROVE doi:10.1186/  s12885-020-06747-y | 77 | HR+ HER2- | °exemestane + everolimus °capecitabine + bevacizumab | Patient preference for type of therapy | °PFS °OS °treatment satisfaction °Qol °Safety | No | No | No (median per category is reported) | NA | No | No | No | No |
|  | Temsirolimus | NCT00083993 doi:10.1200/  JCO.2011.38.3331 | 1112 | HR+ HER2- HR+ HER2+ | °letrozole + placebo  °letrozole + temsirolimus | PFS | °OS  °Tumor response  °Clinical benefit  °TTP  °DOR  °TTF  °Safety  °QoL | No | No | No | NA | No | No | NA | No |
| **OTHER** | | | | | | | | | | | | | | | |
| Metastatic | Tucidinostat | ACE doi:10.1016/ S1470-2045(19)30164-0 | 365 | HR+ HER2- | °tucidinostat + exemestane  °placebo + exemestane | °PFS  °PK Chidamide, PK Exemestane  °Acetylation level histone H3 | °OS  °DOR  °ORR  °CBR | No | No | No | NA | No | No | NA | No |
|  | Margetuximab | SOPHIA  doi:10.1200/  JCO.21.02937 | 536 | HER2+ | °margetuximab + chemotherapy  °trastuzumab + exemestane | °PFS  °OS  °Grade 3 CTCEA | °ORR  °Safety | No | No | No | NA | No | No | NA | No |

AC: anthracycline; AE: adverse events; AEPI: Adverse Events of Primary Interest; AESI: Adverse Events of Special Interest; AI: aromatase inhibition; ATA: anti-therapeutic antibodies; AUC: area under the curve; BCS: breast conserving surgery; BICR: Blinded independent central reviews; BOR: best overall response rate; CBR: clinical benefit rate; C-DOR: duration of confirmed response; Cmax: maximum concentration; Cmin: minimum concentration; CNS: central nervous system; CPS: combined positive score; Ctrough: trough concentration; CRR: clinical response rate; CTC: circulating tumor cell; CTCEA: Common Terminology Criteria for Adverse Events; ctDNA: circulating tumor DNA; DCIS: ductal carcinoma in situ; DCR: disease control rate; ddAC: dose-dense anthracycline; DDFS: distant disease free survival; DEFS: distant event free survival; DFS: disease free survival; DM1: emtansine; DOR: duration of response; DRFI: distant recurrence free interval; DRFS: distant recurrence free survival; EFS: event free survival; ECOG: Eastern Cooperative Oncology Group; EORTC QLQ-BM22: The European Organisation for Research and Treatment of Cancer Quality of Life Questionnaire for patients with bone metastases; EORTC QLQ-BR23: The European Organization for Research and Treatment of Cancer Breast Cancer-Specific Quality of Life Questionnaire; EORTC QLQ-C30: European Organization for Research and Treatment of Cancer Quality of Life Questionnaire Version 3.0; EQ-5D-5L: Euro Quality of Life 5 dimensions 5 level; ER: estrogen receptor; FACIT-F: Functional Assessment of Chronic Illness Therapy – Fatigue; FACT-B: Functional Assessment of Cancer Therapy – Breast; FACT-C: Functional Assessment of Cancer Therapy Colorectal Cancel Module; FACT-TaxS: Functional Assessment of Cancer Therapy Taxane Score; HR: hormone receptor; HRQOL: Health Related Quality of Life; iDFS: invasive disease free survival; IRC: independent review committee; ITT: intention to treat; NA: not applicable; LRFS: local recurrence free survival; LRRFS: locoregional recurrence free survival; LVEF: left ventricular ejection fraction; mBPI-sf: modified Brief Pain Inventory-short form; NW: normal weight; OB: obese; OR: objective response; ORR: overall response rate; OS: overall survival; OW: overweight; pCR: pathological complete response; PD: progressive disease; PFS: progression free survival; PK: pharmacokinetics; QALY: quality adjusted life year; QOL: quality of life; QTc: QT duration corrected for heart rate; RFS: recurrence free survival; RSCL: Rotterdam Symptom Checklist; SAE: serious adverse events; SERD: selective estrogen receptor degrader; SOC: standard of care; T-DM1: trastuzumab-emtansine; TDT: Time to Study Treatment Discontinuation or Death; T-DXd: trastuzumab-deruxtecan; TEAE: treatment emergent adverse event; TFST: time to first subsequent therapy; TMB: tumour mutational burden; TPC: treatment of physician’s choice; TSST: time to second subsequent therapy; TTD: time to deterioration; TTF: time to treatment failure; TTP: time to progression; TTR: time to response; ULN: upper limit of normal; UW: underweight; WPAI: Work Productivity and Activity Impairment

**Supplementary Table 3: Acquired data per included clinical drug trial, phase I and II**

| **Setting** | **Drug** | **Phase** | **Trial** | **Enrollment** | **Clinical subtype** | **Treatment arms** | **Dosing Regimen drug of interest** | **Primary endpoint** | **Secondary endpoints** | **Exclusion based on BMI /weight / …** | **Exclusion based on diabetes** | **Is number or % per BMI category reported** | **% per BMI category** | **Is number or % of diabetes reported** | **Other adiposity measurements reported** | **Subgroup analyses by BMI category performed** | **Pharmacokinetics/Pharmacodynamics reported** |
| --- | --- | --- | --- | --- | --- | --- | --- | --- | --- | --- | --- | --- | --- | --- | --- | --- | --- |
| **CDK 4/6 inhibitors** | | | | | | | | | | | | | | | | | |
| Neoadjuvant | Palbociclib | II | NCT01684215  doi:10.1111/cas.13507 | 42 | ER+ HER2- | Phase I:  °part 1 palbociclib  °part 2 palbocicilib + letrozole  Phase 2: palbociclib + letrozole | Fixed dose | °DLT  °AE | °Safety profile °PK | No | No | No | NA | No | Median body weight | No | Yes, without subgroup analysis according to weight/BMI |
|  |  | II | NA-PHER2  doi: 10.1016/S1470-2045(18)30001-9 | 36 | ER+ HER2+ | °trastuzumab + pertuzumab + palbociclib + fulvestrant | Fixed dose | °Ki67 expression change °apoptosis changes | °ORR  °pCR | No | Yes, uncontrolled diabetes | No | NA | No | No | No | No |
|  |  | II | NeoPal  doi: 10.1093/annonc/mdy448 | 106 | ER+ HER2- | °FEC °AI + Palbo | Fixed dose | °RCB | °Clinical response (US and examination) °CTCAE ° PAM50 score °BCS rate | No | No | No | NA | No | No | No | No |
|  |  | II | NeoPalAna  doi: 10.1158/1078-0432.CCR-16-3206 | 50 | ER+ HER2- | °palbociclib + AI  °AI | Fixed dose | °CCCA | °Clinical response rate °Radiologic Response Rate  °Safety °Change in Ki67 °pCR °OS °PFS | No | No | No | NA | No | No | No | No |
|  |  | Ib/II | CheckMate 7A8  10.1016/j.breast.2023.103580 | 21 | ER+ HER2- | °nivolumab+palbociclib+anastrozole °palbociclib + anastrazole => nivolumab + palbociclib + anastrazole °palbociclib + anastrazole | Fixed dose | °DLT  °RCB | °ORR  °BCS rate  °pCR  °AEs  °Death rate  °discontinuation | No | No | No | NA | No | No | No | No |
|  |  | II | PALLET trial  doi: 10.1200/JCO.18.01624 | 307 | ER+ HER2- | °letrozole  °letrozole => palbociclib + letrozole  °palbociclib => palbociclib + letrozole  °letrozole + palbociclib | Fixed dose | °clinical response °change in Ki67 | °pCR °Changes in surgical intent  °Safety | No | No | No | NA | No | No | No | No |
|  |  | II | PALTAN  doi: 10.1038/s41523-022-00504-z | 26 | ER+ HER2+ | palbociclib + letrozole + trastuzumab +/- gosereline | Fixed dose | °pCR | °Safety  °Tolerability ° PRO | No | No | No | NA | No | No | No | No |
|  |  | II | POP  10.1093/annonc/mdy202 | 100 | HR+ | °Palbocicilib 2w °No intervention | Fixed dose | °KI67<1% °Senescence-associated Β-galactosidase expression increase (but abandoned) | °DLT °relative change in Ki67 | No | No | No | NA | No | No | No | No |
|  | Abemaciclib | II | NeoMONARCH  doi:10.1158/1078-0432 | 224 | HR+ HER2- | °Abemaciclib + ET 2w °Abemaciclib alone °Anastrazole alone | Fixed dose | ki67 change | °pCR °ORR °radiological response °QoL °PK | No | No | No | NA | No | No | No | Yes, without subgroup analysis according to weight/BMI |
|  | Ribociclib | II | CORALLEEN  10.1016/S1470-2045(19)30786-7 | 106 | HR+ HER2- | °ribociclib + letrozole °Doxorubicine +cyclophosphamide+paclitaxel | Fixed dose | Prosigna score | °ORR °pCR °PEPI °RCB °Rate of BC surgery °AEs | No | No | No | NA | No | No | No | No |
|  |  | II | NCT01919229  doi:10.1016/j.breast.2016.06.008 | 14 | HR+ HER2- | °letrozole °letrozole + ribociclib 400mg/d °letrozole + ribociclib 600mg/d | Fixed dose | °Ki67 change | °Safety and Tolerability °ECG °pRB °PK °CDK1 expression | No | No | Yes | Cohort A: BMI 29 kg/m^2^, cohort B 26kg/m^2^, cohort C 29 kg/m^2^ | No | No | No | Yes, without subgroup analysis according to weight/BMI |
| Adjuvant | Palbociclib | II | NCT02040857  doi: 10.1093/annonc/mdz198 | 162 | HR+ HER2- | Palbociclib + AI/Tamoxifen | Fixed dose | °2y treatment discontinuation rate | °2y t disc rate by AI vs Tamoxifen °Toxicity °Fatigue,  °alopecia | No | No | No | NA | No | No | No | No |
| Metastatic | Palbociclib | II | FLIPPER  doi: 10.1016/j.ejca.2021.11.010 | 189 | HR+ HER2- | Fulvestrant + palbociclib Fulvestrant + placebo | Fixed dose | °PFS 1y | °ORR °CBR °OS °PRO and physical function | No | No | No | NA | No | No | No | No |
|  |  | I | NCT01320592  doi: 10.1158/1078-0432.CCR-18-0790 | 27 | All BC | ° Abemaciclib + Trastuzumab + Fulvestrant ° abemaciclib + trastuzumab  ° trastuzumab + SOC | Fixed dose | °AE | °MTD °Biomarkers for safety, efficacy and tolerability | No | All patients with diabetes mellitus | No | NA | No | No | No | No |
|  |  | II | PALOMA-1, TRIO-18  doi: 10.1007/s10549-020-05755-7 | 165 | ER+ HER2- | ° palbociclib + letrozole ° letrozole | Fixed dose | °AEs  °phase 1 DLTs °PFS phase 2 | Phase 1:  °ORR °CBR °PK parameters °QTc Phase 2:  °ORR °DoR °TTP °Pain °CBR °Tissue biomarkers °Genetic analysis °AEs | No | No | No | NA | No | No | No | Mentioned in the objectives on clinicaltrials.gov, though no results published |
|  |  | II | NCT02536742   doi: 10.1016/j.ejca.2021.12.030 | 122 | HR+ HER2- | palbociclib+ fulvestrant | Fixed dose | °PFS and biomarkers | °Safety °Tolerability | No | yes, if HbA1c>7% | No | NA | No | No | No | No |
|  |  | I | NCT02499146  doi: 10.1007/s00280-021-04263-9 | 26 | ER+ HER2- | palbociclib+ letrozole | Fixed dose | °PK | °AEs °PFS °ORR °DCR °letrozole concentration °plasma Rb expression °ki67 °Thymidine kinase | No | No | No | NA | No | Median body weight | No | No |
|  |  | II | NCT03007979  doi: 10.1038/s41523-022-00399-w | 54 | HR+ HER2- | palbociclib+ letrozole/fulvestrant+/- goserelin | Fixed dose | °G3 Neutropenia | °rate of reduction rate of interruption °AEs °PFS °ORR °CBR | No | No | No | NA | No | No | No | No |
|  |  | II | TREND trial  doi: 10.1093/annonc/mdy214 | 115 | ER+ HER2- | palbociclib+ tamoxifen | Fixed dose | °PFS | °AEs °CBR °OS °response rates | No | No | No | NA | No | No | No | No |
|  |  | II | PARSIFAL  10.1001/jamaoncol.2021.4301 | 486 | HR+ HER2- | palbociclib+ AI  palbociclib+ fulvestrant | Fixed dose | °PFS | °AEs °TTP °OS °CBR °ORR | No | No | No | NA | No | No | No | No |
|  |  | II | BioPER  doi: 10.1158/1078-0432.CCR-22-1281 | 33 | HR+ HER2- | palbociclib+ ET | Fixed dose | °Rb loss °CBR | molecular patterns resistance | No | No | No | NA | No | No | No | No |
|  |  | Ia/b | NCT03332797  doi: 10.1158/1078-0432.CCR-23-1796 | 175 | ER+ HER2- | °Palbociclib+ giredestrant +/- LHRH °giredestrant +/- LHRH | Fixed dose | °AE °MTD °clinical parameters | °PK giredenstrant °PK Palbocicilb °CBR °ORR °DoR | No | No | No | NA | No | Median body weight | No | Mentioned in objectives though no results reported |
|  |  | II | coopERA Breast Cancer  doi: 10.1016/S1470-2045(23)00268-1 | 221 | ER+ HER2- | °giredestrant + palbociclib °AI + palbociclin | Fixed dose | °Ki67 change | °ORR °CCCA 2w °CTCAE °Change clinical parameters  °PK giredestrant | No | No | No | NA | No | No | No | For giredestrant mentioned in objectives, no results reported |
|  |  | II | KCSG-BR15-10  doi: 10.1016/S1470-2045(19)30565-0 | 189 | HR+ HER2- | °palbociclib + exemestane + GnRH agonist °capecitabine | Fixed dose | °PFS | °OS  °QoL  °toxicity °ORR °CBR  °biomarkers | No | No | No | NA | No | No | No | No |
|  |  | II | PALINA  doi: doi:10.1016/j.conctc.2018.05.012 | 35 | HR+ HER2- | °palbociclib+ AI/fulvestrant | Fixed dose | °AE (neutropenia) | °Dose delays by neutropenia °dose reductions by neutropenia °CBR | No | No | Yes | BMI over both arms 22 kg/m^2^ | No | No | No | No |
|  |  | II | Shah et al  doi: 10.1016/j.clbc.2022.12.006 | 12 | HR+ HER2+ | °palbociclib+ trastuzumab | Fixed dose | °ORR CNS | °PFS °ORR °time to CNS progression °OS °Safety °Tolerability | No | No | No | NA | No | No | No | No |
|  |  | Ib/II | NCT02448771  doi: 10.1158/1078-0432 | 36 | HR+ HER2- | °palbociclib+ bazedoxifen | Fixed dose | °CBR  °CBR by ESR1 °Neutrophil decrease | °ORR °PFS °OS  °PFS by ESR1 °OS by ESR1 °ORR by ESR1 | No | No | No | NA | No | No | No | No |
|  |  | I/Ib | Haley et al  doi: 10.1016/j.clbc.2021.03.005 | 18 | HER2+ | °palbociclib+ TDM1 | Palbociclib: Fixed dose  T-DM1: Weight based | °DLT | °PK  °Biomarkers °RR °DoR °PFS | No | No | No | NA | No | No | No | Mentioned in objectives though no results reported |
|  |  | II | PATRICIA 2  doi: 10.1158/1078-0432.CCR-20-0844 | 71 | HR+ HER2+ | °palbociclib+ trastuzumab +/- letrozole | Fixed dose | °PFS 6m | °Toxicity, PFS °ORR °PFS and ORR according to PAM50 | No | No | No | NA | No | No | No | No |
|  | ribociclib | II | MAINTAIN  doi: 10.1200/JCO.22.02392 |  | HR+ HER2- | °ribociclib + fulvestrant/exemestane °fulvestrant/exemestane + placebo | Fixed dose | °PFS24w | °ORR °CBR  °AE °ctDNA detection | No | No | No | NA | No | No | No | No |
|  |  | I/II | TRINITI-1  doi: 10.1158/1078-0432.CCR-20-2114 | 104 | HR+ HER2- | Phase 1:  °ribociclib 200 + everolimus + exemestane °ribociclib 250 + everolimus + exemestane °ribociclib 300 + everolimus + exemestane Phase II:  °ribociclib 300 + everolimus + exemestane °ribociclib 200mg + everolimus +exemestane | Fixed dose | °CBR | °Safety °Biomarkers °PFS °OS °ORR | No | Yes, exclusion of all patients with diabetes mellitus | No | NA | No | No | No | Yes, without subgroup analysis according to weight/BMI |
|  |  | Ib | NCT01857193  doi: 10.1158/1078-0432.CCR-20-1068 | 116 | HR+ HER2- | °ribociclib + everolimus + exemestane | Fixed dose | °DLT °RP2D | °Safety °ORR °DCR °DOR °OS °PFS °PK | No | No | No | NA | No | No | No | Yes, without subgroup analysis according to weight/BMI |
|  |  | Ib/II | NCT02657343  doi: 10.1016/j.clbc.2019.05.010 | 13 | HER2+ | °ribociclib + trastuzumab | Fixed dose | °RP2D °CBR 24w | °safety °ORR °PFS °biomarkers  °PK | No | No | No | NA | No | No | No | Mentioned in objectives though no results reported |
|  |  | II | NCT02187783  doi: 10.1200/PO.18.00383 | 106 (7 BC) | All solid | °ribociclib + trastuzumab + fulvestrant °ribociclib + trastuzumab  °trastuzumab + SOC | Fixed dose | °CBR °ORR | °PFS °OS °DoR | No | No | No | NA | No | No | No | No |
|  |  | II | AMICA  doi: 10.1016/j.breast.2023.08.007 | 53 | HR+ HER2- | °ribociclib + ET  °ET | Fixed dose | °PFS | °OS °CBR °Qol | No | No | Yes | BMI per arm: arm A) 25.7 kg/m^2 ,^ arm B) 27.9 kg/m^2^ | No | No | No |  |
|  |  | Ib | NCT02088684  doi: 10.1158/1078-0432.CCR-20-0645 | 70 | HR+ HER2- | °ribociclib + fulvestrant °ribociclib continuos + fulvestrant °ribociclib + alpelisib + fulvestrant °ribociclib + buparlisib + fulvestrant | Fixed dose | °DLTs °PFS | °Safety and Tolerability °PK °ORR °DoR °OS | No | No | No | NA | No | No | No |  |
|  |  | Ib | MONALEESASIA  doi: 10.1111/cas.14554 | 87 | HR+ HER2- | °ribociclib + letrozole °ribociclib + Tamoxifrn  °ribociclib + I42 | Fixed dose | Phase Ib °dose esc:  DLT Aes °dose exp:  AE | °PK °Dose esc:  Aes °Dose exp:  ORR CBR PFS OS DCR DoR | No | No | Yes | Yes Arm A) 22,1 kg/m^2^, arm B) 22,3 kg/m^2^ arm C) 21,9 kg/m^2^ Arm D) 23,7 kg/m^2^ | No | No | No |  |
|  | abemaciclib | Ib | NCT02057133  doi: 10.3389/fonc.2021.810023 | 67 | HR+ HER2- | Part A-D abemaciclib+ Letrozole abemaciclib+ anastrazole abemaciclib+ Tamoxifen abemaciclib+ Exemestane  abemaciclib+ Exemestane | Fixed dose | °AE | °PK °ORR °PFS °MDASI | No | No | No | NA | No | No | No | Yes, without subgroup analysis according to weight/BMI |
|  |  | II | NCT03703466  doi: 10.1007/s10549-022-06690-5 | 72 | HR+ HER2- | abemaciclib + meal abemaciclib without meal abemaciclib whenever | Fixed dose | °AE  °Diarrhea induced reduction rate °Diarrhea induced interruption rate °use of antidiarrheals | °PK | No | No | yes | Arm A) BMI26,6 kg/m^2^ Arm B) 28,3 kg/m^2^ Arm C) 26 kg/m^2^ | No | No | No | Yes, without subgroup analysis according to weight/BMI |
|  |  | II | MONARCH 1  doi: 10.1158/1078-0432.CCR-17-0754 | 132 | HR+ HER2- | abemaciclib | Fixed dose | °ORR investigator | °CBR °PFS °OS °DoR °DCR °Pain score °PK °Global health score | No | No | No | NA | No | No | No | Mentioned in objectives though no results reported |
|  |  | II | NCT02308020  doi: 10.1158/1078-0432.CCR-20-1764 | 104 | HR+ | abemaciclib +/- ET +/- trastuzumab | Fixed dose | °ORR intracranial | °intra/extracranial ORR °intra/extracranial DCR °intra/extracranial CBR  °PFS °OS | No | No | No | NA | No | No | No | Yes, without subgroup analysis according to weight/BMI |
|  |  | II | nextMONARCH  doi: 10.1007/s10549-022-06662-9 | 234 | HR+ HER2- | abemaciclib + tamoxifen abema | Fixed dose | °PFS | °ORR °DoR °OS °PK °QoL °Pain | No | No | No | NA | No | No | No | Mentioned in objectives though no results reported |
|  |  | II | MOnarcHER  doi: 10.1016/S1470-2045(20)30112-1 | 237 | HR+ HER2+ | ° abemaciclib + Trastuzumab + fulvestrant ° abemaciclib + trastuzumab  ° trastuzumab + SOC | Fixed dose | °PFS | °OS °ORR °DoR °DCR °Pain  °QoL °CBR °EQ-5D-5L Index Score °EQ-5D-5L VAS  °PK | No | No | No | NA | No | No | No | Mentioned in objectives though no results reported |
|  |  | II | ELAINE 2  10.1200/JCO.2022.40.16_suppl.1022 | 29 | HR+ HER2- | °lasofoxifene + abemaciclib | Fixed dose | °Safety  °Tolerability | °PFS °CBR °ORR °DoR PK | No | No | No | NA | No | No | No | Mentioned in objectives though no results reported |
|  |  | II | WJOG11418B NEWFLAME  doi: 10.1136/jitc-2023-007126 | 17 | HR+ HER2- | °nivolumab + abemaciclib | Fixed dose | °safety °cohort: DLT efficacy cohort: ORR | °toxicity °DCR °PFS °OS | No | No | No | NA | No | No | No | No |
|  | Dalpiciclib | Ib | LORDSHIPS   doi: 10.3389/fonc.2022.775081 | 15 | HR+ HER2+ | °pyrotinib + letrozole + dalpiciclib | Fixed dose | °Safety and tolerability °DLTs °MTD °RP2D | °PFS °ORR °DCR °CBR °DoR | No | No | No | NA | No | No | No | No |
|  |  | II | Yan et al  doi: 10.1038/s41591-021-01562-9 | 41 | HER2+ | °pyrotinib + dalpiciclib | Fixed dose | °ORR | °PFS °OS °Safety | No | Yes, severe diabetes | No | NA | No | No | No | No |
| Healthy | palbociclib | I | NCT02085538  doi: 10.1007/s00280-020-04163-4 | 31 | NA | °palbociclib | Fixed dose | °impact of renal impairment | °safety and tolerability in healthy versus subjects with renal impairment | No | No | Yes | Cohort A) 29.2 m/kg^2^, B) 29.9 kg/m^2^, C) 29.8 kg/m^2^ | No | No | No | Yes, without subgroup analysis according to weight/BMI |
|  | ribociclib | I | CLEE011A2117 and CLEE011A2103  doi: 10.1002/cpdd.853 | 38 | NA | °Bio availability:  -ribociclib 600mg po cycle 1, ribociclib IV cycle 2 -ribociclib IV 150mg cycle 1, ribociclib po cycle 2 ° Bio equivalence:  ribociclib in capsule ribociclib in tablet | Fixed dose | °PK | °Safety and tolerability | No | Unclear | Yes | yes, bioavailibity study: median BMI 24,3 kg/m^2^ bioequivalence study:25,78 kg/m^2^ | No | No | No | Yes, without subgroup analysis according to weight/BMI |
|  |  | I | NCT02388620  doi: 10.1002/jcph.1825 | 30 | NA | Ribocicilib | Fixed dose | °PK | °AE | Yes, included only  BMI 18-36 kg/m^2^, and at least 50kg an no more than 120kg | Unclear | Yes | yes, cohort A (normal liver) 30,1 kg/m^2^, cohort B) 30,4 kg/m^2^ cohort C) 29,8 kg/m^2^ cohort D) 31,8 kg/m^2^ | No | No | No | Yes, without subgroup analysis according to weight/BMI |
| **Oral SERDs** | | | | | | | | | | | | | | | | | |
| Metastatic | elacestrant | I | NCT02338349  doi: 10.1200/JCO.20.02272 | 57 | ER+ HER2- | Elacestrant tablets/capsules | Fixed dose | °DLTs | °Safety  °PK  °Tumor endpoints | No | No | No | NA | No | No | No | Yes, without subgroup analysis according to weight/BMI |
| **ADCs** | | | | | | | | | | | | | | | | | |
| Neoadjuvant | T-DM1 | II | TEAL  doi: 10.1186/s13058-019-1186-0 | 30 | HER2+ | °T-DM1 q3w + lapatinib + Nab-paclitaxel  °Trastuzumab q1w + pertuzumab q3w +paclitaxel q1w | T-DM1: weight based dosing Lapatinib: fixed dose | °pCR | °Breast imaging response | No | No | No | NA | No | No | No | No |
|  |  | II | Harbeck et al  doi: 10.1200/JCO.2016.71.9815 | 375 | HR+ HER2+ | °T-DM1 °T-DM1+ ET °trastuzumab + ET | Weight-based dosing | °pCR | °EFS °OS °Safety | No | No | No | NA | No | No | No | No |
|  |  | II | NCT02568839  doi: 10.1001/jamaoncol.2021.1932 | 202 | HER2+ | °docetaxel+trastuzumab+pertuzumab °T-DM1 | Weight-based dosing | °pCR | °clinical and radiological ORR °PFS °iDFS °dDFS °OS °safety °QoL °Functional and biological characteristics °BCS rate | No | No | No | NA | No | No | No | No |
|  |  | II | I-SPY-2  doi: 10.1038/s41467-021-26019-y | 52 | HER2+ | °paclitaxel + trastuzumab °pertuzumab + T-DM1 °trastuzumab+pertuzumab+paclitaxel | Weight-based dosing | °pCR | °RCB °PFS °AE °MRI volume | No | No | No | NA | No | No | No | No |
|  | Sacituzumab-Govitecan | II | NeoSTAR  doi: 10.1038/s41467-021-26019-y | 50 | TNBC | °Sacituzumab govitecan | Weight-based dosing | °pCR | °ORR °Safety °EFS °biomarkers | No | No | No | NA | No | No | No | No |
| Adjuvant | T-DM1 | II | NCT01196052  oi: 10.1200/JCO.2014.58.7782 | 153 | HER2+ | (neo)adj chemotherapy + T-DM1 | Weight-based dosing | °Cardiac events °LVEF | °AE °Completion °PCR °DFS | No | No | No | NA | No | No | No | No |
|  |  | II | ATEMPT  doi: 10.1200/JCO.20.03398 | 497 | HER2+ | °TDM-1 °trastuzumab-paclitaxel | Weight-based dosing | °Clinically relevant toxicities °3y iDFS | °G3-4 incidence °QoL °Neuropathy °Alopecia °effect on work productivity and activity °AE °OS | No | No | No | NA | No | No | No | No |
| Metastatic | T-DM1 | I | NCT03153163  doi: 10.1097/MD.0000000000022886 | 11 | HER2+ | °T-DM1 | Weight-based dosing | °PK | °AEs | No | No | No | NA | No | No | No | Yes, without subgroup analysis according to weight/BMI |
|  |  | Ib | NCT01983501  doi: 10.1001/jamaoncol.2018.1812 | 57 | HER2+ | °tucatinib + T-DM1 | Weight-based dosing | °AE | °dose reduction °ORR °DoR °DCR °CBR °PFS | No | No | No | NA | No | No | No | Yes, without subgroup analysis according to weight/BMI |
|  |  | II | NCT00679341  doi: 10.1200/JCO.2012.44.9694 | 137 | HER2+ | °Trastuzumab + docetaxel °T-DM1 | Weight-based dosing | °PFS | °OS °ORR °DoR °CBR °QoL °PK | No | No | No | NA | No | No | No | Mentioned in objectives though no results reported |
|  |  | II | NCT00509769  doi: 10.1200/JCO.2010.29.5865 | 112 | HER2+ | °T-DM1 | Weight-based dosing | °ORR | °DoR °PFS investigator °PFS independent reviewer | No | No | No | NA | No | No | No | No |
|  |  | II | CirCe T-DM1 trial  doi: 10.1186/s13058-019-1215-z |  | HER2- primary | °T-DM1 | Weight-based dosing | °ORR | °HER2+ CTC °reproducibility HER2 FISH on CTC °PFS °DCR °OS °CTC quantity °AE | No | No | No | NA | No | No | No | No |
|  |  | II | NCT00932373  doi: 10.1200/JCO.2009.26.2071 |  | HER2+ | °T-DM1 | weight based dosing | °AE °DLT °MTD °PK | °ORR °DoR °PFS °anti=therapeutic antibodies | No | No | No | NA | No | No | No | Yes, without subgroup analysis according to weight/BMI |
|  |  | I, II | NCT01702558  doi: 10.1001/jamaoncol.2020.1796 |  | HER2+ | Phase I:  °Capecitabin 750/700/650 + T-DM1 Phase II:  °Capacitabin in MTD + T-DM1 °T-DM1 alone | weight based dosing | Phase 1:  °MTD °DLT  Phase II:  °ORR | Phase I:  °PK Phase II:  °TTR °DoR °Treatment failure °PFS °CBR °death °OS | No | No | No | NA | No | No | No | Yes, without subgroup analysis according to weight/BMI |
|  |  | Iia | NCT00875979  doi: 10.1200/JCO.2013.52.6590 | 64 | HER2+ | °T-DM1 + pertuzumab | Weight-based dosing | °ORR | °DOR °PFS | No | No | No | NA | No | No | No | No |
|  |  | Ib/IIa | NCT00951665  doi: 10.1186/s13058-016-0691-7 | 110 | HER2+ | Phase 1:  ° TDM-1 q3w + paclitaxel ° T-DM1 q3w + paclitaxel + pertuzumab q3w ° T-DM1 q1w + paclitaxel  ° T-DM1 q1w + paclitaxel + Pertuzumab q3w Phase 2:  °Paclitaxel + TDM1 °Paclitaxel + pertuzumab + TDM1 | Weight-based dosing | °AE °DLT °MTD  °Dose modification °Completion rate °PK °Cardiac function | °ORR °DoR °CBR °PFS | No | No | No | NA | No | No | No | Yes, without subgroup analysis according to weight/BMI |
|  |  | Ib/IIa | NCT00934856  doi: 10.1093/annonc/mdw157 | 25 | HER2+ | Phase Ib:  °Docetaxel + T-DM1 different dosing regimens  Phase II:  °Docetaxel + T-DM1  °Docetaxel + T-DM1 + Pertuzumab | Weight-based dosing | °DLT °AE | °PFS °Treatment failure  °TTR  °CBR °DoR °PCR °Anti-therapeutic antibody  °PK | No | No | No | NA | No | No | No | Yes, without subgroup analysis according to weight/BMI |
|  |  | I | NCT04419181  doi: 10.1200/PO.22.00237 | 11 | HER2+ | °T-DM1 | Weight-based dosing | °Platelet function °thrombokinetic changes | °Cause of death  °CBR °Death °AE °LV ejection fraction °ORR | No | No | No | NA | No | No | No | No |
|  |  | II | SAFE-HEaRt  doi: 10.1007/s10549-019-05191-2 | 30 | HER2+ | °trastuzumab +/- pertuzumab °T-DM1 | Weight-based dosing | °Completion rate without Cardiac Event | °Time to develop Cardiac Event °Changes in LVEF °Myocardial Strain With Cardiac Events and Asymptomatic Worsening of Cardiac Function °Trop I and Trop T | No | No | Yes | yes, 16% of patients were lean, 32% of patients were overweight, 52% of patients were obese | Yes, 5% | No | No | No |
|  |  | II | NCT01835236  doi: 10.1001/jamaoncol.2023.2909 | 210 | HER2+ | °pertuzumab + trastuzumab => T-DM1 °pertuzumab + trastuzumab + chemotherapy => T-DM1 | Weight-based dosing | °OS ITT | °PFS °PFS second-line treatment (+/- CNS lesion) °TFS °OS °ORR °DC °ORR second line  °DC second-line °AE °QoL | No | No | No | NA | No | No | No | No |
|  |  | Ib | Thelma  doi: 10.3390/cancers12123509 | 15 | HER2+ | °T-DM1 + doxorubicine | Weight-based dosing | °MTD | °ORR °BOR °CBR °PFS °AE °Ventricular dysfunction  °HER-2 levels °cardiotoxicity °PK | No | No | No | NA | No | No | No | Yes, without subgroup analysis according to weight/BMI |
|  |  | I | NCT03190967  doi: 10.2217/fon-2020-0094 | 12 | HER2+ | °T-DM1 | Weight-based dosing | °Safety and tolerability °RP2D | °time to new parenchymal brain metastases development, °time to whole brain radiation, °OS | No | No | No | NA | No | No | No | Yes, without subgroup analysis according to weight/BMI |
|  |  | Ib | Kojima et al  doi: 10.1159/000497276 | 13 | HER2+ | ° T-DM1 + S-1 (oral 5-FU) | Weight-based dosing | °DLT | °safety °ORR | No | No | No | NA | No | No | No | Yes, without subgroup analysis according to weight/BMI |
|  |  | Ib | JapicCTI-101234  doi: 10.1007/s12282-018-0887-z | 6 | HER2+ | °T-DM1 + pertuzumab | unclear | °Safety °PK | °ORR by RECIST | No | No | No | NA | No | No | No | No |
|  |  | I | Jain et al   doi: 10.1007/s10549-018-4792-0 | 17 | HER2+ | ° T-DM1 + alpelisib | T-DM1: weight based dosing Alpelisib: fixed dose | °MTD °Safety | °ORR | No | No | No | NA | No | No | No | No |
|  |  | II | Watanabe et al  doi: 10.21873/invivo.11088 | 232 | HER2+ | °T-DM1 | Weight-based dosing | °Safety | °AEs | No | No | No | NA | No | yes: mean body weight per cohort | No | No |
|  |  | I | Li et al   doi: 10.1007/s00280-017-3440-4 | 28 | HER2+ | °T-DM1 | Weight-based dosing | °PK | °OFS  °PFS | No | No | No | NA | No | No | No | Yes, without subgroup analysis according to weight/BMI |
|  |  | II | JO22997 t  doi: 10.1093/jjco/hyw013 | 73 | HER2+ | °T-DM1 | Weight-based dosing | °ORR | °PFS °OS °Safety °PK | No | No | No | NA | No | No | No | Yes, without subgroup analysis according to weight/BMI |
|  |  | I | Yamamoto et al  doi: 10.1093/jjco/hyu160 | 10 | HER2+ | °T-DM1 | Weight-based dosing | °MTD | °toxicity  °PK | No | No | No | NA | No | No | No | Yes, without subgroup analysis according to weight/BMI |
|  | T-DXd | II | TUXEDO-1  doi: 10.1038/s41591-022-01935-8 | 15 | HER2+ | T-DXd | weight based dosing | °Response rate of BrainM | PFS OS Safety | No | No | No | NA | No | No | No | No |
|  |  | II | DEBBRAH  doi: 10.1093/neuonc/noac144 | 21 | HER2+ or HER2 low | T-DXd | weight based dosing | °PFS 16w °ORR-intracranial °OS | °ORR °CBR °TTR °DoR °DCR °OS °PFS 6m,14m °safety and tolerability °the best percentage of change from baseline in the size of tumor lesions as per RANO-BM for intracranial lesions and per RECIST v.1.1 for extracranial and overall measurable lesions | No | No | No | NA | No | No | No | No |
|  |  | II | DAISY  doi: 10.1038/s41591-023-02478-2 | 186 | All BC | T-DXd | Weight-based dosing | °ORR | °DOR °OS °PFS °CBR °safety | No | No | No | NA | No | No | No | No |
|  |  | I | NCT03366428  doi: 10.1002/cpt.2757 | 51 | HER2+ or low | T-DXd | Weight-based dosing | °QTc °PK | °ORR °AE | No | No | No | NA | No | No | No | No |
|  |  | II | DESTINY-Breast01  doi: 10.1056/NEJMoa1914510 | 184 | HER2+ | T-Dxd | Weight-based dosing | °ORR | °DCR °CBR °DoR °PFS °Safety | No | No | No | NA | No | No | No | Yes, without subgroup analysis according to weight/BMI |
|  |  | I | NCT02564900  doi: 10.1016/S1470-2045(19)30097-X | 24 total, 17 BC | All BC | T-Dxd | Weight-based dosing | °Safety °MTD °RP2D | Dose esc:  ° DCR ° best OR ° PK ° AEs  Dose expansion:  °DCR °Best OR °DoR °TTR °PFS °OS °Aes | No | No | yes | Dose esc: Cohort A) 24,8 kg/m^2^; B) 22,7 kg/m^2^; C) 20,5 kg/m^2^; D) 20,3 kg/m^2^;E) 21,1 kg/m^2^; F) 21,3 kg/m^2^ | No | No | No | Yes, without subgroup analysis according to weight/BMI |
|  | sacituzumab govitecan | IIb | NCT04454437  doi: 10.1002/ijc.34424 | 107 | TNBC | Sacituzumab govitecan | Weight-based dosing | °ORR | °BOR °CBR °PFS °OS °ORR | No | No | No | NA | No | No | No | No |
|  |  | I/II | NCT01631552  doi: 10.1056/NEJMoa1814213 | 44 | TNBC | Sacituzumab govitecan | Weight-based dosing | °Safety °ORR | °DOR °time to repsonse °CBR °OS °PFS °PK | No | No | No | NA | No | No | No | Mentioned in objectives though no results reported |
| **Immune Checkpoint Inhibitor** | | | | | | | | | | | | | | | | | |
| Neoadjuvant | pembrolizumab | II | NeoImmunoboost, AGO-B-041  doi: 10.1016/j.ejca.2023.01.001 | 50 | TNBC | °nab Paclitaxel + EC + pembrolizumab | fixed dose | °PCR | °safety °QoL | No | No | No | NA | No | overall median body weight | No | No |
|  |  | Ib | KEYNOTE 173  doi: 10.1016/j.annonc.2020.01.072 | 60 | TNC | °pembrolizumab + chemotherapy | fixed dose | °safety °RP2D | °pCR °ORR °EFS °OS | No | No | No | NA | No | No | No | No |
|  |  | II | NEOPACT  10.1001/jamaoncol.2023.5033 | 115 | TNBC | °carboplatin + docetaxel + pembrolizumab | fixed dose | °pCR | °MRD °RFS | No | No | No | NA | No | No | No | No |
|  |  | Ib/II | PANACEA  doi: 10.1016/S1470-2045(18)30812-X | 58 | TNBC | °pembrolizumab + trastuzumab | fixed dose | phase Ib °incidence DLT phase 2:  °ORR | °PDL1 neg ORR °Safety °Tolerability °disease control °DoR °TTP °PFS °OS | No | No | No | NA | No | No | No | No |
|  |  | II | cTRAK TN  doi: 10.1016/j.annonc.2022.11.005 | 280 | TNBC | after ctDNA detection, start pembrolizumab vs observation | fixed dose | °proportion of ctDNA + after 12m and 24m  °absence of detectable ctDNA 6m after pembrolizumab start | °time to ctDNA detection  °detection of M+ at ctDNA +  °lead time between ctDNA detect and disease recurrence  °Absence of detect dtDNA after 6m in observation   °safety and tolerability   °commencement of treatment of those randomised to pembrolizumab | No | No | No | NA | No | No | No | No |
|  |  | II | I-SPY2  doi:10.1001/jamaoncol.2019.6650 | 250 | HER2- | °pembrolizumab + paclitaxel => doxorubicin + cyclophosphamide + pembrolizumab | fixed dose | °pCR | °RCB  °DFS 3y  °DRFS 3y | No | No | No | NA | No | No | No | No |
|  | atezolizumab | II | NCT02530489  doi: 10.1007/s10549-023-06929-9 | 37 | TNBC | °nabpaclitaxel + atezolizumab | Fixed dose | °pCR  °RCB | °PFS °OS | No | uncontrolled diabetes mellitus | No | NA | No | No | No | No |
|  |  | I, II | NCT03881878  doi: 10.1001/jamaoncol.2022.2310 | 67 | HER2+ | °pertuzumab/trastuzumab/atezolizumab/docetaxel | Fixed dose | °pCR | °clinical obj RR °EFS 3y °DFS °OS °toxicity °QoL | No | No | No | NA | No | No | No | No |
|  |  | II | NCT02883062  doi:10.1038/s41523-022-00500-3 | 67 | TNBC | °carboplatin + paclitaxel  °carboplatin+ paclitaxel + atezolizumab | Fixed dose | °TIL °PCR | °Safety biomarkers of response °immuno markers °OS °DFS | No | No | No | NA | No | No | No | No |
| Metastatic | pembrolizumab | II | NCT02648477(a) doi: 10.1007/s00262-023-03470-y | 10 | TNBC | °pembrolizumab + doxorubicin | fixed dose | °ORR | °CBR °PFS °OS °safety | No | No | No | NA | No | No | No | No |
|  |  | II | NCT02648477(b) doi: 10.3390/cancers14174279 | 20 | HR+ HER2- | °pembrolizumab + AI | fixed dose | °ORR | °CBR °PFS °OS °Safety and °tolerability | No | No | No | NA | No | No | No | No |
|  |  | II | PEMBRACA  doi: 10.1016/j.esmoop.2023.101207 | 22 | HER2- | °pembrolizumab + carboplatin | fixed dose | °ORR | °DCR °TTP °DOR °OS | No | No | No | NA | No | No | No | No |
|  |  | Ib | NCT03032107  doi: 10.1136/jitc-2022-005119. | 20 | HER2+ | °TDM1 + pembrolizumab | fixed dose | °safety  °tolerability | °ORR °PFS | No | Yes if uncontrolled diabetes | No | NA | No | No | No | No |
|  |  | II | NCT02768701   doi: 10.1136/jitc-2021-003427 | 40 | TNBC | °1x cyclophosphamide => pembrolizumab | fixed dose | °PFS  °change in Tregs | °ORR °DOR  °OS  °AES | No | No | No | NA | No | No | No | No |
|  |  | I/II | NCT02778685  doi: 10.1016/j.ejca.2021.05.035 | 23 | HR + HER2- | °palbociclib + pembrolizumab +letrozole | fixed dose | °ORR °CRR °PBMC changes | °safety  °tolerability °PFS °CRR °DOR °ctDNA changes | No | No | No | NA | No | No | No | No |
|  |  | II | KELLY  doi: 10.1016/j.ejca.2021.02.028 | 44 | HR+ HER2- | °eribulin + pembrolizumab | fixed dose | °CBR | °ORR  °Duration of Clinical benefit  °DOR°Duration of Clinical benefit  °DOR  °TTR  °PFS  °OS | No | No | No | NA | No | No | No | No |
|  |  | Ib/II | ENHANCE 1  doi: 10.1158/1078-0432.CCR-20-4726 | 167 | TNBC | °eribulin + pembrolizumab | fixed dose | phase 1b: °safety  °tolerability  phase II:  °ORR | °PFS °OS °DoR °CBR | No | No | No | NA | No | No | No | No |
|  |  | II | NCT02971761  doi: 10.1002/onco.13583 | 18 | TNBC, AR+ | °enobosarm + pembrolizumab | fixed dose | °safety °response rate | °PFS  °OS  °toxicity | No | No | No | NA | No | No | No | No |
|  |  | II | NCT03051659   doi: 10.1001/jamaoncol.2020.3524 | 88 | HR+ HER2- | °eribulin + pembrolizumab | fixed dose | °PFS | °ORR °OS | No | No | No | NA | No | No | No | No |
|  |  | II | NCT02886585  doi: 10.1038/s41591-020-0918-0 | 20 | Not only BC | °pembrolizumab | fixed dose | °OS | °toxicity response rate °Time to progression | No | No | No | NA | No | No | No | No |
|  |  | II | NCT03366844  doi: 10.1016/j.clbc.2020.01.012 | 8 | HR+ HER2- | °pembrolizumab + radiotherapy | fixed dose | °ORR | °PFS °OS °Safety | No | No | No | NA | No | No | No | No |
|  |  | II | NCT03044730  doi: 10.1136/jitc-2019-000173 | 30 | °TNBC  °HR+ HER2- | °pembrolizumab +capecitabine | fixed dose | °PFS | °ORR °Safety °tolerability | No | No | No | NA | No | No | No | No |
|  |  | II | NCT02730130  doi: 10.1002/cncr.32599 | 17 | TNBC | °pembrolizumab + radiotherapy | fixed dose | °ORR | °Safety °PFS | No | No | No | NA | No | No | No | No |
|  |  | II | Keynote 086  doi: 10.1093/annonc/mdy517 | 254 | TNBC | °pembrolizumab | fixed dose | cohort A: °ORR °discontinuation rate  °safety  Cohort B:  °Safety | Cohort A)  °ORR °DOR °DCR °PFS °OS  Cohort B)  °ORR  °DCR °DoR °PFS OS °PDL1-ORR | No | No | No | NA | No | No | No | No |
|  |  | II | NCT02054806  doi: 10.1200/JCO.2017.74.5471 | 25 | TNBC | °pembrolizumab | weight-based dosing | °ORR | °Safety °Discontinuation rate °PFS °OS °DoR | No | No | No | NA | No | No | No | No |
|  |  | Ib | PembroPlus  doi: 10.1038/bjc.2017.145 | 49 | TNBC | °pembrolizumab + chemotherapy | weight-based dosing | °R2D | °safety °response °OS °PFS | No | No | No | NA | No | No | No | No |
|  |  | Ib | Keynote -012  doi: 10.1200/JCO.2015.64.8931 | 111 | ER+ | °pembrolizumab | weight-based dosing | °ORR  °Safety | °PFS  °DoR  °OS | No | No | No | NA | No | No | No | No |
|  |  | II | NCT02395627  doi: 10.1038/s41467-020-17414-y | 34 | ER+ | °pembrolizumab + vorinostat + tamoxifen | fixed dose | °ORR  °safety °tolerability | °DoR  °PDL1 expression | No | No | No | NA | No | No | No | Yes, without subgroup analysis according to weight/BMI |
|  |  | I/II | Keynote-162/TOPACIO  doi:10.1001/jamaoncol.2019.1029 | 55 | TNBC | °pembrolizumab + niraparib | P: Fixed dose Niraparib: Fixed dose | °DLTs °ORR | °AEs °ORR iRECIST °DOR iRECIST °DCR °PFS °OS °Pharmacokinetics Niraparib | No | No | No | NA | No | No | No | for niraparib mentioned in objectives though no results reported |
|  |  | II | PANGEA  doi: 10.3390/cancers13215432 | 36 | HER2- | °pembrolizumab + gemcitabine | fixed dose | °DLT °RPD °ORR | °PFS °CBR °CBR 24w °RD °OS °AE °ORR °PFS °CBR °RD | No | No | No | NA | No | No | No | No |
|  |  | II | CHEMOIMMUNE doi: 10.2147/BCTT.S400055 | 20 | HER2- | °pembrolizumab + oral cyclophosphamide | fixed dose | °Toxicity °CBR24w | °ORR °DOR  °PFS °OS °AE reporting | No | No | No | NA | No | No | No | No |
|  |  | I/II | KEYNOTE 037  doi: 10.1200/JCO.2018.78.9602 | 62 | Not only BC | °pembrolizumab + epacadostat | fixed dose | °AE °ORR | °DOR °PFS °Duration of DC °OS °Response score °AE | No | No | No | NA | No | No | No | No |
|  |  | II | INSPIRE  doi: 10.1186/s40425-019-0541-0 | 106 | Not only BC | °pembrolizumab | fixed dose | °Changes in genomic and immune biomarkers pre/on treatment and at progression | °ORR °ctDNA genomic markers  °radiomic imaging °PMs °tumor genomic profile and radiomic signatures  °Changes in immune cel subsets  °PPV and NPV of in vitro predictive assay for pembrolizumab response °Vd °baseline tumor RNA expression °profile  Comparison of baseline RNA experssion for immun inhibitory genes with clinical outcome | No | No | No | NA | No | No | No | Mentioned in objectives though no results reported |
|  |  | Ib | NCT02779751  doi: 10.1038/s41523-022-00482-2 | 54 | HR+ HER2- | ° abemaciclib + pembrolizumab+ anastrazole °abemaciclib + pembrolizumab | fixed dose | ° serious and non-serious AEs | °ORR °DCR °DoR °PFS °OS °PK | No | No | No | NA | No | No | No | Yes, without subgroup analysis according to weight/BMI |
|  |  | I | NCT02646748   doi: 10.1158/2767-9764.CRC-22-0461 | 159 | Not only BC | °pembrolizumab + itacitinib °pembrolizumab + INCB050465 | fixed dose | °safety | °ORR °TILS | No | No | No | NA | No | No | No | Yes, without subgroup analysis according to weight/BMI |
|  |  | I | NCT02872025  doi: 10.1038/s41523-021-00267-z | 9 | DCIS | °pembrolizumab 2x °pembrolizumab 4x °pembrolizumab 2x + mRNA 2752 2-4x °mRNA 2752 2-4x | intratumoral fixed dose | °MTD °DLTs °increase in CD8 Tcells | °Tumor volume °immunological markers | No | No | yes | 6 patients lean, 2 overweight, 1 obese | No | No | No | No |
|  | atezolizumab | II | GELATO  doi: 10.1038/s43018-023-00542-x | 23 | All BC subtypes (ILC) | °atezolizumab + carboplatin | Fixed dose | °PFS 6m | °PFS 12m °nr of patients free at 6m in the IR profile subgroup °nr of patients free at 6m in the non-IR profile subgroup °ORR °AE °OS | No | No | No | NA | No | No | No | No |
|  |  | II | KATE2  doi: 10.1016/S1470-2045(20)30465-4 | 330 | HER2+ | °atezolizumab + TDM1  °placebo + TDM1 | Atezolizumab: Fixed dose  T-DM1: Weight based | °PFS investigator assessed | °OR °DoR  °PK | No | No | No | NA | No | No | No | Mentioned in objectives though no results reported |
|  |  | IIb | ALICE  doi: 10.1038/s41591-022-02126-1 | 70 | TNBC | °Peg liposo doxorubicin + cyclophosphamide+ placebo °Peg liposo doxorubicin + cyclophosphamide+ atezolizumab | Fixed dose | °Toxicity °PFS | °Obj tumor response °OS °DoR °DRR °fatigue 5PRO- °NRS pain PRO °QoL PRO °tumor and °immunol markers °CBR °PDL1 expression | No | No | No | NA | No | No | No | No |
|  |  | II | COLET  doi: 10.1016/j.annonc.2021.01.065 | 147 | TNBC | °Cohort I: cobimetinib + paclitaxel °Cohort II: paclitaxel + placebo °Experimental: cobimetinib + paclitaxel + atezolizumab °Cohort III: cobimetinib + atezolizumab + nab-paclitaxel | Fixed dose | °PFS  °OR | °OS °Cohort I: OR °Cohort I-III DoR (+/- RECIST) °Cohort II-III: PFS RECIST Aes PK | No | No | No | NA | No | No | No | Mentioned in objectives though no results reported |
|  |  | II | TBCRC 043  doi: 10.1001/jamaoncol.2023.5424 | 130 | TNBC | °carboplatin + /-atezolizumab | Fixed dose | °PFS | ORR CBR DOR OS | No | No | Yes | °overweight= 29 pts [27.6%  °Obese:obesity 30 [34.2%]  °Lean 40 [38.1%] | No | No | There was a trend toward greater benefit from the combination for patients with obesity (HR, 0.52; P = .10) and patients with uncontrolled blood glucose levels at prediabetic (HR, 0.62; P = .13) and diabetic (HR, 0.35; P = .09) levels | No |
|  |  | Ib | NCT02605915  doi.org/10.1016/j.clbc.2021.04.011 | 73 | HER2+ | °1A: atezolizumab/Trastuzumab/Pertuzumab °1B: atezolizumab/TDM1 3,6mg °1C: atezolizumab/TDM1 3mg °1D: atezolizumab/TDM1 2,4mg  °1E: atezolizumab/doxorubicin/cyclophosphamide °1F: atezolizumab/Trastuzumab/Pertuzumab/Docetaxel °2A: atezolizumab/trastuzumab/pertuzumab °2B: atezolizumab/TDM1 °2C: atezolizumab/TDM1 expansion °2D: atezolizumab/TDM1 expansion | Atezolizumab: Fixed dose  T-DM1: Weight based | °DLT °AE | °PK  °Dose intensity | No | yes, uncontrolled diabetes | No | NA | No | No | No | Mentioned in objectives though no results reported |
|  |  | I | NCT03853707  doi: 10.1093/oncolo/oyad026 | 28 | TNBC | °Arm A (ipatasertib, carboplatin, paclitaxel) °Arm B (ipatasertib and carboplatin) °Arm C (ipatasertib, capecitabine, atezolizumab) | Fixed dose | °PFS | °OR °OS °CBR | No | No | No | NA | No | No | No | No |
|  |  | Ib | NCT03256344  doi: 10.1016/j.esmoop.2023.100884 | 11 | TNBC | °alimogene Laherparepvec + atezolizumab | Fixed dose | °DLT | °ORR °BOR °DOR °DRR °Lesion level °response in in-and uninjected tumor lesion °DCR °PFS °OS | No | No | No | NA | No | No | No | No |
|  |  | Ib | NCT01633970  doi: 10.1001/jamaoncol.2018.5152 | 33 | TNBC | °atezolizumab + Nab-paclitaxel | Fixed dose | °safety °tolerability | °ORR °ORR °DoR °DCR °PFS °OS °biomarker analysis | No | No | No | NA | No | No | No | No |
|  |  | I | NCT01375842  doi: 10.1038/nature14011 | 227 | TNBC | °atezolizumab | Weight-based dosing and Fixed dose | °safety °tolerability °RP2D | °PK °ORR °DoR °PFS °OS | No | No | No | NA | No | No | No | Yes, without subgroup analysis according to weight/BMI |
| PARP inhibitors | | | | | | | | | | | | | | | | | |
| Neoadjuvant | Olaparib | I | RadioPARP  doi: 10.1002/ijc.33737 | 24 | TNBC | Olaparib + radiotherapy | Fixed dose | °Safety °tolerability | °OS °EFS | No | No | No | NA | No | No | No | No |
|  |  | I | Bundred et al  doi: 10.1007/s10637-012-9922-7 | 60 | All BC | Olaparib | Fixed dose | °RP2D | °confirm exposure in plasma and tissue °PK °safety | No | No | yes | cohort A) 26.9 kg/m^2^ B)29.5 kg/m C)24.1 kg/m^2^ D)26.6 kg/m^2^ E)28.5 kg/m^2^ | No | No | No | Yes, without subgroup analysis according to weight/BMI |
|  |  | II | I-SPY-2  doi: 10.1016/j.ccell.2021.05.009 | 73 | All BC | °Durvalumab + olaparib + paclitaxel °Paclitaxel | Fixed dose | °pCR | °RCB °PFS °AE °MRI volume | No | No | No | No | No | No | No | No |
|  |  | II | PETREMAC  doi: 10.1016/j.annonc.2020.11.009 | 32 |  | °Olaparib => carboplatin + olaparib °Olaparib => Olaparib | Fixed dose | °Predictive and prognostic value of mutations | °epigenetic changes °ORR °Ki67 reduction °EFS °OS °Completion rate °BCsurgery vs ME °AE | No | No | No | NA | No | No | No | No |
|  |  | II | GEPAROLA  doi: 10.1016/j.annonc.2020.10.471 | 107 | HER2- | °Paclitaxel + Olaparib => EC °Paclitaxel + Carboplatin => EC | Fixed dose | °pCR | °Bcsurgery vs ME °ORR °Safety °tolerability | No | No | No | NA | No | No | No | No |
|  | Iniparib | II | SOLTI NeoPARP  doi: 10.1007/s10549-015-3616-8 | 141 | TNBC | °iniparib + paclitaxel  °paclitaxel | Weight-based dosing | °pCR breast | °pCR axilla °best overall response °Bcsurgery rate °safety | No | No | No | NA | No | No | No | No |
|  |  | II | NCT00813956  doi: 10.1200/JCO.2014.57.0085 | 80 | TNBC | °gemcitabine + carboplatin + iniparib | Weight-based dosing | °pCR | °RCB °Safety °radiographic response by MRI °conversion rate to Bcsurgery  °gene expression and treatment response | No | No | No | NA | No | No | No | No |
|  | Talazoparib | II | NCT03499353  doi: 10.1200/JCO.19.01304 | 61 | TNBC | Talazoparib | Fixed dose | °pCR | °Toxicity | No | No | No | NA | No | No | No | No |
|  | Veliparib | II | NCT01042379  doi: 10.1007/s12609-019-00334-2 | 72 | HER2- | veliparib + carboplatin + paclitaxel | Fixed dose | °PCR | °EFS °OS | No | No | No | NA | No | No | No | No |
| Adjuvant | Olaparib | II | OLTRE  doi: 10.1007/s12609-019-00334-2 | 27 | all BC | Olaparib | Fixed dose | changes induced by olaparib on biomarkers (Ki67, TILs, PD-L1, immune cells) | °ORR by BRCA status  °changes in pathologic, radiometabolic, immune markers by BRCA status °tumor mutation analyses °correlation of baseline mutations, gene and protein expression profile with PET/CT and/or clinical response after olaparib °Safety and tolerability °HRQoL | No | No | No | NA | No | No | No | No |
| Metastatic | Olaparib | II | ICEBERG 1  doi: 10.3389/fonc.2020.00954 | 54 | all BC | °olaparib | Fixed dose | °ORR | °DOR °CBR PFS Best percentage change tumor size TEAEs | No | No | No | NA | No | No | No | No |
|  |  | I/II | MEDIOLA  doi: 10.1016/S1470-2045(20)30324-7 | 34 | not only BC | °olaparib => olaparib + durvalumab °olaparib + durvalumab °olaparib + durvalumab + bevacuzimab | Fixed dose | °DCR 12w °Safety and °tolerability | °DCR28w °ORR °DOR °PFS °% change in tumour size 12w, 28w °Best % change in tumour size °Time to study treatment discontinuation or death °OS | No | No | No | NA | No | No | No | No |
|  |  | I | NCT00707707  doi: 10.1186/bcr3484 | 19 | TNBC | °olaparib + paclitaxel | Fixed dose | °Safety and tolerability | °PFS °ORR | No | No | No | NA | No | No | No | No |
|  |  | II | NCT00679783  doi: 10.1016/S1470-2045(11)70214-5 | 91 | not only BC | °olaparib | Fixed dose | °ORR | °DCR °% change in tumour size °PFS | No | No | No | NA | No | No | No | No |
|  |  | I | NCT02484404  doi: 10.1016/j.jtho.2019.04.026 | 9 | not only BC | °durvalumab + olaparib + cediranib | Fixed dose | °RP2D °MTD °DLT | °RR °PK | No | No | No | NA | No | No | No | Yes, without subgroup analysis according to weight/BMI |
|  |  | II | TBCR 048  doi: 10.1200/JCO.20.02151 | 54 | All BC | °olaparib | Fixed dose | °ORR | °CBR °PFS °genomic analyses °AE | No | No | No | NA | No | No | No | No |
|  |  | Ib | NCT01623349  doi: 10.1158/1078-0432.CCR-21-3045 | 17 | TNBC | °olaparib + alpelisib | Fixed dose | °RP2D °MTD °DLT | °Safety °ORR | No | No | No | NA | No | No | No | No |
|  |  | I | NCT02418624  doi10.1002/ijc.33498 | 24 | not only BC | °olaparib + carboplatin | Fixed dose | °MTD | °systemic exposure of olaparib °PK/PD °ORR | No | No | No | NA | No | No | No | Yes, without subgroup analysis according to weight/BMI |
|  |  | II | POLA  doi: 10.3390/cancers14040915 | 73 | not only BC | ° lurbinectedin + olaparib | Fixed dose | °ORR | °PFS °OS °Translational | No | No | No | NA | No | No | No | No |
|  |  | I/Ib | NCT01237067  doi: 10.1158/1078-0432 | 77 | not only BC | °olaparib + carboplatin => olaparib maintenance | Fixed dose | °PK, PD °platinum-DNA adducts | °DNA damage  °PAR concentrations °Safety °ORR | No | No | No | NA | No | Total body weight of population | No | The authors used a model that calculated cofounders and weight did not influence clearance |
|  |  | II | DORA  doi: 10.1158/1078-0432.CCR-23-2513 | 45 | TNBC | °olaparib + durvalumab °olaparib | Fixed dose | °PFS | °OS °CBR °Safety and °tolerability °ORR | No | No | No | NA | No | No | No | No |
|  |  | I | NCT03544125  doi: 10.1038/s41698-021-00232-w | 3 | TNBC | °olaparib + durvalumab | Fixed dose | °feasibility of completing a suite of Clinical Laboratory Improvement Amendments (CLIA) assays | °Safety and °tolerability °ORR °TTP °OS | No | No | No | NA | No | No | No | No |
|  |  | I | NCT00516724  doi: 10.1007/s10637-019-00857-6 | 132 | not only BC | °olaparib (capsule vs tablet) + carboplatin + paclitaxel | Fixed dose | °MTD °Safety and tolerability | °PK °ORR | No | No | No | NA | No | No | No | Yes, without subgroup analysis according to weight/BMI |
|  |  | I/Ib | NCT01445418  doi: 10.1093/jnci/dju089 | 45 | not only BC | °olaparib + carboplatin => olaparib maintenance | Fixed dose | °Safety | °PARP and H2AX activity in mononuclear cells  °ORR | No | No | No | NA | No | No | No | No |
|  |  | Ib | NCT02208375  doi: [10.1093/annonc/mdx367.025](https://doi.org/10.1093/annonc/mdx367.025) | 38 | not only BC | °olaparib + capivasertib °olaparib + vistusertib intermittent °olaparib + vistusertib continuos | Fixed dose | °MTD | °DLT °ORR °PFS °DoR °PK | No | No | No | NA | No | No | No | Mentioned in objectives though no results reported |
|  |  | I/II | UMIN00009498  doi: 10.1016/j.ejca.2018.11.014 | 48 | TNBC | °olaparib + eribulin | Fixed dose | phase 1: °RP2D Phase 2: °Safety  °ORR | phase 1:  °PK Phase 2:  °Best change in tumor size response and BRCA status °PFS °OS | No | Yes,  Patients with diabetes under poor control or who were receiving insulin therapy | No | NA | No | No | Yes, BSA | Yes, without subgroup analysis according to weight/BMI |
|  |  | I | SOLACE  doi: 10.1038/s41416-018-0349-6 | 32 | Not only BC | °olaparib +oCyclophosphamide | Fixed dose | °RP2D °MTD °DLT | Safety and tolerability | No | No | No | NA | No | No | No | Yes, without subgroup analysis according to weight/BMI |
|  |  | I | NCT02093351  doi: 10.1007/s12325-018-0804-z | 97 | Not only BC | °olaparib + tamoxifen/anastrazole/letrozole | Fixed dose | °PK | °safety | No | No | No | NA | No | No | Median body weight | Yes, without subgroup analysis according to weight/BMI |
|  |  | I | Matulonis et al  doi: 10.1093/annonc/mdw672 | 70 | Not only BC | °olaparib + BKM120 | Fixed dose | °MTD | °Toxicity °PK | No | No | No | NA | No | No | No | No |
|  |  | I | van der Noll et al  doi: 10.1007/s10637-019-00856-7 | 21 | Not only BC | °olaparib | Fixed dose | °Safety °Response | °ORR | No | No | No | NA | No | No | No | No |
|  |  | II | NCT01078662  doi: 10.1016/j.ygyno.2015.12.020 | 298 | Not only BC | °olaparib | Fixed dose | °tumour response rate | °ORR  °PFS °OS °DoR °DCR | No | No | No | NA | No | No | No | No |
|  |  | I | NCT00819221  doi: 10.1038/bjc.2014.345 | 44 | Not only BC | °olaparob + liposomal doxorubicin | Fixed dose | °RP2D | °Safety °PK PD °ORR | No | No | No | NA | No | No | No | Yes, without subgroup analysis according to weight/BMI |
|  |  | I | NCT00782574  doi: [10.1093/annonc/mdu187](https://doi.org/10.1093/annonc/mdu187) | 54 | Not only BC | °olaparib + cisplatin | Fixed dose | °Safety and tolerability | °PK °ORR °AE | No | No | No | NA | No | No | No | Yes, without subgroup analysis according to weight/BMI |
|  |  | I | NCT01116648  doi: 10.1016/S1470-2045(14)70391-2 | 28 | Not only BC | °olaparib + cediranib | Fixed dose | °DLT °MTD | °Toxicity profile °ORR °CBR °PFS | No | No | No | NA | No | No | No | No |
|  |  | I | NCT00710268  doi: 10.1038/bjc.2011 | 12 | Not only BC | °olaparib + bevacuzimab | Fixed dose | °Safety and tolerability °RP2D | °PK | No | No | No | NA | No | No | No | Yes, without subgroup analysis according to weight/BMI |
|  | Iniparib | II | NCT00540358  doi: 10.1056/NEJMoa1011418 | 123 | TNBC | °Gemcitabine + carboplatin + iniparib °Gemcitabine + cabroplatine | Weight based dosing | °CBR °Safety °Tolerability | °ORR °PFS | No | No | No | NA | No | No | No | No |
|  |  | II | NCT01045304   doi: 10.1007/s10549-019-05305-w | 163 | TNBC | °Gemcitabine + carboplatine + iniparib | Weight based dosing | °ORR | °CBR °PK °PFS °OS | No | No | No | NA | No | No | No | No |
|  |  | II | TBCRC 018  doi: 10.1007/s10549-014-3039-y | 37 | TNBC | °iniparib + irinotecan | Weight based dosing | °TTP | °RR °CBR °OS °Toxicity QoL | No | No | No | NA | No | No | No | No |
|  | Talazoparib | I | NCT03343054  doi: 10.1007/s12282-022-01390-w | 19 | HER2- | Talazoparib | Fixed dose | ORR | °PFS °OS °Safety °PK | No | No | No | NA | No | No | No | No |
|  |  | II | NCT02401347  doi: 10.1038/s43018-022-00439-1 | 20 | not only BC | Talazoparib | Fixed dose | ORR | °CBR °PFS °Safety | No | No | No | NA | No | No | No | No |
|  |  | II | ABRAZO  doi: 10.1158/1078-0432.CCR-18-1891 | 84 | all BC | Talazoparib | Fixed dose | ORR | °DoR °CBR °PFS °OS | No | No | No | NA | No | No | No | Yes, without subgroup analysis according to weight/BMI |
|  |  | II | NCT01989546  doi: 10.1007/s00280-023-04600-0 | 9 | not only BC | Talazoparib | Fixed dose | % pts with PD response | °ORR | No | No | No | NA | No | No | No | Yes, without subgroup analysis according to weight/BMI |
|  |  | I | NCT01286987  doi: 10.1158/2159-8290.CD-16-1250 | 110 | not only BC | Talazoparib | Fixed dose | °MTD °ORR | °PK/PD °PFS °Tumormarker change | No | No | No | NA | No | No | No | No |
|  | Veliparib | I | NCT01853306   doi: 10.1002/cam4.1488 | 71 | not only BC | Veliparib | Fixed dose | °PK °MTD °RP2D | °Safety and °tolerability | No | No | No | NA | No | No | No | Yes, without subgroup analysis according to weight/BMI |
|  |  | I | NCT02210663   doi: 10.1111/cas.13307 | 16 | Not only BC | Veliparib | Fixed dose | °DLT | °AE °response by RECIST °PK | No | No | No | NA | No | No | No | Yes, without subgroup analysis according to weight/BMI |
|  |  | I | NCT01009788  doi: 10.1007/s10549-021-06292-7 | 63 |  | Veliparib + temozolomide | Fixed dose | °ORR °safety | °PFS °CBR | No | No | No | NA | No | No | No | No |
|  |  | I | NCT01477489  doi: 10.1200/JCO.2017.77.2665. | 30 | All BC | Radiotherapy + veliparib | Fixed dose | °DLT | / | No | No | No | NA | No | No | No | No |
|  |  | I | NCT02009631  doi: 10.1007/s00280-016-3156-x. | 47 | Not only BC | Veliparib | Fixed dose | °QTcF | °PK  °AE °Tumor size | No | No | No | NA | No | No | No | Yes, without subgroup analysis according to weight/BMI |
|  |  | II | BROCADE  doi: 10.1093/annonc/mdx505 | 290 | All BC | °Veliparib + Temozolimide °Veliparib + Carboplatine + Paclitaxel °Carboplatin + Paclitaxel + PBO | Fixed dose | °PFS | °OS °ORR | No | No | No | NA | No | No | No | No |
|  |  | I | NCT00526617  doi: 10.1007/s40262-017-0547-z | 42 | Not only BC | Veliparib + temozolomide | Fixed dose | °MTD °Safety and °tolerability °PK | / | No | No | No | NA | No | No | Median Body weight | Yes, without subgroup analysis according to weight/BMI |
|  |  | II | NCT01306032  doi: 10.1158/1078-0432.CCR-14-2565 | 45 | TNBC | °Veliparib + oCyclophosphamide °oCyclophosphamide | Fixed dose | °ORR °PFS | °Safety °Concentration CTCs °nr of pts with deleterious Mut in DNA repair genes | No | No | No | NA | No | No | No | No |
|  |  | II | NCT02595905  doi: 10.1016/S1470-2045(22)00739-2 | 335 | TNBC | °Veliparib + cisplatin °PCB + cisplatin | Fixed dose | °iPFS | °OS °ORR °CBR | No | No | No | NA | No | No | No | No |
|  |  | I/II | NCT01149083  doi: 10.1158/1078-0432.CCR-16-2714. | 71 | All BC | Phase I °Carboplatin + veliparib  Phase II °Veliparib => progression: + carboplatin | Fixed dose | °MTD °Safety and °tolerability °PK | °Toxicity °ORR °PK °Translational | No | No | No | NA | No | No | No | Yes, without subgroup analysis according to weight/BMI |
|  |  | I | NCT01145430  doi: 10.1007/s00280-020-04030-2 | 44 | Not only BC | °Veliparib + liposomal doxorubicin | Fixed dose | °RP2D | °Toxicity °PK | No | No | No | NA | No | No | No | Yes, without subgroup analysis according to weight/BMI |
|  |  | I | NCT00535119  doi: 10.1007/s00280-019-03960-w | 73 | Not only BC | °Paclitaxel + Carboplatin => Paclitaxel + carboplatin + veliparib | Fixed dose | °RP2D | - DLT - preliminary anti-tumor activity - PK | No | No | No | NA | No | No | No | Yes, without subgroup analysis according to weight/BMI |
|  |  | I | NCT01251874  doi: 10.1634/theoncologist.2020-0039 | 44 | HER2- | °Veliparib + carboplatin | Fixed dose | °DLT | - CR | No | No | No | NA | No | No | No | No |
|  |  | I | NCT00576654  doi: 10.1158/1078-0432.CCR-15-0652 | 35 | Not only BC | °irinotecan + veliparib | Fixed dose | °RP2D °Aes °DLT MTD | - PK - ORR | No | No | No | NA | No | No | No | No |
|  |  | I | ETCTN 8620  doi: 10.1007/s10549-023-06889-0 | 30 | TNBC | °veliparib + caboplatin + paclitaxel | Fixed dose | °MTD °Safety and °tolerability °PK | - BRCA levels - PAR and g-H2AX in tumor tissue - AE - PK - ORR | No | No | No | NA | No | No | No | Yes, without subgroup analysis according to weight/BMI |
|  |  | I | NCT01154426  doi: 10.1007/s00280-022-04430-6 | 98 | Not only BC | °veliparib | Fixed dose | °DLT °MTD °RP2D | - Safety and tolerability - PK - PD - Clinical response | No | No | No | NA | No | No | No | Yes, without subgroup analysis according to weight/BMI |
|  |  | I | Tan et al  doi: 10.1007/s00280-021-04350-x | 80 | Not only BC | °veliparib + iv cyclophosphamide °veliparib + iv doxorubicin + iv cyclophosphamide | Fixed dose | °MTD | - PK - PARP activity | No | No | No | NA | No | No | No | Yes, without subgroup analysis according to weight/BMI |
|  |  | I | NCT01063816  doi: 10.1016/j.ygyno.2017.12.029. | 75 | Not only BC | °veliparib + carboplatin + gemcitabin => veliparib maintenance | Fixed dose | °MTD °RP2D | - Safety - tumor assessment - PK | No | No | No | NA | No | No | No | Yes, without subgroup analysis according to weight/BMI |
|  |  | I | NCT01351909  doi: 10.1016/j.clbc.2017.08.013 | 31 | HER2- | °veliparib + ocyclophosphamide | Fixed dose | °safety °RP2D | - AE profile - Clinical efficacy | No | No | No | NA | No | No | No | No |
|  |  | II | Mehta et al  doi: 10.1007/s11060-015-1733-1 | 81 | Not only BC | °WBRT + veliparib | Fixed dose | °safety and °tolerability °MTD °RP2D | - PK - ORR - OS | No | No | No | NA | No | No | No | Yes, without subgroup analysis according to weight/BMI |
| **Tyrosine kinase inhibitors** | | | | | | | | | | | | | | | | | |
| Neoadjuvant | Lapatinib | II | Neo-ALL-IN   doi: 10.1007/s00280-016-3107-6 | 24 | ER+ HER2+ | letrozole + lapatinib | Fixed dose | - PCR | - ORR - RFS - OS - BC surgery rate - Safety | No | No | No | NA | No | No | No | No |
|  |  | Ib | NCT01133912  doi: 10.1007/s10637-011-9759-5 | 12 | HER2+ | lapatinib + paclitaxel + gemcitabine | Fixed dose | - DLT - MTD - Safety | - pCR | No | No | No | NA | No | No | No | No |
|  |  | I | NCT00331630  doi: 10.1007/s10549-011-1411-8 | 30 | HER2+ | lapatinib + nab-paclitaxel | Fixed dose | - cRR | - pCR Tolerability marker response | No | No | No | NA | No | No | No | No |
|  |  | II | PAMELA  doi: [10.1016/S1470-2045(17)30021-9](https://doi.org/10.1016/S1470-2045(17)30021-9) | 151 | HER2+ | lapatininb + trastuzumab +/- et | Fixed dose | - pCR by HER2 enriched subtype | - Gene expression changes - Safety and tolerability - HER2 enriched subtype predictive value | No | No | No | NA | No | No | No | No |
|  |  | II | LPS study  doi: 10.1093/annonc/mds594 | 31 | HER2+ | lapatinib | Fixed dose | - Ki67 apoptosis | - Clinical response - AE - changes in FOXO3a, FOXM1, p-AKT, HER3 | No | No | No | NA | No | No | No | No |
|  |  | II | NCT00524303  doi: 10.1186/1756-0500-6-507 | 100 | HER2+ | °trastuzumab  °lapatinib °trastuzumab + lapatinib | Fixed dose | - pCR | - CCR - DFS - Cardiac function | No | No | No | NA | No | No | No | No |
|  |  | II | TBCRC 006  doi: 10.1200/JCO.2012.44.8027 | 64 | HER2+ | lapatinib + endocrine treatment | Fixed dose | - PCR | - biomarkers | No | No | No | NA | No | No | No | No |
|  |  | II | GEICAM/2006-14  doi: 10.1038/bjc.2013.831. | 102 | HER2+ | °epirubicin + cyclophosphamide + docetaxel + Trastuzumab  °epirubicin + cyclophosphamide + docetaxel+ lapatinib | Fixed dose | - pCR | - toxicity - ORR | No | No | No | NA | No | No | No | No |
|  |  | II | Cher-LOB  doi: 10.1200/JCO.2011.39.0823 | 121 | HR+ HER2- | Lapatinib + letrozole  letrozole + PCB | Fixed dose | - ORR | - PCR - OS - PFS - Bcsurgery rate - safety - TTF - Biomarkers and GEPs | No | No | No | NA | No | No | No | No |
|  |  | II | TBCRC 023  doi: 10.1158/1078-0432.CCR-19-0851 | 97 | HER2+ | Lapatinib + trastuzumab | Fixed dose | - PCR breast | - Safety | No | No | No | NA | No | No | No | No |
|  |  | II | TRIO-US B07  doi: 10.1038/s41467-020-19494-2 | 128 | HER2+ | °trastuzumab + carboplatin + docetaxel °lapatinib + carboplatin + docetaxel °trastuzumab + lapatinib + carboplatin + docetaxel | Fixed dose | - pCR | - Safety and tolerability | No | No | No | NA | No | No | No | No |
|  |  | IIb | DeCensi et al  doi: 10.1200/JCO.18.01779 | 56 | HER2+ | lapatinib pcb | Fixed dose | - Ki67 change | - Safety | No | No | Yes | cohort A) BMI 25.5 mg/kg^2^ B) 24.4 kg/m^2^ | No | No | No | No |
|  |  | II | JBCRG-16 [Neo-LaTH]  doi: 10.3390/cancers13164008 | 215 | HER2+ | °Arm A: 18w lapatinib + 18w Trastuzumab + 12w paclitaxel  °arm B: 30w lapatinib + 30w trastuzumab + 12w paclitaxel °Arm C: 18w lapatinib + 18w trastuzumab + 12w paclitaxel  °Arm D: 18w lapatinib + 18w trastuzumab + 18w ET + 12w Paclitaxel °Arm E: 30w lapatinib + 30w trastuzumab + 30w ET + 12w pacltaxel | Fixed Dose | - CpCR | - ORR - Bcsurgery rate - CpCR + yN0 | No | No | Yes | BMI cohort A) 21.7 kg/m^2^ B)22.9 kg/m^2^ C)21.8 kg/m^2;^ D)21.5 kg/m^2^; E)21.8 kg/m^2^ | No | No | No | No |
|  |  | II | UK EPHOS-B trial  doi: 10.1158/1078-0432.CCR-21-3177 | 257 | HER2+ | part 1:  °no preoperative treatment °trastuzumab °lapatinib  part 2:  °no preoperative treatment °lapatinib + trastuzumab °trastuzumab | Fixed Dose | - Ki67 apoptosis | - RFS - OS - Safety | No | No | No | NA | No | No | No | No |
|  |  | II | Estevez et al  doi: 10.1186/bcr3695 | 20 | HER2+ | lapatinib | Fixed dose | - effect on biology | - tumor changes on MRI | No | No | No | NA | No | No | No | No |
|  |  | II | RC0639  doi: 10.1007/s10549-013-2469-2 | 109 | HER2+ | 4x doxorubicin + cyclophosphamideq2-3w => 12w trastuzumab q3w + paclitaxel q1w + lapatinib => 40w trastuzumab q3w + lapatinib | Fixed dose | - Cardiac function | - safety | No | No | No | NA | No | No | No | No |
|  |  | II | Boussen et al  doi: 10.1200/JCO.2009.21.8594 | 49 | all BC subtypes | lapatinib + paclitaxel | Fixed dose | - pCR | - CRR | No | No | No | NA | No | No | Median weight | No |
|  |  | II | EORTC 10054  doi: 10.1093/annonc/mdu551 | 128 | HER2+ | °chemotherapy + lapatinib °chemotherapy + trastuzumab °chemotherapy + lapatinib + trastuzumab | Fixed dose | - PCR | - safety and toxicity | No | No | No | NA | No | No | No | No |
|  |  | II | NCT00826267  doi: [10.1016/j.clbc.2014.11.004](https://doi.org/10.1016/j.clbc.2014.11.004) | 29 | HER2+ | °Afatinib  °Lapatinib  °Trastuzumab | Fixed dose | - ORR | - CBR | No | No | No | NA | No | No | No | Mentioned in objectives though no results reported |
|  | Neratinib | II | I-SPY2  doi: 1 0.1056/NEJMoa1513750 | 193 | All BC subtypes | °neratinib + chemotherapy °trastuzumab + chemotherapye | Fixed dose | - pCR | - EFS OS RCB | No | No | No | NA | No | No | No | No |
|  |  | I | NSABP FB-7, part I  doi: 10.1007/s00280-013-2262-2 | 21 | HER2+ | neratinib + trastuzumab + paclitaxel | Fixed dose | - RP2D | - ORR TTP | No | No | No | NA | No | No | No | No |
|  |  | II | NSABP FB-7, part II  doi: 10.1186/s13058-019-1196-y | 126 | HER2+ | °Neratinib + trastuzumab + paclitaxel => doxorubicin + cyclophosphamide °Trastuzumab + paclitaxel => doxorubicin + cyclophosphamide °Neratinib + paclitaxel => doxorubicin + cyclophosphamide | Fixed dose | - pCR | - CCR pCR breast Aes | No | No | No | NA | No | No | No | No |
|  | Pyrotinib | II | NeoATP  doi: 10.1158/1078-0432.CCR-22-0446 | 53 | HER2+ | Pyrotinib + trastuzumab + paclitaxel/cisplatin | Fixed dose | - pCR, ypT0 N0 | - pCR ypT0/isN0 °Biomarker °Safety | No | No | No | NA | No | No | No | No |
|  |  | II | NCT04152057  doi: 10.21037/gs-21-911 | 21 | HER2+ | pyrotinib + trastuzumab + nab-paclitaxel | Fixed dose | - pCR, ypT0 N0 | - ORR °AE | No | No | No | NA | No | No | No | No |
|  |  | II | NCT04486911  doi: 10.1038/s41467-022-34838-w | 81 | TNBC | Pyrotinib + letrozole + dalpiciclib | pyrotinib + dalpiciclib: Fixed dose | - pCR, ypT0 N0 | - RCB °ORR °breast pcr °safety °changes in Ki67 | No | No | No | NA | No | No | No | No |
|  |  | II | Xuhong et al, 2020  doi: [10.1002/onco.13546](https://doi.org/10.1002/onco.13546) | 20 | HER2+ | 8x Pyrotinib + EC => Docetaxel + trastuzumab | Fixed dose | - pCR | - ORR °Safety °TIL °RCB °Neo-bioscore | No | No | No | NA | No | No | No | No |
|  |  | II | PANPHILA  doi: 10.1016/j.ejca.2022.01.022 | 69 | HER2+ | Pyrotinib + trastuzumab + carboplatin + docetaxel | Fixed dose | - pCR | - ORR °EFS °DFS °OS °safety | No | No | No | NA | No | No | No | No |
|  |  | II | Ding et al  doi: 10.1159/000531492 | 69 | HER2+ | °pyrotinib + trastuzumab + docetaxel + caboplatin ° trastuzumab + docetaxel + caboplatin | Fixed dose | - pCR | - ORR s°afety | No | No | No | NA | No | No | No | No |
| Adjuvant | neratinib | II | NCT02400476  doi: 10.1016/j.annonc.2020.05.012 | 69 | HER2+ | °neratinib + loperamide prophylaxis | Fixed dose | - Incidence of diarrhea grade III | - AEs Diarrhea severity | No | No | No | NA | No | No | No | No |
| Metastatic | lapatinib | II | NCT00820924  doi: 10.1007/s10549-012-2045-1 | 16 | HER2- | - lapatinib | Fixed dose | - ORR | - CBR - TTTP - Safety - effect on CTCs | No | No | No | NA | No | No | No | No |
|  |  | I | EGF10027  doi: 10.1371/journal.pone.0142845. | 28 | HER2+ | - lapatinib | Fixed dose | - biomarker | - AEs - Changes in labvalues | No | No | Yes | BMI cohort A) 30 kg/m^2^, B) 31 kg/m^2^ C) 24 kg/m^2^ | No | No | No | Yes, without subgroup analysis according to weight/BMI |
|  |  | Ib | NCT02131506  doi: 10.1007/s00280-017-3279-8 | 11 | HER2+ | - caelyx + lapatinib | Fixed dose | - MTD | - Safety - ORR - CBR | No | No | No | NA | No | No | No | No |
|  |  | II | NCT00263588  doi: [10.1158/1078-0432.CCR-08-1080](https://doi.org/10.1158/1078-0432.CCR-08-1080) | 242 | HER2+ | - lapatinib | Fixed dose | - CNS ORR | - Safety - Tolerability - Neurologic signs and symptoms - PFS - OS | No | No | yes | Mean BMI 27 kg/m^2^ | No | No | No | Yes, without subgroup analysis according to weight/BMI |
|  |  | I | NCT00996762  doi: 10.1002/cpdd.139 | 68 | HER2+ | - lapatinib | Fixed dose | - PK | - Safety - Questionnaires regarding the different formulations (taste/consistency etc) | No | No | No | NA | No | No | Median weight | Yes, without subgroup analysis according to weight/BMI |
|  |  | II | EGF103009  doi: 10.1016/S1470-2045(09)70087-7 | 153 | HER2+ | - lapatinib | Fixed dose | - ORR | - CBR - PFS - TTR - DoR - OS - QoL | No | No | No | NA | No | No | Median and máximum weight | Yes, without subgroup analysis according to weight/BMI |
|  |  | I, II | SWOG S0528  doi: 10.1007/s00280-013-2297-4 | 80 | Not only BC | - lapatinib - lapatinib + everolimus | Fixed dose | - PK DLT | - PK | No | No | No | NA | No | No | No | No |
|  |  | I | EGF111582  doi: 10.1007/s10637-013-0055-4 | 25 | Not only BC | - lapatinib | Fixed dose | - PK | - Safety - tolerability | No | No | No | NA | No | No | No | Yes, without subgroup analysis according to weight/BMI |
|  |  | II | NCT00558103  doi: 10.1007/s10549-012-2369-x | 164 | HER2+ | - lapatinib + pcb - lapatinib + pazopanib | Fixed dose | - ORR | - DOR - PFS - OS - Safety | No | No | No | NA | No | No | No | No |
|  |  | II | NCT00444535  doi: 10.1007/s10549-011-1918-z | 52 | HER2+ | - lapatinib + bevacuzimab | Fixed dose | - PFS12w | - ORR - CBR - DOR - TTR - PFS - Safety | No | No | No | NA | No | No | No | No |
|  |  | II | NCT00694252  doi: 10.1371/journal.pone.0123683 | 22 | HER2+ | - lapatinib | Fixed dose | - effect of lapatinib on CTC | - kinetics of CTC - toxicity | No | No | No | NA | No | No | No | No |
|  |  | II | NCT00709618  doi: 10.1186/2193-1801-3-108 | 44 | HER2+ | - lapatinib + vinorelbine | Fixed dose | - ORR | - PFS - OS - DoR - TTR - TTP | No | No | No | NA | No | No | No | No |
|  |  | Ib | EGF100161  doi: [10.1093/annonc/mdt222](https://doi.org/10.1093/annonc/mdt222) | 53 | HER2+ | - lapatinib + trastuzumab + docetaxel | Fixed dose | - Optimally tolerated regimen - Safety - tolerability | - ORR - PK | No | No | No | NA | No | No | No | Yes, without subgroup analysis according to weight/BMI |
|  |  | II | NCT00709761  10.1007/s10549-012-2341-9 | 32 | HER2+ | - lapatinib + nab-paclitaxel | Fixed dose | - ORR | - PFS - OS - DOR - TTR - TTP | No | No | No | NA | No | No | No | No |
|  |  | Ib | PIKHER2  doi: 10.1016/j.ejca.2017.08.025 | 24 | HER2+ | - lapatinib + buparlisib | Fixed dose | - MTD - RP2D | - PK - ORR - CBR - DCR | No | No | No | NA | No | No | No | Yes, without subgroup analysis according to weight/BMI |
|  |  | II | VEG20007  doi: 10.1007/s10549-012-2399-4 | 190 | HER2+ | - lapatinib + pazopanib  lapatinib | Fixed dose | - Progressive disease rate 12w | - response rate 12w OS - safety - tolerability | No | No | No | NA | No | No | No | No |
|  |  | II | NCT00356811  doi: 10.1159/000318043 | 57 | HER2+ | - lapatinib + paclitaxel | Fixed dose | - ORR | - DoR - TTR - TTP - PFS - OS - Safety | No | No | No | NA | No | No | No | No |
|  |  | I | NCT00388076  doi: 10.1038/bjc.2014.233 | 26 | Not only BC | - paclitaxel + pazopanib + lapatinib | Fixed dose | - DLT | - safety profile - PK - ORR | No | No | No | NA | No | No | No | Yes, without subgroup analysis according to weight/BMI |
|  |  | II | TBCRC 003  doi: 10.1200/JCO.2014.60.0353 | 78 | HER2+ | - lapatinib + trastuzumab | Fixed dose | - ORR | - CBR - PFS | No | No | No | NA | No | No | No | No |
|  |  | II | KCSG BR11-16  doi: 10.1038/s41416-019-0618-z | 149 | HER2+ | - lapatinib + vinorelbine vinorelbine | Fixed dose | - PFS 18w | - ORR - OS - PFS - Safety | No | No | No | NA | No | No | No | No |
|  |  | II | NCT00777101  doi: 10.1016/j.ejca.2013.07.142. | 233 | HER2+ | - lapatinib + capecitabine neratinib | Fixed dose | - PFS | - OS - ORR - CBR - DOR - CNS Metastasis time to CNS metastasis | No | No | No | NA | No | No | No | No |
|  |  | I/II | NCT01138046  doi: 10.1007/s10147-015-0832-5 | 12 | HER2+ | - paclitaxel + lapatinib | Fixed dose | - Tolerability - OS | - PK - PFS - ORR - CBR - Safety - Biomarkers | No | No | No | NA | No | No | No | Yes, without subgroup analysis according to weight/BMI |
|  |  | I | NCT01934894  doi: 10.1016/j.clbc.2018.03.004 | 11 |  | - lapatinib + cabazitaxel | Fixed dose | - Safety - ORR CNS | - CBR CNS PFS ORR extracranial | No | No | No | NA | No | No | No | No |
|  |  | I, II | NCT00912275  doi: [10.1093/jjco/hyx188](https://doi.org/10.1093/jjco/hyx188) | 46 |  | - lapatinib + vinorelbine | Fixed dose | - phase I: DLT - Phase II: PFS | - ORR - OS - CBR - Safety | No | No | No | NA | No | No | No | No |
|  |  | I | Lapatam  doi: 10.1016/j.breast.2014.07.003 | 25 |  | - tamoxifen => tamoxifen + lapatinib - lapatinib => tamoxifen + lapatinib | Fixed dose | - PK | - Safety - ORR - PFS | No | No | No | NA | No | No | No | Yes, without subgroup analysis according to weight/BMI |
|  |  | I | NCT00952692  doi: 10.1186/1479-5876-10-28 | 12 | HER2+ | - lapatinib + dHER2 ASCI injections | Fixed dose | - Safety - Tcell response | - ORR - PFS - OS | No | No | No | NA | No | No | No | No |
|  |  | Ib/II | TRIO-US B09  doi: 10.1038/s41467-020-19494-2 | 19 | HER2+ | - lapatinib + everolimus + capecitabine | Fixed dose | - ORR | - Safety - PFS - OS - best CNS ORR - best extra CNS - ORR | No | No | No | NA | No | No | No | No |
|  |  | II | NCCTG N0733  doi: 10.1007/s10549-021-06221-8 | 68 | HER2+ | - capecitabine + lapatinib - capecitabine + lapatinib + cituxumab | Fixed dose | - PFS | - OS - TTF - Safety - QoL - Fatigue - Rash - Diarrhea - Hand-foot syndrome | No | No | No | NA | No | No | No | No |
|  |  | I | NCT01199367  doi: 10.1177/1758835918786858 | 11 | HER2+ | - lapatinib + letrozole | Fixed dose | - Safety and tolerability - RP2D | - PK/PD | No | No | No | NA | No | No | Average height and weight of sample | Yes, without subgroup analysis according to weight/BMI |
|  |  | II | NCT02422199  doi: 10.1038/s41392-023-01322-w | 128 | HER2+ | - pyrotinib + capecitabine - lapatinib + capecitabine | Fixed dose | - ORR | - PFS - Safety | No | No | No | NA | No | No | No | No |
|  |  | II | NCT01688609  doi: 10.18632/oncotarget.23914 | 18 | HER2+ | - lapatinib + trastuzumab + paclitaxel | Fixed dose | - Biomarkers - PCR - Safety | - ORR | No | No | No | NA | No | No | No | No |
|  |  | II | NCT01013740  doi: 10.1016/j.breast.2015.08.005. | 112 | HER2+ | - lapatinib + vinorelbine - lapatinib + capecitabine | Fixed dose | - PFS | - OS | No | No | No | NA | No | No | No | No |
|  |  | Ib | NCT01434303  doi: 10.1038/s41416-019-0473-y | 13 | HER2+ | - etinostat + lapatinib - etinostat + lapatinib + trastuzumab | Fixed dose | - Safety - MTD | - PD - ORR - OS - PFS | No | No | No | NA | No | No | No | No |
|  |  | II | SYSUCC-005  doi: 10.1186/s12885-022-09399-2 | 159 | HER2+ | - lapatinib + capecitabine/vinorelbine | Fixed dose | - PFS | - OS - RR - CBR - Safety | No | No | No | NA | No | No | No | No |
|  |  | I | LAPTEM trial  doi: 10.1093/annonc/mdt359 | 18 | HER2+ | - lapatinib + temozolomide | Fixed dose | - DLTs - MTD | - ORR - CBR - DOR | No | No | No | NA | No | No | No | No |
|  |  | II | NCT01622868  doi: [10.1016/j.ijrobp.2020.07.953](https://doi.org/10.1016/j.ijrobp.2020.07.953) | 143 | HER2+ | °WBRT or SRS + lapatinib  °WBRT of SRS | Fixed dose | - Intracranial - CRR | - ORR - Lesion specific RR - CNS PFS - OS | No | No | No | NA | No | No | No | No |
|  |  | I | NCT00544804  doi: 10.1200/JCO.2013.52.1161 | 40 | HER2+ | - lapatinib | Fixed dose | - MTD | - PK - clinical RR | No | No | No | NA | No | No | No | Yes, without subgroup analysis according to weight/BMI |
|  |  | II | LANDSCAPE  doi: 10.1016/S1470-2045(12)70432-1 | 45 | HER2+ | - lapatinib + capecitabine | Fixed dose | - RR CNS | - TTP - Time to   radiotherapy  °ORR CNS and extra CNS | No | No | No | NA | No | No | No | No |
|  |  | I | NCT02650752  doi: 10.1158/1078-0432.CCR-18-3502 | 11 | HER2+ | - lapatinib + capecitabine | Fixed dose | - Safety - MTD | - CTC assessment in CSF - Completion rate - ORR | No | No | No | NA | No | No | No | No |
|  |  | II | LANTERN  doi: 10.1016/j.clon.2020.06.003 | 30 | HER2+ | - lapatinib + capecitabine - lapatinib + trastuzumab | Fixed dose | - TTP of CNS M | - PFS - OS - CNS ORR - CNS CBR - Steroid use - QoL | No | No | No | NA | No | No | No | No |
|  |  | II | Xu et al  doi: [10.1634/theoncologist.2020-0044](https://doi.org/10.1634/theoncologist.2020-0044) | 10 | HER2+ | - lapatinib + pc chemotherapy | Fixed dose | - PK | - Safety | No | No | No | NA | No | No | No | Yes, without subgroup analysis according to weight/BMI |
|  |  | II | UMIN000005219  doi: 10.1016/j.breast.2018.04.010 | 86 | HER2+ | - laptinib + capecitabine trastuzumab + capecitabine | Fixed dose | - PFS | - OS - ORR | No | No | No | NA | No | No | No | No |
|  |  | II | HE42/09  doi: 10.1007/s11060-017-2548-z. | 81 | Not only BC | - Lapatinib + WBRT | Fixed dose | - ORR CNS | - OS - TTP - Safety - tolerability | No | No | No | NA | No | No | No | No |
|  |  | Ib | Chia et al  doi: 10.1186/s13058-017-0836-3 | 19 | HER2+ | - lapatinib + foretinib | Fixed dose | - RP2D - Safety profile | - PK - ORR - c-Met in tumor specimen | No | No | No | NA | No | No | No | Yes, without subgroup analysis according to weight/BMI |
|  |  | I | Wisinski et al  doi: 10.1158/1078-0432.CCR-15-2365. | 28 | not only BC | - MK-2206 + lapatinib | Fixed dose | - MTD - DLT - safety | - PK/PD - ORR | No | No | No | NA | No | No | No | Yes, without subgroup analysis according to weight/BMI |
|  |  | II | CECOG LaVie  doi: 10.1186/s12885-016-2171-y | 9 | HER2+ | - lapatinib + vinorelbine | Fixed dose | - PFS | - OS - ORR - Safety | No | No | No | NA | No | No | No | No |
|  |  | II | NCT01050322  doi: 10.1002/14651858 | 142 | HER2+ | - lapatinib + capecitabine - lapatinib + vinorelbine - lapatinib + gemcitabine | Fixed dose | - ORR | - PFS - OS - DoR - safety | No | No | No | NA | No | No | No | No |
|  |  | I | van der Noll et al  doi: 10.1007/s10637-015-0281-z | 33 | HER2+ | - lapatinib + gemcitabine | Fixed dose | - Safety - Tolerability - MTD - DLT | - PK | No | No | No | NA | No | No | No | Yes, without subgroup analysis according to weight/BMI |
|  |  | II | NCT00903656  no doi;  Anticancer research, 2015, Pircher et al | 24 | HER2+ | - lapatinib + pegylated liposomal doxorubicin | Fixed dose | - ORR | - Safety - QoL - occurence CNS M - OS - PFS - CBR | No | No | No | NA | No | No | No | No |
|  |  | II | Chan et al  doi: [10.1111/ajco.12292](https://doi.org/10.1111/ajco.12292) | 19 | HER2+ | - lapatinib + vinorelbine | Fixed dose | - PFS | - ORR - Pain - Safety - OS | No | No | No | NA | No | No | No | No |
|  |  | II | CALGB 40302  doi: 10.1200/JCO.2014.56.7941 | 295 | HR+ | - fulvestrant + lapatinib - fulvestrant + PCB | Fixed dose | - PFS | - Toxicity - ORR - OS | No | No | No | NA | No | No | No | No |
|  |  | II | Shawky et al  doi: 10.1016/j.jnci.2014.08.001. | 21 | HER2+ | - lapatinib + capecitabine | Fixed dose | - ORR | - PFS - OS - toxicity | No | No | No | NA | No | No | No | No |
|  |  | II | NU Lin et al, 2013  doi: 10.1007/s10549-013-2754-0 | 35 | HER2+ | - lapatinib + WBRT | Fixed dose | - MTD | - ORR CNS - ORR non-CNS - PFS - OS - Site of first progression - PFS6m - Cause of death | No | No | No | NA | No | No | No | No |
|  |  | I | NCT00543504  doi: 10.1093/annonc/mdt395 | 26 | HER2+ | - lapatinib + trastuzumab + bevacuzimab | Fixed dose | - RP2D - Safety profile | - ORR | No | No | No | NA | No | No | No | No |
|  |  | II | Saip et al  doi: 10.1016/j.breast.2013.07.048 | 29 | HEr2+ | - lapatinib | Fixed dose | - ORR CBR | - Toxicity DoR PFS | No | No | No | NA | No | No | No | No |
|  |  | II | Villanueva et al  doi: 10.1007/s11523-013-0279-4 | 24 | HR+ HER2- | - letrozole + lapatinib | Fixed dose | - ORR 12w | - TTR DoR CBR PFS OS Safety | No | No | No | NA | No | No | No | No |
|  |  | II | Iwata et al  doi: 10.1007/s10147-012-0444-2 | 6 | HER2+ | - lapatinib | Fixed dose | - Safety | - PK | No | No | No | NA | No | No | No | No |
|  |  | I | Kimple et al  doi: 10.1634/theoncologist.2012-0256 | 19 |  | - lapatinib | Fixed dose | - Toxicity and safety MTD | - effect on EGFR and HER2 pathways | No | No | No | NA | No | No | No | No |
|  |  | II | Crivellari et al  doi: 10.1177/030089161209800104 | 68 | HER2+ | - lapatinib + capecitabine | Fixed dose | - PFS | - OS ORR safety | No | No | No | NA | No | No | No | No |
|  |  | I | Brain et al  doi: 10.1038/bjc.2011.591 | 33 | HER2+ | - lapatinib + vinorelbine | Fixed dose | - MTD - DLT - Max administered dose | °Safety | No | No | No | NA | No | No | No | Yes, without subgroup analysis according to weight/BMI |
|  |  | II | Gajria et al  doi: 10.1586/era.10.226 | 23 | HER2+ | - lapatinib + capecitabine | Fixed dose | - ORR | - Toxicity - PFS - % pts Stable disease ≥ 6m | No | No | No | NA | No | No | No | No |
|  |  | II | NU Lin et al, 2011  doi: 10.1007/s11060-011-0629-y | 22 | HER2+ | - lapatinib + capecitabine - lapatinib + topotecan | Fixed dose | - ORR CNS | - Safety - Toxicity - % pts Stable disease ≥ 6m - CBR - DoR CNS - % pts with NSS improvement - ORR in non-CNS - Site of disease progression - TTP - OS | No | No | No | NA | No | No | No | No |
|  |  | II | Blackwell et al  doi: 10.1093/annonc/mdn759 | 79 | HER2+ | - lapatinib | Fixed dose | - ORR | - CBR - TTP - DoR - PFS - OS - Safety | No | No | No | NA | No | No | No | No |
|  |  | I | Chu et al  doi: 10.1158/1078-0432 | 39 pts, 18 BC | Not only BC | - lapatinib + letrozole | Fixed dose | - PK - DLT - Optimally tolerated regimen | - Safety | No | No | No | NA | No | No | No | Yes, without subgroup analysis according to weight/BMI |
|  |  | I | Storniolo et al   doi: 10.1200/JCO.2007.13.5202 | 54 | HER2+ | - lapatinib + trastuzumab | Fixed dose | - Safety - Feasibility - OTR | - PK | No | No | No | NA | No | No | No | No |
|  |  | II | Burstein et al  [10.1093/annonc/mdm601](https://doi.org/10.1093/annonc/mdm601) | 229 | All BC | - lapatinib | Fixed dose | - ORR | - CBR - TTP - PFS - OS - safety | No | No | No | NA | No | No | No | No |
|  | Neratinib | I | NCT00146172  doi: 10.1158/1078-0432 | 60 | Not only BC | - neratinib | Fixed dose | - DLT °MTD | - ORR - DoR - Best overall response - PFS - CBR | No | No | No | NA | No | No | No | Yes, without subgroup analysis according to weight/BMI |
|  |  | I/II | NCT00741260  doi: 10.1200/JCO.2014.56.3809 | 105 | HER2+ | - neratinib + capecitabine | Fixed dose | - MTD - ORR | - CBR - PFS - DoR | No | No | No | NA | No | No | No | No |
|  |  | I/II | NCT00706030   doi: 10.1093/annonc/mds284 | 91 | Her2+ | - neratinib + vinorelbine | Fixed dose | - MTD - Safety - ORR | - PK - PFS - CBR - DoR | No | No | No | NA | No | No | No | Yes, without subgroup analysis according to weight/BMI |
|  |  | I/II | NCT00445458  doi: 10.1038/bjc.2013.178 | 110 | HER2+ | - neratinib + paclitaxel | Fixed dose | - MTD - DLT - ORR | - PK - Safety - CBR - PFS - DoR | No | No | No | NA | No | No | No | No |
|  |  | II | NEfERT-T  doi: 10.1038/s41467-023-44140-y | 479 | HER2+ | - neratinib + paclitaxel + trastuzumab | Fixed dose | - PFS | - RR - CBR - DoR - Frequency - TTP CNS - Safety | No | No | No | NA | No | No | No | No |
|  |  | II | TBCRC 022  doi: 10.1200/JCO.18.01511 | 94 | HER2+ | - neratinib + capecitabine | Fixed dose | - CNS ORR | - CNS response - RANO-BM - PFS - Site of first progression - extra CNS ORR - OS - Toxicity | No | No | No | NA | No | No | No | No |
|  |  | II | SUMMIT  [10.1016/j.annonc.2023.08.003](https://doi.org/10.1016/j.annonc.2023.08.003) | 81 | HER2+ | - neratinib neratinib+fulvestrant neratinib + fulvestrant + trastuzumab | Fixed dose | - ORR | - CBR - DoR - PFS | No | No | No | NA | No | No | No | No |
|  |  | II | NCT02673398 doi:10.1016/  j.jgo.2021.02.020 | 25 | HER2+ or HER2/3 mut | - neratinib | Fixed dose | - Safety | - Reduction rates - PK - ORR - PFS - OS | No | No | No | NA | No | No | No | Yes, without subgroup analysis according to weight/BMI |
|  |  | II | Ma et al doi:10.1158/  1078-0432.CCR-21-3418 | 35 | HER2- | - neratinib + fulvestrant | Fixed dose | - CBR | - PFS - Safety and - tolerability | No | No | No | NA | No | No | No | No |
|  |  | I/II | NSABP Foundation Trial FB-10 doi:10.1200/  JCO.19.00858 | 27 | HER2+ | - T-DM1 + neratinib | °T-DM1: weight-based dose  °neratinib: fixed dose | - RP2D - DLT | - ORR - DOR - Toxicity | No | No | No | NA | No | No | No | Yes, without subgroup analysis according to weight/BMI |
|  |  | I/II | Blackwell et al doi:10.1016/  j.clbc.2018.12.011 | 45 | HER2+ | - neratinib + trastuzumab | Fixed dose | - DLT - PFS 16w | - Safety - ORR - CBR - PFS - DoR - PK | No | No | No | NA | No | No | No | Yes, without subgroup analysis according to weight/BMI |
|  |  | II | NCT01670877 doi:10.1158/  1078-0432.CCR-17-0900 | 16 | HER2- | - neratinib | Fixed dose | - CBR | - PFS - Safety - HER2mut - ctDNA | No | No | No | NA | No | No | No | No |
|  |  | II | plasmaMATCH doi:10.1016/  S1470-2045(20)30444-7 | 1051 | All BC subtypes | - fulvestrant - neratinib +/- fulvestrant - capivasertib + fulvestrant - capivasertib | neratinib: fixed dose  °capivasertib: fixed dose | ORR | - Toxicity | No | No | No | NA | No | No | No | Yes, without subgroup analysis according to weight/BMI |
|  |  | I | NCT00838539 doi:10.1200/  JCO.2012.47.2787 | 60 | not only BC | - neratinib + temsirolimus | Fixed dose | - toxicity - RP2D | - PK - ORR | No | No | No | NA | No | No | No | Yes, without subgroup analysis according to weight/BMI |
|  |  | II | NCT00300781 doi:10.1200/  JCO.2009.25.8707 | 136 | HER2+ | - neratinib | Fixed dose | - PFS 16w | - ORR - AEs | No | No | No | NA | No | No | No | Yes, without subgroup analysis according to weight/BMI |
|  | pyrotinib | I | NCT01937689 doi:10.1200/  JCO.2016.69.6179 | 18 | HER2+ | - pyrotinib | Fixed dose | - MTD | - Safety ORR PK | No | No | No | NA | No | No | No | Yes, without subgroup analysis according to weight/BMI |
|  |  | II | NCT03412383 doi:10.1038/  s41523-020-00201-9 | 1184 | HER2- | - pyrotinib | Fixed dose | - PFS | - Aes - ORR - CBR - TTP - QoL - OS | No | No | No | NA | No | No | No | No |
|  |  | II | PICTURE doi:10.1186/  s12916-023-02999-0 | 100 | HER2+ | - pyrotinib | Fixed dose | - PFS | - ORR - DoR - DCR - OS - Safety | No | Yes, severe diabetes | No | NA | No | No | No | No |
|  |  | II | PLEHERM  doi:10.1186/  s12916-023-02943-2 | 53 | HER2+ | - pyrotinib + letrozole | Fixed dose | - CBR | - ORR - PFS - AEs | No | Yes, severe diabetes | No | NA | No | No | No | No |
|  |  | II | PERMEATE doi:10.1016/  S1470-2045(21)00716-6 | 78 | HER2+ | - pyrotinib + capecitabine | Fixed dose | - ORR CNS | - ORR non-CNS - DCR CNSDoR - PFS - OS - Safety | No | No | No | NA | No | No | No | No |
|  |  | II | PANDORA doi:10.1038/  s41467-023-44140-y | 79 | HER2+ | - pyrotinib + docetaxel | Fixed dose | - ORR | - PFS - DoR - CBR - OS - Safety | No | No | No | NA | No | No | No | No |
|  |  | I | NCT02361112 doi:10.1016/  j.eclinm.2023.102314 | 28 | HER2+ | - pyrotinib + capecitabine | Fixed dose | - DLT MTD Safety | - PK - ORR - PFS - biomarkers | No | Yes, severe diabetes | No | NA | No | No | No | Yes, without subgroup analysis according to weight/BMI |
|  |  | II | NCT04582968 doi:10.1016/  j.ijrobp.2023.06.641 | 40 | HER2+ | - Pyrotinib + capecitabine + stereotactic radiotherapy or WBRT | Fixed dose | - PFS CNS | - ORR CNS - PFS - OS - Safety - Changes in neurocognitive function | No | No | No | NA | No | No | No | No |
|  |  | II | He et al doi:10.1016/  j.breast.2023.103581 | 50 | HER2+ | - pyrotinib + capecitabine | Fixed dose | - PFS | - ORR - OS - CBR - Safety | No | No | No | NA | No | No | No | No |
|  |  | II | Xie et al doi:10.1007/  s10549-022-06770-6 | 40 | HER2+ | - pyrotinib + trastuzumab + chemotherapy | Fixed dose | - PFS | - PFS CNS - ORR - CBR - DCR - Safety | No | No | No | NA | No | No | No | No |
| **PIK3CA/AKT/PTEN inhibitor** | | | | | | | | | | | | | | | | | |
| Neoadjuvant | everolimus | II | NECTAR doi:10.1038/  s41598-020-80081-y | 24 | TNBC | °anthracyclulines + taxane, if residual disease > 1cm => everolimus + cisplatin | Fixed dose | - RCB | - Safety | No | No | No | NA | No | No | No | No |
|  |  | II | NCT00499603 doi:10.1093/  annonc/mdu124 | 62 | TNBC | °paclitaxel => 5-FU + epirubicin + cyclophosphamide °paclitaxel + everolimus => 5-FU + epirubicin + cyclophosphamide | Fixed dose | - proportion of pts with inhibition of mTOR pathway 48h | - ORR 12w - ORR 24w | No | Yes, uncontrolled diabetes | No | NA | No | No | No | No |
|  |  | II | NCT00930930 doi:10.1158/  1078-0432.CCR-16-3055 | 145 | TNBC | °cisplatin + paclitaxel + everolimus °cisplatin + paclitaxel + PCB | Fixed dose | - pCR | - CBR - Safety - biomarkers - BCSurgery rate | No | No | No | NA | No | No | No | No |
|  |  | II | RADHER doi:10.1016/  j.ejca.2021.09.017 | 82 | HER2+ | °trastuzumab  °trastuzumab + everolimus | Fixed dose | - CRR | - pCR - Safety | No | No | No | NA | No | No | No | No |
|  |  | II | NCT02742051  doi:10.1186/  s12885-021-08612-y | 40 | ER+ HER2- | °everolimus + letrozole  °fluorouracil + epirubicin + cyclophosphamide | Fixed dose | - Feasibility | - US response rate - pCR - Bcsurgery rate - Toxicity - immune modulation - biomarkers | No | No | No | NA | No | No | No | No |
|  |  | II | NCT00107016 doi:10.1200/  JCO.2008.18.8391 | 270 | ER+ | °letrozole + everolimus °letrozole | Fixed dose | - Clinical response by palpation | - US/Mammography response | No | yes, uncontrolled diabetes | No | NA | No | No | No | Mentioned in objectives, though not reported in results |
|  |  | I/II | NCT02520063 doi:10.1007/  s10549-023-06864-9 | 15 | HR+ HER2- | °everolimus + letrozole + carotuximab | Fixed dose | - Safety and tolerability  RP2D | - PK/PD | No | No | No | NA | No | No | No | Yes, without subgroup analysis according to weight/BMI |
|  | alpelisib | II | NEO-ORB doi:10.1158/  1078-0432.CCR-18-3160 | 257 |  | °letrozole + placebo  °letrozole + alpelisib | Fixed dose | - ORR - pCR | - PEPI score °Safety | No | Yes,  1. Patient with type 1 diabetes or uncontrolled type 2 diabetes (HbA1c </=8%) | No | NA | No | No | No | No |
|  | buparlisib | II | NCT01816594 doi:10.1016/  j.ejca.2017.08.020 | 50 | HER2+ BC | °Buparlisib + trastuzumab=> Buparlisib + trastuzumab + paclitaxel °Placebo + trastuzumab=> placebo + trastuzumab + paclitaxel | Fixed dose | - pCR | - ORR 6w | No | No | Yes | BMI cohort A)  25,1,- kg/m^2^ B) 23,9 kg/m^2^ | No | No | No | No |
|  | taselisib | II | LORELEI doi:10.1016/  S1470-2045(19)30334-1 | 334 |  | - letrozole + taselisib - letrozole + Placebo | Fixed dose | - ORR PIK3Cam - ORR all | - ORR PIK3CAwt | No | Yes,  Type 1 or 2 diabetes requiring antihyperglycemic medication | No | NA | No | No | No | No |
|  | capivasertib | I | STAKT doi:10.1158/  1078-0432.CCR-19-3053 | 36 | ER+ | - Capivasertib - placebo | Fixed dose | - changes from baseline in AKT pathway markers pPRAS40, pGSK3β, and Ki67 | - Safety and tolerability - Other AKT pathway markers | No | Yes,  patients with type 1 or 2 diabetes mellitus (irrespective of management), fasting glucose ≥7 mmol/L, or glycated hemoglobin (HbA_1c_) ≥64 mmol/mol or ≥8% | Yes | BMI cohort A) 27.3 kg/m^2^, B) 28.8 kg/m^2^, C) 31.5 kg/m^2^, D)27.4 kg/m^2^ | No | No | Mean weight and height of each cohort | Yes, without subgroup analysis according to weight/BMI |
|  | ipatasertib | II | FAIRLANE doi:10.1093/  annonc/mdz177 | 151 | TNBC | °paclitaxel + ipatasertib | Fixed dose | - pCRin ITT - pCR in PTEN-low | - pCR in PIK3ca/AKT1/PTEN altered tumors - Response rate MRI | No | History of Type I or Type II diabetes mellitus requiring insulin | No | NA | No | No | No | No |
| Metastatic | everolimus | II | NCT00426530 doi:10.1007/s10549-010-1260-x | 47 | HER2+ | - everolimus + trastuzumab + vinorelbine | Fixed dose | - DLT | - safety - RDI - ORR - PK | No | No | No | NA | No | No | No | Yes, without subgroup analysis according to weight/BMI |
|  |  | II | XENERA doi:10.1186/  s13058-023-01649-w | 103 | HR+ HER2- | - Everolimus + exemestane + xentuzumab - Everoliumus + exemestane + PCB | Fixed dose | - PFS | - OS - DCR | No | No | No | NA | No | No | No | No |
|  |  | II | NCT00570921 doi:10.1007/  s10549-013-2810-9 | 33 | ER+ | - Fulvestrant + everolimus | Fixed dose | - TTP | - ORR - CBR - safety - Biomarkers | No | No | No | NA | No | No | No | No |
|  |  | I | NCT03154281 doi:10.1002/  cam4.6475 | 14 pts, 1 BC | TNBC | - Everolimus + niraparib | Fixed dose | - Safety and tolerability MTD RP2D | - ORR - toxicity | No | No | No | NA | No | No | No | No |
|  |  | II | BOLERO 6 doi:10.1001/  jamaoncol.2018.2262 | 309 | ER+ HER2- | - Everolimus + exemestane - Everolimus - Capecitabine | Fixed dose | - PFS arm A vs B | - PFS arm A vs Cs - Safety | No | No | No | NA | No | No | No | No |
|  |  | II | TAMRAD doi:10.1200/  JCO.2011.39.0708 | 57 | HR+ HER2- | - Tamoxifen + everolimus - Tamoxifen | Fixed dose | - CBR 6m | - TTP - OS - Toxicity - CBR | No | No | No | NA | No | No | No | No |
|  |  | II | PEARL  doi:10.1038/  s41523-021-00331-8 | 46 | ER+ HER2- | - Everolimus + exemestane | Fixed dose | - Association early response and PFS | - ctDNA analysis | No | Yes,  Uncontrolled diabetes as defined by fasting glucose ≥ 1.5 x ULN | No | NA | No | No | No | No |
|  |  | Ib/II | NCT02123823 doi:10.1186/  s13058-020-01382-8 |  | HR+ HER2- | - Everolimus + exemestane + xentuzumab - Everoliumus + exemestane | Fixed dose | - MTD RP2D PFS | - Phase I:  MTD - Phase II:  PFS TTP time to OR ORR DoR | No | No | No | NA | No | No | No | No |
|  |  | I/II | TRINITI-1 doi:10.1158/1078-0432.CCR-20-2114 | 104 | HR+ HER2- | - Exemestane + ribociclib + everolimus | Fixed dose | - CBR | - PFS - OS - overall response - rate - safety | No | No | No | NA | No | No | No | No |
|  |  | II | MIRACLE doi:10.1001/  jamaoncol.2021.3428 | 199 | ER+ | - everolimus + letrozole - letrozole | Fixed dose | - PFS | - ORR - CBR - DoR - OS | No | No | No | NA | No | No | No | No |
|  |  | II | NCT01797120 doi:10.1200/  JCO.2017.76.9331 | 131 | ER+ HER2- | - Fulvestrant + everolimus - Fulvestrant + PCB | Fixed dose | - PFS | - ORR - CBR | No | Yes,  Uncontrolled diabetes as defined by fasting serum glucose >1.5 x ULN | No | NA | No | No | No | No |
|  |  | II | LCCC1025 doi:10.1007/  s10549-018-4852-5 | 32 | HER2+ | - everolimus + trastuzumab + vinorelbine | Fixed dose | - Intracranial RR | - CBR CNS - extracranial RR - TTP - OS | No | Yes,  uncontrolled diabetes, defined as fasting serum glucose >1.5 x ULN (Note: Optimal glycemic control should be achieved before starting trial therapy) | No | NA | No | No | No | No |
|  |  | II | NCT01127763 doi:10.1186/  bcr3634 | 25 | TNBC | - Everolimus + carboplatin | Fixed dose | - CBR | - OS - PFS | No | Yes,  uncontrolled diabetes as defined by fasting serum glucose >1.5 x ULN | No | NA | No | No | No | No |
|  |  | I | NCT00930475 no doi available; Anticancer Research August 2012, 32 (8) 3435-3441 | 15 | TNBC | - Everolimus + carboplatin | Fixed dose | - MTD DLT | - AE | No | No | No | NA | No | No | No | No |
|  |  | I/II | NCT01939418 doi:10.7150/  jca.24035 | 23 | TNBC | - Gemcitabine + cisplatin + everolimus | Fixed dose | - Phase I:  MTD Phase II:  PFS Toxicity | - OS - ORR - Toxicity profile | No | Yes,  uncontrolled diabetes mellitus | No | NA | No | No | No | No |
|  |  | II | VicTORia doi:10.1007/  s10549-019-05280-2 | 133 | HER2- | - Chemotherapy + vinorelbine - Chemotherapy + vinorelbine + everolimus | Fixed dose | - PFS | - PFS 6m - OS - ORR - safety | No | No | Yes | BMI cohort A) 25.4, B) 27.4 kg/m2 | No | No | No | No |
|  |  | II | MANTA doi:10.1001/  jamaoncol.2019.2526 | 333 | HR+ | - fulvestrant - Fulvestrant + vistusertib daily - fulvestrant + vistusertib intermittently - Fulvestrant + everolimus | Fixed dose | - PFS | - OS - ORR - CBR - DoR - Safety | No | Yes, type 1 diabetes or uncontrolled type 2 diabetes | No | NA | No | No | No | No |
|  |  | II | SWISH  doi:10.1016/  S1470-2045(17)30109-2 | 92 | HR+ HER2- | - everolimus + exemestane + dexamethasone wash | Fixed dose | - Grade II stomatitis | / | No | Yes,  Uncontrolled diabetes mellitus as defined by HbA1c >8% despite adequate therapy. Patients with a known history of impaired fasting glucose or diabetes mellitus (DM) may be included, however blood glucose and antidiabetic treatment must be monitored closely throughout the trial and adjusted as necessary; | No | NA | No | No | No | No |
|  |  | II | DESIREE doi:10.1016/  j.esmoop.2022.100601 | 160 | HR+ HER2- | - Everolimus + exemestane | Fixed dose | - stomatitis G >/=2 rate at 12w | - stomatitis G >/=2 rate at 24w °cumulative rate of stomatitis °CBR °RTDI °Time to onset stomatitis G >/= 2 °safety °QoL | No | No | Yes | Yes, cohort A) 44% <25 kg/m2, B)51% <25 kg/m2, C) 47% <25 kg/m2 | No | No | No | No |
|  |  | I | NCT02152943 doi:10.1158/  1078-0432.CCR-20-2878 | 32, 26 BC | HR+ HER+ BC and other solid tumors | - Letrozole + everolimus + trastuzumab | Fixed dose | - DLT MTD RP2D | - Safety | No | Yes,  Uncontrolled diabetes mellitus (HbA1c) > 8% despite adequate therapy; patients with a known history of impaired fasting glucose or diabetes mellitus (DM) may be included | No | NA | No | No | No | No |
|  |  | II | NCT01698918 doi:10.1001/  jamaoncol.2018.0060 | 202 | ER+ HER2- | - Everolimus + letrozole At progression: exemestane + everolimus | Fixed dose | - PFS | - OS - ORR - CBR | No | No | No | NA | No | No | No | No |
|  |  | I | NCT02120469 doi:10.1186/  s13058-019-1202-4 | 27 | TNBC | - Eribulin + everolimus | Fixed dose | - Safety and toxicity RP2D | - ORR - PFS | No | No | No | NA | No | No | No | No |
|  |  | II | NCT02015559 doi:10.1177/  1758835920967259 | 32 | HR+ | - everolimus - everolimus + mouth wash | Fixed dose | - Rate of G 1-4 stomatitis | - Rate of stomatitis by OMAS - Rate of everolimus adjustments/discontinuation due to stomatitis | No | Yes,  Uncontrolled diabetes (HbA1c) > 8% despite adequate therapy; patients with a known history of impaired fasting glucose or diabetes mellitus (DM) may be included | No | NA | No | No | No | No |
|  |  | I | EVESOR doi:10.1007/  s00280-023-04508-9 | 43 pts, 5 BC |  | - everolimus + sorafenib | Fixed dose | - RP2D | - PK/PD - ORR - PFS | No | No | No | NA | No | No | Mean body weight | yes, weight didnt have influence on PK parameters |
|  |  | II | LEO trial doi:10.1016/  j.ejca.2020.11.044 | 137 | HR+ HER2- | - everolimus + letrozole + leuprorelin - letrozole + leuprorelin | Fixed dose | - PFS | - OS - ORR - CBR - Safety | No | No | No | NA | No | No | No | No |
|  |  | II | NCT02291913 doi:10.1016/  j.clbc.2019.06.005 | 47 | HR+ HER2- | - Everolimus + ET | Fixed dose | - PFS | - ORR - CBR - OS | No | No | No | NA | No | No | No | No |
|  |  | II | Jones et al doi:10.1634/  theoncologist.2018-0340 | 100 | HR+ | - AI + everolimus + Moutwash 1 vs AI + everolimus + Mouthwash 2 | Fixed dose | - incidence of grade ≥2 stomatitis and related events | - incidence of AEs (all grades); percentage of patients requiring dose interruption, dose reduction, or discontinuation of everolimus because of toxicity; and evaluation of the impact of mouth rinse on the duration and severity of mIAS, as assessed by the OSDQ. | No | Yes,  Patients with uncontrolled diabetes mellitus (DM) as defined by hemoglobin A1c >8% despite adequate therapy were not eligible | No | NA | No | No | No | No |
|  |  | II | NCT01627067 doi:10.1007/  s10637-018-0700-z | 22 | HR+ HER2- | - exemestane + everolimus + metformine | Fixed dose | - PFS | - OS - DOR | Yes,  yes, BMI >/= 25 kg/m2 | No | No | NA | No | No | No | No |
|  |  | II | NCT0123659 doi:10.1016/  j.clbc.2017.09.004 | 72 | HR+ HER2- | - everolimus + letrozole | Fixed dose | - ORR | - DCR - PFS - OS - Safety | No | No | No | NA | No | No | No | No |
|  |  | II | Vidal et al  doi:10.1016/  j.clbc.2017.03.003 | 18 | HER2- | - everolimus + capecitabine | Fixed dose | - MTD PK | - PFS - OS - CBR - Safety | No | No | No | NA | No | No | No | Yes, without subgroup analysis according to weight/BMI |
|  |  | I | Basho et al doi:10.1001/  jamaoncol.2016.5281 | 52 | TNBC | - everolimus + bevacuzimab + liposomal doxorubicin | Fixed dose | - Safety  ORR | - CBR - PFS | No | No | No | NA | No | No | No | No |
|  |  | II | NCT00915603 doi:10.1007/  s10549-015-3599-5 | 113 | HER2- | - Paclitaxel + bevacuzimab + everolimus - paclitaxel + bevacuzimab + PCB | Fixed dose | - PFS | - ORR - OS - Safety | No | No | No | NA | No | No | No | No |
|  |  | II | J2101  doi:10.1007/  s10549-013-2689-5 | 55 | HER2+ | - everolimus + trastuzumab +paclitaxel | Fixed dose | - ORR | - PFS - OS - CBR - Safety | No | No | No | NA | No | No | No | No |
|  |  | II | RADAR doi:10.1007/  s00432-013-1518-x | 89 | HER2- | - Everolimus 8w => everolimus continue - Everolimus 8w => placebo | Fixed dose | - TTP | - TTP in responders - ORR - CBR - bone M+ related event - pain - safety - Compliance | No | Yes,  uncontrolled diabetes with a fasting blood glucose level above 120 mg/dl. | No | NA | No | No | No | No |
|  |  | I | Moulder et al doi:10.1002/  cncr.26571 | 15 | All BC subtypes | - Docetaxel + everolimus | Fixed dose | - MTD DLT | - PK | No | No | No | NA | No | No | No | Yes, without subgroup analysis according to weight/BMI |
|  |  | Ib | André et al  doi:10.1200/  JCO.2009.27.8549 | 33 | HER2+ | - Everolimus + trastuzumab + paclitaxel | Fixed dose | - DLT | - RDI - ORR - PK | No | No | No | NA | No | No | No | Yes, without subgroup analysis according to weight/BMI |
|  |  | II | NCT00255788 doi:10.1200/  JCO.2008.21.3033 | 49 | All BC subtypes | - Everolimus | Fixed dose | - ORR | - Safety | No | No | No | NA | No | No | No | No |
|  | Temsirolimus | I | NCT00600496  doi:10.1007/  s10637-017-0459-7 | 80 | Not only BC | - selumetinib + erlotinib - selumetinib + temsirolimus | Fixed dose | - safety - tolerability | - PK - MTD - ORR | No | Yes, uncontrolled diabetes | No | NA | No | No | No | Yes, without subgroup analysis according to weight/BMI |
|  |  | I | NCT01552434 doi:10.1093/  oncolo/oyad158 | 47 | Not only BC | - bevacizumab + temsirolimus + valproic acid | Fixed dose | - safety tolerability - MTD | - ORR | No | No | No | NA | No | No | No | No |
|  |  | Ib | NCT0098263 doi:10.1007/  s11523-014-0309-x | 20 | Not only BC | - pegylated liposomal doxorubicin + temsirolimus | Fixed dose | - MTD - RP2D | - Safety and toxicity - PK - ORR - use of PET-CT to identify those of use of temsirolimus | No | No | No | NA | No | No | No | Mentioned in objectives, though not reported in results |
|  |  | I | Ma et al  doi:10.1007/  s10549-013-2528-8 | 26 | All BC | - temsirolimus + cixutumumab | Fixed dose | - MTD - DLT | - Safety profile - ORR | No | yes,  diabetes requiring oral hypoglycemics or insulin | No | NA | No | No | No | No |
|  |  | I | Moroney et al doi:10.1158/  1078-0432.CCR-12-1158 | 136 | Not only BC | - temsirolimus + bevacuzimab + liposomal doxorubicin | Fixed dose | - Safety - MTD - DLT | - ORR | No | No | No | NA | No | No | No | No |
|  |  | II | Fleming et al doi:10.1007/  s10549-011-1910-7 | 31 | All BC | - temsirolimus | Fixed dose | - CBR - Safety | / | No | No | No | NA | No | No | No | No |
|  |  | II | Chan et al doi:10.1200/  JCO.2005.66.130. | 109 | All BC | - temsirolimus | Fixed dose | - ORR | - CBR - TTR - DoR - TTP - PK | No | No | No | NA | No | No | No | higher body weight was associated with higher temsirolimus concentrations |
|  | Alpelisib | Ib | NCT02077933 doi:10.1016/  j.ejca.2021.03.042 | 79 | HR+ HER2- | °Alpelisib + everolimus °Alpelisib + everolimus + exemestane | Fixed dose | - MTD RP2D | - ORR CBR DCR DoR | No | Yes, all diabetes patients | No | NA | No | No | No |  |
|  |  | Ib | NCT02051751 doi:10.18632/oncotarget.25854 | 19 | Not only BC | Alpelisib + paclitaxel | Fixed dose | - MTD RP2D | - Safety | No | Yes, requiring insulin treatment and/or with clinical signs | No | NA | No | No | No |  |
|  |  | II | PIKNIC doi:10.1158/  2159-8290.CD-21-1696 | 43 | ER+ HER2- and TNBC | Alpelisib | Fixed dose | - ORR | - CBR PFS DoR | No | Yes, type I diabetes mellitus or uncontrolled type II diabetes mellitus | yes | yes, ER+ 25,6 kg/m2, TNBC 27,5 kg/m2 | No | No | No |  |
|  |  | I | NCT02167854 doi:10.1158/  1078-0432.CCR-21-0047 | 10 | HER2+ | Alpelisib + trastuzumab | Fixed dose | - MTD RP2D | - Toxicity ORR | No | Yes, uncontrolled diabetes | No | NA | No | No | No |  |
|  |  | II | BYLieve doi:10.1016/  S1470-2045(21)00034-6 | 127 | HR+ | Alpelisib | Fixed dose | - ORR | - PFS ORR PFS after second line DoR OS safety and tolerability | No | Yes, plasma glucose level of more than 140 mg/dL (7·7 mmol/L) and glycated haemoglobin A1c (HbA1c) no more than 6·4% or 47 mmol/mol | Yes | yes, mean BMI 26,1  kg/m2 | No | No | No |  |
|  |  | Ib | NCT01791478 doi:10.1158/  1078-0432.CCR-16-0134 | 26 | ER+ HER2- | alpelisib + letrozole | Fixed dose | - Safety MTD | - CBR - PFS - ORR | No | Yes, clinically-manifested diabetes mellitu | No | NA | No | No | No | No |
|  |  | I | NCT01219699 doi:10.1001/  jamaoncol.2018.4475 | 221 | not only BC | A) alpelisib B) alpelisib + fulvestrant | Fixed dose | - MTD - RP2D | - Safety and tolerability - PK | No | Yes, diabetes mellitus (treated and/or symptomatic, or with FPG ≥ 140 mg/dL [7.8 mmol/L]), a history of gestational diabetes mellitus, or documented steroid-induced diabetes mellitus | No | NA | No | No | No | Yes, without subgroup analysis according to weight/BMI |
|  |  | Ib | B-YOND doi:10.1158/  1078-0432.CCR-20-1008 | 29 | HR+ HER2- | °tamoxifen + goserelin acetate + alpelisib °tamoxifen + goserelin acetate + buparlisib | Fixed dose | - MTD - RP2D | - PFS - ORR - CBR - Safety | No | No | No | NA | No | No | No | No |
|  |  | I/II | Sharma et al doi:10.1158/  1078-0432.CCR-20-4879 | 43 | HER2- | Alpelisib + nab-paclitaxel | Fixed dose | - RP2D - ORR | - safety - PK - PFS - PIK3CAm associations w outcome | No | Yes,  uncontrolled diabetes mellitus [fasting plasma glucose (FPG) level >140 mg/dL (7.8 mmol/L) or glycosylated hemoglobin (HbA1C) >8%] | No | NA | No | No | No | Yes, without subgroup analysis according to weight/BMI |
|  | Buparlisib | II | NCT01790932 doi:10.1186/  s13058-020-01354-y | 50 | TNBC | Buparlisib | Fixed dose | - CBR | - PFS - OS - toxicity |  | Yes, if poorly controlled | No | NA | No | No | No | No |
|  |  | Ib/II | NCT01132664 doi:10.1007/  s10549-017-4596-7 | 53 | HER2+ | Ib: (brain M): buparlisib + trastuzumab + capecitabine II: Buparlisib + trastuzumab | Fixed dose | - ORR | - safety - tolerability |  | Yes, clinical manifest diabetes mellitus or steroid-induced diabetes mellitus | No | NA | No | No | No | Yes, without subgroup analysis according to weight/BMI |
|  |  | Ib/II | NCT01068483 doi:10.1200/  JCO.2011.36.1360 | 35 | Not only BC | Buparlisib | Fixed dose | - MTD | - safety - tolerability - ORR - PK - PD |  | Yes, clinically manifest diabetes mellitus, including a history of gestational diabetes | No | NA | No | No | No | Yes, without subgroup analysis according to weight/BMI |
|  |  | Ib/II | NCT01626209 doi:10.21873/  anticanres.11212 | 32 | Not only BC | Buparlisib | Fixed dose | - MTD RP2D | - safety - tolerability - PK - ORR |  | No | No | NA | No | No | No | Yes, without subgroup analysis according to weight/BMI |
|  |  | Ib | NCT01363232 doi:10.1634/  theoncologist.2019-0297 | 89 | Not only BC | Binimetinib + buparlisib | Fixed dose | - MTD RP2D | - PK - PD - Safety - ORR |  | Yes, all diabetes mellitus | No | NA | No | No | No | Yes, without subgroup analysis according to weight/BMI |
|  |  | I | NCT01339442 doi:10.1158/  1078-0432.CCR-15-1745 | 31 | ER+ | Buparlisib | Fixed dose | - MTD | - PK - ORR - CBR - PFS |  | Ye,s if poorly controlled diabetes mellitus or steroid-induced diabetes mellitus (defined by fasting glucose >120 mg/dL or hemoglobin [Hb] A1c > 7%) | No | NA | No | No | No |  |
|  |  | Ib | NCT01155453 doi:10.1158/  1078-0432.CCR-14-1814 | 113 | Not only BC | Buparlisib + trametinib | Fixed dose | - DLT | - Safety - PK - PD - Efficacy | No | No | No | NA | No | No | No | No |
|  |  | I | NCT01300962 doi:10.1016/ j.clbc.2017.10.014 | 25 | All BC subtypes | Buparlisib + capecitabine | Fixed dose | - DLT MTD AE | - ORR - PK | No | Yes, if poorly controlled diabetes mellitus | No | NA | No | No | No | Yes, without subgroup analysis according to weight/BMI |
|  |  | I | NCT01248494 doi:10.1200/ JCO.2013.54.0518 | 51 | ER+ HER2- | Buparlisib + letrozole | Fixed dose | - Safety and tolerability | - PFD - ORR | No | Yes, clinically manifest diabetes mellitus, | No | NA | No | No | No | Yes, without subgroup analysis according to weight/BMI |
|  |  | II | PIKTAM doi:10.1002/ cam4.3092 | 21 | HR+ HER2- | Buparlisib + tamoxifen | Fixed dose | - PFS | - PFS in subpopulations - OS - ORR - DCR - Safety | No | Yes, if uncontrolled diabetes mellitus | Yes | BMI cohort A) 27.9 kg/m2, B)21.8 kg/m2, C)24 kg/m2 | No | No | No | No |
|  | Capivasertib | II | PAKT trial doi:10.1200/ JCO.19.00368 | 140 | ER+ | Capivasertib placebo | Fixed dose | - PFS | - OS | No | Yes, DM type I or II hbA1c >/= 8% fasting glucose >/= 7,0 mmol/L | No | NA | No | No | No | No |
|  |  | I | NCT04712396 doi:10.1002/ cpdd.1307 | 11 | TNBC | °Paclitaxel + capivasertib °Paclitaxel + placebo | Fixed dose | - PK AE |  | yes, weight between 50-100kg | No | No | NA | No | No | Mean body weight | No |
|  |  | II | FAKTION doi:10.1016/ S1470-2045(22)00284-4 | 183 | healthy | °Capivasertib + itraconazole | Fixed dose | - PFS | - PFS by PIK3CA OS safety | No | Yes, if Hba1c (>8%) and fasting blood glucose of more than 9·3 mmol/L | No | NA | No | No | No | No |
|  |  | I/II | NCT01226316 doi:10.1158/ 1078-0432.CCR-19-3953 | 63 | ER+ HER2- | °Fulvestrant + capivaserib °Fulvestrant + placebo | Fixed dose | - Safety and tolerability RP2D | - ORR DoR PFS | No | Yes, (i) diagnosis of diabetes mellitus type I or II (irrespective of management); (ii) baseline fasting glucose value of ≥7 mmol/L (fasting is defined as no calorific intake for at least 8 hours); and (iii) glycated hemoglobin (HbA1c) >8% (>64 mmol/mol). | No | NA | No | No | No | No |
|  |  | I/II | BEECH  doi:10.1093/ annonc/mdz086 | 148 | ER+ HER2- | Capivasertib | Fixed dose | - Safety and tolerability RP2D PFS | - ORR PFS DOR | No | Yes, Diagnosis of diabetes mellitus type I or II (irrespective of management). Glycosylated haemoglobin (HbA1C) >8.0% at OR Fasting Plasma Glucose >7.0mmol/L at screening. | No | NA | No | No | No | Yes, without subgroup analysis according to weight/BMI |
|  | Ipatasertib | II | LOTUS doi:10.1007/  s10549-021-06143-5 | 124 | TNBC | °paclitaxel + ipatasertib | Fixed dose | - PFS in ITT PFS in PTEN-low | - OS DOR | No | No | No | NA | No | No | No | No |
| **OTHER** | | | | | | | | | | | | | | | | | |
| Metastatic | margetuximab | I | NCT01148849 doi:10.1093/ annonc/mdx002 | 66 | HER2+ | °Margetuximab 0,1-6mg/kg 3w/1off  °Margetuximab 10-18 mg/kg q3w | Weight-based dose | - AEs | - DLT - PK - drug antibodies - ORR - DoR - PFS - OS - translatoinal | No | No | No | NA | No | No | No | Yes, without subgroup analysis according to weight/BMI |
|  |  | II | NCT04262804 doi:10.21037/tbcr-22-35 | 123 | HER2+ | °Margetuximab plus chemotherapy °Trastuzumab plus chemotherapy | Weight-based dose | - PFS | - OS - PFS by investigator - ORR - DoR - CBR - FcgammaR | No | No | No | NA | No | No | No | No |

*PK: pharmacokinetics, DLT: dose limiting toxicities, AEs: adverse events, FEC: ###, AI: aromatase inhibitor, ORR: objective response rate, pCR: pathological complete response, RCB: residual cancer burden , BCS: breast conserving surgery, CCCA: complete cell cycle arrest , PRO: patient reported outcome, QoL: Quality of Life, pRB: plasma RB protein, OS: overall survival, CBR: clinical benefit rate, PFS: progression free survival , DCR: disease control rate , TTP: time to progression, DoR: duration of response, CNS: central nervous system, LHRH: luteinizing hormone release hormone, PEPI: preoperative endocrine prognostic index, RP2D: recommended phase 2 dose , iORR: intracranial ORR , MDASI: MD Anderson Symptom Inventory Instrument, ET: endocrine treatment, iDFS: invasive disease free survival, dDFS: distant disease free survival , TTR: time to response, TFS: time to failure of strategy, BOR: best overall response, EC: epirubicin + cyclophosphamide, TILs: tumor infiltrating lymphocytes , AR: androgen receptor, TNBC: triple negative breast cancer, cpCR: comprehensive pathological complete response*
